# Ferroptosis‐modulating small molecules for targeting drug‐resistant cancer: Challenges and opportunities in manipulating redox signaling

**DOI:** 10.1002/med.21933

**Published:** 2023-01-19

**Authors:** Solveigh C. Koeberle, Anna P. Kipp, Hermann Stuppner, Andreas Koeberle

**Affiliations:** ^1^ Michael Popp Institute, Center for Molecular Biosciences Innsbruck (CMBI) University of Innsbruck Tirol Innsbruck Austria; ^2^ Department of Molecular Nutritional Physiology, Institute of Nutritional Sciences Friedrich Schiller University Jena Thüringen Jena Germany; ^3^ Unit of Pharmacognosy, Institute of Pharmacy, Center for Molecular Biosciences Innsbruck (CMBI) University of Innsbruck Tirol Innsbruck Austria

**Keywords:** drug‐resistant cancer, ferroptosis, natural product, small molecule, structural requirement

## Abstract

Ferroptosis is an iron‐dependent cell death program that is characterized by excessive lipid peroxidation. Triggering ferroptosis has been proposed as a promising strategy to fight cancer and overcome drug resistance in antitumor therapy. Understanding the molecular interactions and structural features of ferroptosis‐inducing compounds might therefore open the door to efficient pharmacological strategies against aggressive, metastatic, and therapy‐resistant cancer. We here summarize the molecular mechanisms and structural requirements of ferroptosis‐inducing small molecules that target central players in ferroptosis. Focus is placed on (i) glutathione peroxidase (GPX) 4, the only GPX isoenzyme that detoxifies complex membrane‐bound lipid hydroperoxides, (ii) the cystine/glutamate antiporter system X_c_^−^ that is central for glutathione regeneration, (iii) the redox‐protective transcription factor nuclear factor erythroid 2‐related factor (NRF2), and (iv) GPX4 repression in combination with induced heme degradation via heme oxygenase‐1. We deduce common features for efficient ferroptotic activity and highlight challenges in drug development. Moreover, we critically discuss the potential of natural products as ferroptosis‐inducing lead structures and provide a comprehensive overview of structurally diverse biogenic and bioinspired small molecules that trigger ferroptosis via iron oxidation, inhibition of the thioredoxin/thioredoxin reductase system or less defined modes of action.

## 1 INTRODUCTION

Ferroptosis, an alternative cell death program to apoptosis, promises access to anticancer strategies that selectively kill malignant cells and are effective against aggressive, drug‐resistant tumors.^1^, ^2^ Challenges in targeting ferroptosis arise from interference with redox‐dependent signaling cascades whose regulatory mechanisms, unlike kinase signaling cascades, are less investigated and not completely understood. Cutting‐edge reports on chemical probes that inhibit central proteins in ferroptosis, that is, glutathione peroxidase (GPX) 4 and system X_c_^−^, provided essential insights into ferroptotic pathways and disclosed additional targets.^2^, ^3^, ^4^ Recent review articles add to the growing literature that links ferroptosis to human disease and emphasize the critical involvement of redox‐dependent mechanisms.^1^, ^5^, ^6^, ^7^, ^8^, ^9^, ^10^ While the induction of ferroptosis is considered a promising strategy to fight aggressive, metastatic, and therapy‐resistant cancer, current drug development is hampered by the rapidly increasing but still insufficiently structured knowledge about molecular mechanisms and structural requirements of ferroptosis‐inducing small molecules.

Ferroptosis is a strictly regulated necrotic form of programmed cell death (PD) that differs on a cellular and molecular level from other cell death programs like apoptosis, anoikis, autophagy, and regulated necrosis (necroptosis, pyroptosis).^11^ Morphological signs of ferroptosis are visible in but not limited to mitochondria, which decrease in size, gain membrane density, and lose clearly structured mitochondrial cristae. In addition, large membrane blebs are frequently detected.^12^ Ferroptotic cell death critically depends on the iron‐dependent peroxidation of membrane lipids.^13^, ^14^, ^15^, ^16^ While moderate membrane peroxidation also occurs in other cell death pathways, excessive phospholipid peroxidation is a hallmark of ferroptosis.

**Figure 1:**
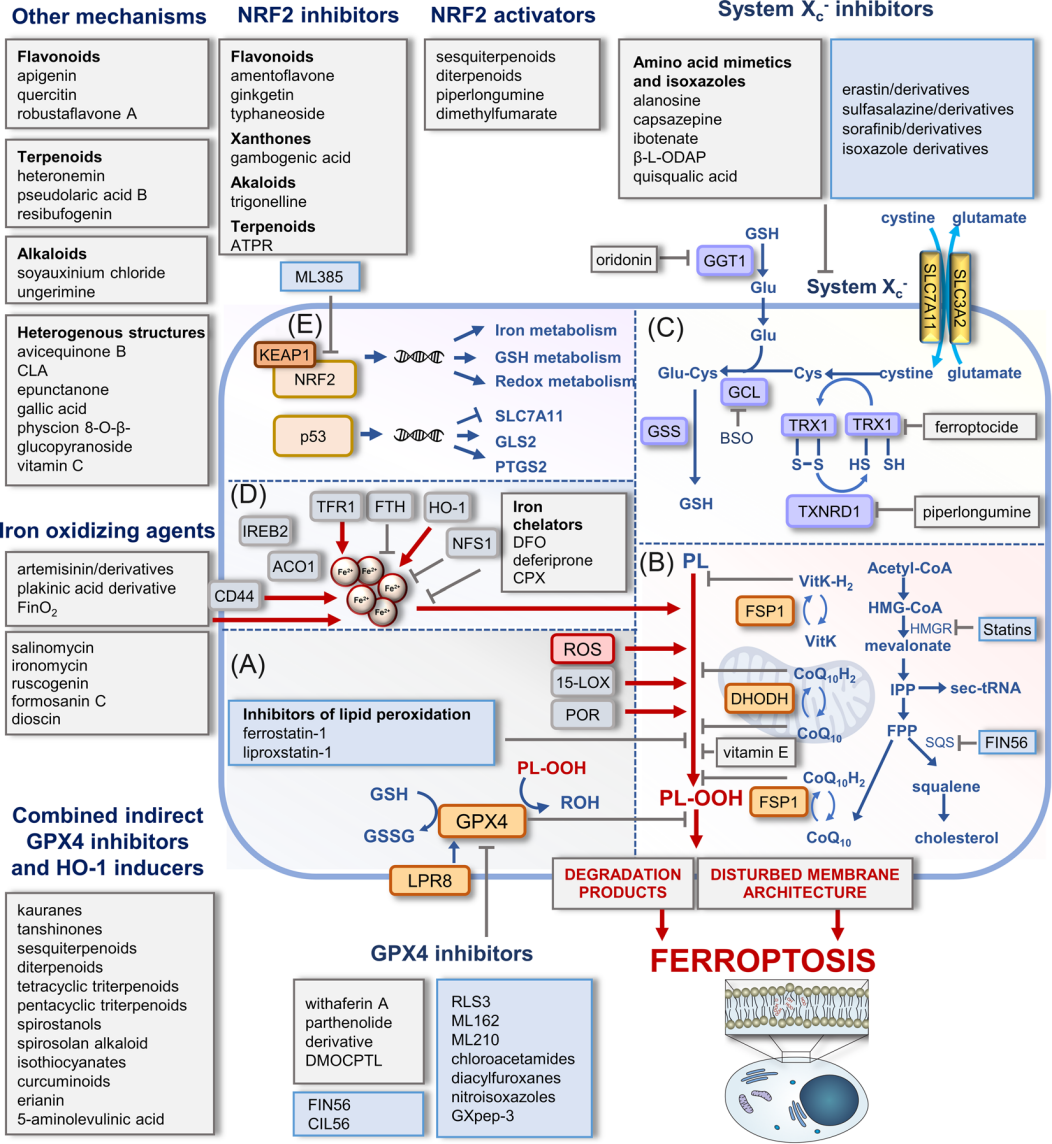
Major metabolic and regulatory pathways in ferroptosis targeted by small molecules. (A) Membrane peroxidation in ferroptosis depends on enzymatic and nonenzymatic mechanisms. LOX isoenzymes and oxidoreductases (POR) with Fe^2+^/Fe^3+^ in their active center introduce oxygen into polyunsaturated fatty acids (PUFAs), and free metal ions, in particular Fe^2+^, convert hydrogen peroxide into hydroxyl radicals via the Fenton reaction. GPX4 reduces lipid hydroperoxides, counteracts ferroptosis, and relies on the biosynthesis and regeneration of its substrate glutathione. (B) Additional protection against membrane peroxidation offers endogenous lipophilic radical traps such as ubiquinol and vitamin K (VitK), which are regenerated by FSP1 and DHODH in the cytosol and mitochondria, respectively. The mevalonate pathway is central for the biosynthesis of (i) cholesterol, (ii) the lipophilic radical trap CoQ10, and (iii) selenocysteine transfer RNA (sec‐tRNA), which inserts selenocysteine into GPX4. (C) System X_c_^−^ regulates cellular GSH levels by importing cystine in exchange for glutamate (Glu). Intracellular cystine is reduced to cysteine, which subsequently enters GSH biosynthesis and serves as a cofactor for GPX4. (D) Labile iron levels are kept within narrow thresholds by co‐ordinated regulation of iron uptake via the transferrin receptor and iron storage within ferritin. Other factors in the control of labile iron levels sequester iron into iron–sulfur clusters (NSF1) or liberate iron from heme oxygenase‐1 (HO‐1). The transmembrane glycoprotein CD44 mediates the endocytosis of iron‐bound hyaluronate. The scheme illustrates points of attack of selected small molecules that induce ferroptosis, with a focus on drug candidates, tool compounds, and natural products. The color of the boxes distinguishes between biogenic/bioinspired (gray) and synthesized small molecules (blue). (E) Central ferroptotic genes are under the control of the transcription factors NRF2 and p53. Mitochondrium in (B) was adapted from “Resting Metabolic Activity vs. Stimulated Metabolic Activity,” by BioRender.com (2020). Retrieved from https://app.biorender.com/biorender-templates. 2,2′‐BP, 2,2′‐bipyridine; 5‐ALA, 5‐aminolevulinic acid; ACO1, aconitase 1; ATPR, 4‐amino‐2‐trifluoromethyl‐phenyl retinate; BHT, butylhydroxy toluol; BSO, *l* ‐buthionine‐*S,R*‐sulfoximine; CLA conjugated linolenic acids; CPX, ciclopirox; DFO, DHODH, dihydroorotate dehydrogenase; deferoxamine; DMF, dimethyl fumarate; FPP, farnesyl pyrophosphate; FSP1, ferroptosis suppressor protein 1; FTH, ferritin heavy chain; GCL, glutamate cysteine ligase; GGT1, γ‐glutamyl transpeptidase 1; GLS2, glutaminase 2; GPX4, glutathione peroxidase 4; GSH, reduced glutathione; GSS, glutathione synthetase; GSSG, glutathione disulfide; HMG‐CoA, 3‐hydroxy‐3‐methylglutaryl‐coenzyme A; HMGR, 3‐hydroxy‐3‐methylglutaryl‐CoA reductase; IPP, isopentenyl pyrophosphate; IREB2, iron responsive element binding protein 2; KEAP1, kelch‐like ECH‐associated protein 1; LOX, lipoxygenase; NFS1, mitochondrial cysteine desulfurase; NRF2, nuclear factor erythroid 2‐related factor 2; PL, phospholipid; PL‐OOH, phospholipid hydroperoxide; PL‐OH, phospholipid alcohol; POR, cytochrome P450 oxidoreductase; ROS, reactive oxygen species; SQS squalene synthase; TFR, transferrin receptor; TRX, thioredoxin; TXNRD1, thioredoxin reductase 1; β‐*l*‐ODAP, β*‐N*‐oxalyl*‐l‐*α‐β‐diaminopropionic acid. [Color figure can be viewed at wileyonlinelibrary.com]

Phospholipids with arachidonic acid (20:4) and adrenic acid (22:4) are preferentially converted into hydroperoxides, with phosphatidylethanolamines seemingly more prone to oxidative modification than other phospholipid classes.^11^, ^13^ Lipid hydroperoxides subsequently degrade to highly reactive metabolites like malondialdehyde (MDA), 4‐hydroxynonenal, and other Michael acceptors^11^, ^17^ that covalently bind to cysteine‐containing stress sensors and likely contribute to membrane dysfunction in ferroptosis (Figure 1). The integrity of cellular membranes is reduced during ferroptosis by microperforations and microruptures, potentially as a direct result of lipid peroxidation, though the exact mechanisms remain obscure.^18^, ^19^ Ferroptotic pathways are engaged in manifold pathologies like degenerative diseases of the central nervous system, organ injury as well as cancer, and emerging evidence suggests that their targeting might help to overcome drug resistance in anticancer therapy.^1^, ^5^, ^11^, ^20^, ^21^ The number of proteins known to participate in ferroptosis is continuously increasing, as is the information about small molecules that interfere with ferroptotic pathways.

Research on designed ligands that trigger ferroptosis is still in its infancy, and many questions remain unanswered: How to selectively modify and fine‐tune redox‐dependent signaling cascades with small molecules? How to design target‐selective ferroptosis inducers? Are multitarget compounds of advantage? Which ferroptotic pathways are predestined targets against therapy‐resistant cancer? How does ferroptosis achieve selective lethality of cancer cells? What underlies the synergism between ferroptosis inducers and conventional chemotherapeutics? The rapidly increasing number of small molecules that trigger ferroptosis will help to answer these questions when channeled into targeted drug discovery and design. Previous review articles addressed the molecular mechanisms and signaling pathways in ferroptotic cell death and, in this context, referred to ferroptosis‐inducing screening hits and synthetic derivatives, phytochemicals, or tool compounds, without in‐depth discussing biomedical and medicinal chemical aspects.^5^, ^10^, ^16^, ^21^, ^22^, ^23^, ^24^, ^25^, ^26^, ^27^, ^28^, ^29^, ^30^, ^31^, ^32^, ^33^ Here, we address ferroptosis from the small molecule inducers’ point of view and provide a comprehensive overview of synthesized, biogenic and bioinspired compounds from diverse sources that induce ferroptosis through mechanisms related to redox signaling, that is, by targeting GPX4, system X_c_^−^, nuclear factor erythroid 2‐related factor 2 (NRF2), and selected NRF2 target genes. We critically discuss ferroptosis‐inducing chemical probes and drug candidates with regard to their structural requirements, molecular mechanisms, and ferroptotic profiles. Moreover, we highlight challenges in proferroptotic drug design and development, propose overarching modes of action, and categorize less‐defined ferroptosis inducers based on structural and functional considerations. On this basis, we propose likely modes of action for less‐defined ferroptosis inducers and highlight challenges in proferroptotic drug design and development.

## 2 POTENTIAL TARGETS IN FERROPTOSIS RELATED TO REDOX SIGNALING

### 2.1 Membrane peroxidation and iron metabolism

Membrane peroxidation in ferroptosis is mediated by enzymatic and nonenzymatic mechanisms.^34^ Lipoxygenases (LOXs), which have Fe^2+^/Fe^3+^ in their active center, introduce oxygen into polyunsaturated fatty acids (PUFAs) in a controlled enzymatic reaction with clear regio‐ and stereospecificity that differs between isoenzymes. With the exception of 15‐LOX that also accepts membrane phospholipids, human LOX isoenzymes (5‐LOX, 12‐LOX) are limited to nonesterified PUFAs as substrates.^11^ In addition to 15‐LOX,^35^ cytochrome P450 oxidoreductase and NADH‐cytochrome b5 reductase 1 participate in enzymatic membrane peroxidation during ferroptosis.^18^, ^36^ Nonenzymatic peroxidation of membrane‐bound PUFAs, on the other hand, is associated with the availability of free redox‐active metal ions, that is, ferrous iron, that converts hydrogen and alkyl peroxides into hydroxyl and alkoxy radicals via the Fenton reaction, respectively.^5^ The import, export, and storage of iron, as well as the turnover and control of the labile iron pool, have, therefore, a strong impact on ferroptosis sensitivity, and iron chelators that decrease the availability of free iron efficiently inhibit ferroptotic cell death.^37^

Iron metabolism is orchestrated by multiple transcription factors, with many of them being linked to ferroptosis, including transcription factor NRF2, yes‐associated protein 1 (YAP1), heat shock transcription factor 1, and hypoxia‐induced factor (HIF) 1/2.^37^ To maintain labile iron levels despite elevated intracellular iron concentrations, cells engage two iron regulatory proteins, IRP1 and IRP2, that tightly coordinate the expression of transferrin receptor (responsible for iron uptake) and ferritin (an iron storage protein).^38^ Sequestering iron into iron–sulfur cluster (ISC) is another efficient cellular strategy to prevent iron‐mediated Fenton reaction and subsequent lipid peroxidation.^39^, ^40^ The biosynthesis of ISCs relies on sulfur supply by mitochondrial cysteine desulfurase (NFS1), which has recently been discovered to protect from ferroptosis.^39^ Suppression of NFS1 activates the canonical iron starvation response and accordingly increases labile iron levels by inducing transferrin receptor expression and decreasing ferritin protein levels. Two other ISC‐modulating proteins, frataxin and CDGSH iron–sulfur domain‐containing protein 2 are upregulated in diverse tumors, most likely to protect cancer cells from ferroptosis by decreasing free iron levels.^41^ Other important factors controlling the cellular abundance of iron are (i) the iron export protein ferroportin, (ii) prominin 2 that mediates the formation of ferritin‐containing multivesicular bodies, (iii) the kinase ataxia telangiectasia mutated, that regulates ferritin availability as well as (iv) the ferrous ion membrane transport protein solute carrier family 11 member 2.^2^

### 2.2 NRF2, p53, and other transcription factors

NRF2 protects from oxidative and electrophilic stress and is linked to enhanced cell survival^42^ and ferroptosis resistance.^43^ Protein levels of NRF2 are tightly controlled by kelch‐like ECH‐associated protein 1 (KEAP1), which forms a complex with NRF2 that is ubiquitinylated by the E3 ubiquitin ligase and subsequently degraded.^44^ Oxidative or electrophilic modifications of cysteine side chains in KEAP1 inhibit NRF2 ubiquitination and allow newly translated NRF2 to translocate into the nucleus and induce NRF2 target gene expression.^45^, ^46^ NRF2 orchestrates the expression of genes involved in iron and redox metabolism,^47^ with many of them (e.g., GPX4, SLC7A11, heme oxygenase [HO]‐1, glutamate‐cysteine ligase catalytic subunit [GCLC], as well as the ferroptosis suppressor protein [FSP] 1 and GTP cyclohydrolase 1 [GCH1]) being closely linked to ferroptosis (Figure 1).^48^, ^49^, ^50^, ^51^

A large set of small molecules modulates NRF2 activation^48^, ^52^ or interferes with other major transcription factors at the heart of ferroptosis, such as p53,^53^ HIF1,^54^ YAP1,^55^, ^56^ activating transcription factor (ATF) 3, activating transcription factor 4 (ATF 4), and specificity protein (SP) 1.^57^ The section “NRF2 inhibitors” discusses those NRF2 inhibitors that have been demonstrated to trigger ferroptosis. There are also few reports on ferroptosis‐inducing compounds that upregulate p53,^58^ a transcription factor that is most commonly mutated in cancer and critically involved in tumorigenesis, metastasis, and drug resistance.^59^ p53 is a tumor suppressor protein that induces redox stress and sensitizes cells to ferroptosis^60^ by coordinating the expression of a large set of target genes including the ferroptosis‐related genes SLC7A11, mitochondrial glutaminase (GLS2), and prostaglandin‐endoperoxide synthase 2 (cyclo‐oxygenase‐2 [COX‐2]).^61^ It should be noted that the effects of p53 are highly dependent on the cell type and context and that p53 activation can both sensitize to and inhibit ferroptosis.^61^, ^62^ Whether small molecules that interfere with HIF1,^63^, ^64^ YAP1,^65^, ^66^, ^67^ ATF3/4, or specificity protein 1 (SP1)^68^ induce cell death through a ferroptotic mechanism is poorly understood.

### 2.3 GPX4 and glutathione regeneration

The selenoprotein GPX4 reduces and thereby detoxifies phospholipid hydroperoxides to the corresponding alcohols (Figure 1).^5^ Deletion or selective inhibition of GPX4, for example, by the tetrahydro‐β‐carboline RSL3 (23), elevates lipid hydroperoxide levels and induces ferroptotic cell death.^69^ GPX4 activity essentially depends on the supply of the cosubstrate GSH and therefore relies on GSH biosynthesis and regeneration as well as the cellular import of cystine via the cystine/glutamate antiporter system X_c_^−^ (Figure 1).^70^ The antiporter (i) consists of two subunits, SLC7A11 (xCT) and SLC3A2 (CD98hc or 4F2hc) that are linked via a disulfide bridge, (ii) regulates the cellular redox status, and (iii) counteracts ferroptosis.^70^ SLC7A11, which is unlike SLC3A2 not shared by other transporters of the heteromeric amino acid transporter family, mediates substrate specificity. After cellular uptake, cystine is reduced to cysteine and subsequently transferred to GSH biosynthesis (Figure 1).

Suppression of GPX4 levels rather than direct inhibition of GPX4 has been suggested as an efficient strategy to promote ferroptosis.^2^ The availability of GPX4 is controlled at different stages. On the on hand, the expression of GPX4 and other key regulators in ferroptosis is orchestrated by transcription factors^2^, ^57^, ^71^: AP‐2γ (TFAP2C), ATF4, SP1, nuclear factor‐κB (NF‐κB), androgen receptor (AR), and CCAAT/enhancer binding protein induce and early growth response protein 1 (EGR1), and sterol regulatory element‐binding protein‐1 repress GPX4 expression. Effects are controversial for NRF2, with deletion and knockdown of the transcription factor either enhancing or diminishing GPX4 transcription.^50^, ^72^, ^73^

The insertion of the catalytically active selenocysteine into GPX4 is regulated by the availability of selenocysteine‐transfer RNA during protein biosynthesis.^74^ The lipoprotein receptor LRP8 (also known as ApoER2) serves as receptor for the uptake of selenoprotein P, a selenium‐rich protein that delivers selenium to extrahepatic tissues.^75^ Selenium, as well as GPX4 levels, accordingly decrease upon knockout of LRP8, the latter being specific for cancer cells.^76^ Conclusively, a cancer‐specific vulnerability toward ferroptotic cell death can be obtained by knock‐out or pharmacological inhibition of LRP8.

Another strategy to adjust cellular GPX4 levels relies on controlled protein degradation following E3 ligase‐dependent ubiquitination.^77^ Ubiquitin‐independent degradation takes place in lysosomes and comprises chaperone‐mediated autophagy, a process that drives the degradation of selective (e.g., oxidized) proteins.^78^ Important for the lysosomal enrichment of GPX4 are the ^124^NVKFD^128^ or ^187^QVIEK^191^ recognition motifs that enable interaction with heat‐shock protein (HSP) 70.^78^

GPX4 enzyme activity is also regulated by posttranslational modification, such as the succination of GPX4 at cysteine 93 (mono‐ and disuccination), which reduces enzymatic activity.^79^ Other posttranslational modifications like SUMOylation and phosphorylation affect GPX4 activity,^77^ but the exact amino acid residues modified and biological consequences remain yet to be elucidated.^77^

### 2.4 Endogenous inhibitors of lipid peroxidation

Several cellular antiferroptotic redox cycles work independent from GPX4 to keep ferroptosis at bay,^11^, ^80^ including (i) FSP1/coenzyme (Co) Q10 (also known as ubiquinone‐10), (ii) dihydroorotate dehydrogenase (DHODH)/CoQ10, and (iii) GCH1/tetrahydrobiopterin (BH4) (Figure 1B). Using NAD(P)H as a redox cofactor, FSP1 reduces CoQ10 at intracellular membranes to ubiquinol (Figure 1B),^5^, ^81^, ^82^, ^83^ which scavenges lipophilic radicals^84^ and regenerates endogenous antioxidants such as tocopherols.^82^ In addition to CoQ10, FSP1 restores the reduced form of vitamin K and derivatives, which are structurally closely related to CoQ10 and represent a group of naphthoquinones with radical‐trapping and antioxidative properties. CoQ10, vitamin K as well as plant‐derived phylloquinone and menaquinone accordingly form (upon reduction) a robust endogenous ferroptosis‐suppressing system.^85^ While FSP1 is located at diverse (intra)cellular membranes, DHODH reduces mitochondrial CoQ10 at the outer side of the inner mitochondrial membrane.^86^ The biosynthesis of CoQ10 can be inhibited by statins that target 3‐hydroxy‐3‐methylglutaryl‐CoA reductase (HMGR), the rate‐limiting enzyme of the mevalonate pathway (Figure 1B).^87^ Similar to endogenous hydroquinones, the widely used ferroptosis inhibitors ferrostatin‐1 and liproxstatin‐1 act as lipophilic radical traps that protect membranes from peroxidation.^88^, ^89^, ^90^ Regeneration to the active reduced form occurs with the consumption of CoQ10.^91^ Other endogenous antiferroptotic factors include the GCH1‐tetrahydrobiopterin (BH4) system that works analogous to FSP1/CoQ10,^92^ inducible nitric oxide (NO) synthase 2 that triggers NO production under proinflammatory conditions and the endosomal sorting complex required for transport that participates in the formation of multilaminar vesicles.^62^

### 2.5 Shaping the PUFA composition of membranes

Ferroptosis sensitivity is associated with PUFA biosynthesis and the incorporation of PUFAs into membrane phospholipids^5^ and decreases with their release from phospholipids by Ca^2+^‐independent phospholipase A_2_.^93^ Long‐chain PUFAs are preferentially found in the *sn*‐2 position of phospholipids, where they are either incorporated during de novo fatty acid biosynthesis by lysophosphatidic acid acyltransferases or through the concerted action of lysophospholipid acyltransferase and phospholipase A_2_ isoenzymes within the remodeling pathway (Land's cycle).^94^ According to their preference for PUFAs as substrates, acetyl‐CoA synthetase long‐chain family member (ACSL) 4, and lysophosphatidylcholine acyltransferase 3 (LPCAT3) increase the PUFA ratio of membranes and render them more susceptible to lipid peroxidation in ferroptosis.^13^, ^95^, ^96^, ^97^ ACSL4 esterifies PUFAs with CoA and the resulting thioesters then serve as the substrate of LPCAT3 that incorporates them into phosphatidylcholines and phosphatidylethanolamines.^98^, ^99^ Pharmacological or genetic inhibition of Acsl4 and Lpcat3 accordingly inhibits ferroptosis in mouse embryonic fibroblasts (MEFs) with tamoxifen‐inducible Gpx4 disruption (Pfa1 cells).^13^, ^95^, ^100^ Another isoenzyme, ACSL1, has recently been described to mediate ferroptosis by conjugated linoleic acids.^101^ Monounsaturated fatty acids (MUFAs) lack the bis‐allylic double bond of PUFAs and protect from membrane peroxidation. The biosynthesis of MUFA‐containing phospholipids via stearoyl‐CoA desaturase 1^96^, ^102^ and ACSL3^2^ accordingly exerts antiferroptotic effects and contributes to stress adaption, partially through the lipokine PI(18:1/18:1).^103^, ^104^, ^105^

### 2.6 Other pathways

Ferroptosis is closely linked to (i) cell–cell contact‐mediated signal transduction, for example, via the E‐cadherin–NF2–Hippo–YAP/TAZ axis,^55^, ^106^ (ii) cell metabolism,^5^, ^11^ among others via the energy sensor AMP‐activated kinase (AMPK)^13^, ^96^ and glutamate/glutamine metabolism,^107^ and (iii) the production of reactive oxygen species (ROS). Major sources for (lipid) ROS are nicotinamide adenine dinucleotide phosphate (NADPH) oxidases (NOXs), LOX isoenzymes, cytochrome P450 oxidoreductases, and electron leakage from the mitochondrial electron transport chain.^36^, ^88^, ^108^, ^109^ Ferroptosis is also modulated by epigenetic mechanisms. For example, the histone‐lysine *N*‐methyltransferase 2B (MLL4) promotes ferroptosis by rewiring the expression of ferroptosis‐related gene sets,^110^ whereas the ubiquitin carboxyl‐terminal hydrolase BRCA1‐associated protein 1 supports ferroptosis by repressing SLC7A11 besides participating in multiple cellular processes related to DNA damage repair, cell cycle control, and the immune response.^111^ These pleiotropic enzymes, pathways, and regulatory factors are not the focus of this article, either because few is known about how their interaction with small molecules influences ferroptosis or because they are covered by recent review articles.^1^, ^5^, ^9^, ^11^, ^112^

There are also many links between ferroptosis and immunoregulation existing. Recent reports suggest that ferroptosis is involved in the physiological processes of the immune system to suppress tumor growth.^2^, ^113^ Activated CD8^+^ cells, for example, induce tumor cell ferroptosis by secreting interferon γ (IFNγ), which subsequently downregulates SLC7A11 and upregulates ACSL4 in cancer cells.^41^ Moreover, the oxidized phospholipid, 1‐steaoryl‐2‐15‐HpETE‐*sn*‐glycero‐3‐phosphatidylethanolamine was identified as a key “eat me” signal for the phagocytotic clearance of cancer cells in vivo.^114^

## 3 FERROPTOSIS, CANCER, AND CHEMORESISTANCE

### 3.1 Ferroptosis and cancer

Mounting evidence suggests the induction of ferroptosis as a promising strategy for the treatment of various types of cancer, including therapy‐resistant forms.^1^, ^115^ Cancer cells are more susceptible to ferroptosis than nonmalignant cells for several reasons: they (i) have persistently high ROS levels as a consequence of genetic alterations and aberrant proliferation,^116^ (ii) have elevated intracellular iron levels,^88^, ^117^, ^118^ and (iii) rely on oncogenic signal transduction that is crosslinked to ferroptotic pathways.^1^ Given the strong differences in ferroptosis sensitivity between cell types, it is obvious that the efficacy of antiferroptotic therapy varies between tumors from different origins and genotypes. The applicability of ferroptosis‐inducing drugs to treat individual tumors, therefore, requires careful evaluation in terms of personalized medicine.

Cancer cells upregulate endogenous antioxidative systems, for example, GPXs, superoxide dismutases (SODs), GSH, catalase, peroxiredoxins (PRDXs), and thioredoxins, to maintain redox balance and ensure cell survival despite elevated ROS production.^70^ By keeping oxidative stress below a certain threshold, they avoid irreparable cell damage leading to the induction of PD as a consequence of lipid, protein, or DNA (per)oxidation.^119^ Note that cell death by oxidative stress is neither unique to ferroptosis nor cancer cells. ROS induction or the inhibition of antioxidative enzymes evokes various forms of cell death like apoptosis, necrosis, and autophagy and is deleterious to cancer and normal cells. However, the increased ROS levels of cancer cells likely confer to their higher vulnerability toward pro‐oxidants as compared to nontransformed cells.^120^, ^121^, ^122^ In addition, the elevated iron levels in tumors as compared to healthy tissues^123^, ^124^, ^125^ may contribute to cancer‐specific ROS formation and lipid peroxidation via the Fenton reaction.^126^ It is tempting to speculate that cells with high basal ROS levels are more sensitive to ferroptosis and that the targeted induction of ferroptotic pathways (that channel ROS damage to membranes) adds to this selectivity.^127^, ^128^, ^129^ Interestingly, endogenous ROS concentrations are even further elevated in cancer cells resistant to chemotherapy or radiotherapy, which might render them more sensitive to ROS‐driven ferroptosis.^127^, ^128^

### 3.2 Ferroptosis and tumor resistance

Drug resistance mechanisms of cancer cells impact ferroptosis sensitivity.^1^ Most relevant in this context are: (i) the adaption to the microenvironment and activated defense systems,^130^ (ii) mutated drug target genes or oncogenes that confer protection,^131^ (iii) as well as mechanisms that regulate the cellular differentiation status, including stemness or epithelial‐to‐mesenchymal transition (EMT).^58^ Another major strategy of cancer cells to acquire drug resistance is the expression of transport proteins, for example, multidrug resistance proteins like P‐glycoprotein, that pump chemotherapeutics out of the cells. In some cases, therapy‐resistant cancer cells are even more sensitive to ferroptosis than less aggressive malignant cells.^87^ The exact mechanisms that render therapy‐resistant cancer cells sensitive to ferroptosis are incompletely understood. Many ferroptosis‐related factors (e.g., NRF2, SLC7A11) are upregulated by tumor resistance mechanisms,^70^ among which EMT seems to play a prominent role.^87^ In consequence, aggressive cancer cells that are resistant to other forms of PD (e.g., apoptosis) often remain vulnerable to ferroptosis. While it is out of the scope of this review to outline all the complex interconnections between ferroptosis, cancer and drug resistance, we want to point out three promising drug targets at this interface, namely NRF2, SLC7A11, and GPX4, and refer to comprehensive review articles in this field.^21^, ^42^, ^48^, ^70^, ^132^ For all three proteins, a growing number of ligands and indirect modulators has been reported during recent years, which are described in Sections 5, 6, and 8.

## 4 INDUCING FERROPTOSIS BY SMALL MOLECULES

Since the term ferroptosis has been coined in 2012, the field prospered and important discoveries on the complex ferroptosis machinery have been made. With some delay and following a better understanding of the relevance of ferroptosis in cancer and drug resistance, research on ferroptosis‐inducing small molecules has been intensified, resulting in an exponentially growing number of published articles on this field. Ferroptosis‐inducing small molecules promise novel therapeutic options for the treatment of (therapy‐resistant) cancer.^133^ They interact with transport proteins (e.g., system X_c_^−^), enzymes (e.g., GPX4), or transcription factors (e.g., NRF2) or nonenzymatically initiate ROS formation via the Fenton reaction. Rational drug development against these targets is hampered by nonclassical binding modes and insufficient structural information about putative binding pockets. For example, there are several electrophilic inhibitors of GPX4 described that covalently react with the active site selenocysteine,^69^ but these compounds lack selectivity and have poor pharmacodynamic properties.^4^ While these inhibitors establish stable covalent bonds with the enzyme, the initial positioning of the ligand is kinetically disfavored without pronounced noncovalent protein–ligand interactions. The majority of approved drugs target well‐defined binding sites, either competitively at substrate or ligand binding pockets or substrate independently at allosteric sites. This classical mode of action of noncovalent inhibitors is extended by the warhead concept that aims at covalently coupling the ligand to cysteine residues (or other reactive groups) close to the binding site.^134^ Combining specific noncovalent interactions with an irreversible modification promises superior potency as well as selectivity. Ferroptosis‐inducing small molecules with such features are of high pharmacological interest and represent potential chemical tools for investigating redox‐regulated processes in cell signaling.^135^ Along these lines, oxidative protein modifications have recently been suggested as another level in the regulation of signaling cascades in addition to phosphorylation.^136^ In support of this hypothesis, the reactivity of cysteine residues within the cysteinome varies by seven magnitudes of order.^136^ Little is known so far how redox‐dependent cysteine modifications achieve target selectively under physiological conditions, which renders the design of selective inhibitors or activators challenging.

With the erastin analogue PRLX 93936 (**21**), a selective inhibitor of system X_c_^−^ entered clinical trials already in 2007 (www.clinicaltrials.gov, NCT00528047), with another study following in 2012 (NCT01695590). More recent clinical studies (based on single or combinatory treatments) focused on dimethyl fumarate (three clinical trials for cancer treatment, phase I–III, 2015–2016, NCT02784834, NCT02546440, and NCT02337426) and the natural products artemisinin/artesunate (eight clinical trials for cancer, phase I–II, 2008‐2021, NCT02633098, NCT03093129, NCT00764036, NCT02353026, NCT02354534, NCT03792516, NCT03100045, NCT04098744, NCT02786589, NCT05478239, NCT02304289), phenylethyl isothiocyanate (PEITC, **71**) (seven clinical trials, phase I–III, 2004‐2021, NCT00968461, NCT00005883, NCT00691132, NCT01790204, NCT03700983, NCT03034603, and NCT02468882), and β‐elemene (**56**) (two clinical trials, phase II–III, 2012‐2021, NCT02629757, NCT03123484), and not further specified elemenes (six clinical trials, phase I–IV, NCT04674527, NCT04401059, NCT03166553, NCT03167775, NCT04397432, NCT01679847). Major breakthroughs were, however, hampered by the poor druggability of key players in ferroptosis, that is, GPX4 and NRF2, both of which lack a classical binding pocket for “drug‐like” small molecules that dominate high‐throughput screening. Secondary metabolites from plants and other organisms cover a broader chemical space and include complex structures with multiple stereogenic centers, diversified polycyclic ring systems, and substitution patterns that might overcome these limitations.^3^, ^137^ We consider natural products as a rich source of novel lead structures for targets in ferroptosis, which are otherwise difficult to address, like GPX4 and NRF2, or where the target is either unknown or not associated with ferroptosis. The number of small molecules that induce ferroptosis, trigger membrane peroxidation, or target NRF2 signaling is rapidly increasing, as is the preclinical evidence for their efficacy in suppressing drug‐resistant cancer.^47^, ^48^, ^138^ There is a high demand for small molecules that induce ferroptosis, whether for use as chemical probes to unravel ferroptosis‐associated pathways or as drug candidates to fight aggressive cancer.^135^

## 5 INHIBITORS OF SYSTEM X_C_^−^

Before being recognized as a major target in ferroptosis, system X_c_^−^ was already known to support tumor growth, survival, and drug resistance and to contribute to neurological dysfunction.^139^, ^140^, ^141^, ^142^, ^143^ Efforts have been undertaken to identify small molecule system X_c_^−^ inhibitors for therapeutic use.^54^ High‐throughput screening led to the identification of the potent system X_c_^−^ inhibitor erastin (**18**) and disclosed additional modes of action for the approved multikinase inhibitor and anticancer drug sorafenib (**22**), which shows moderate system X_c_^−^‐inhibitory activity.^54^

### 5.1 Quisqualic acid (**1**) and isoxazole derivatives

**Figure 2:**
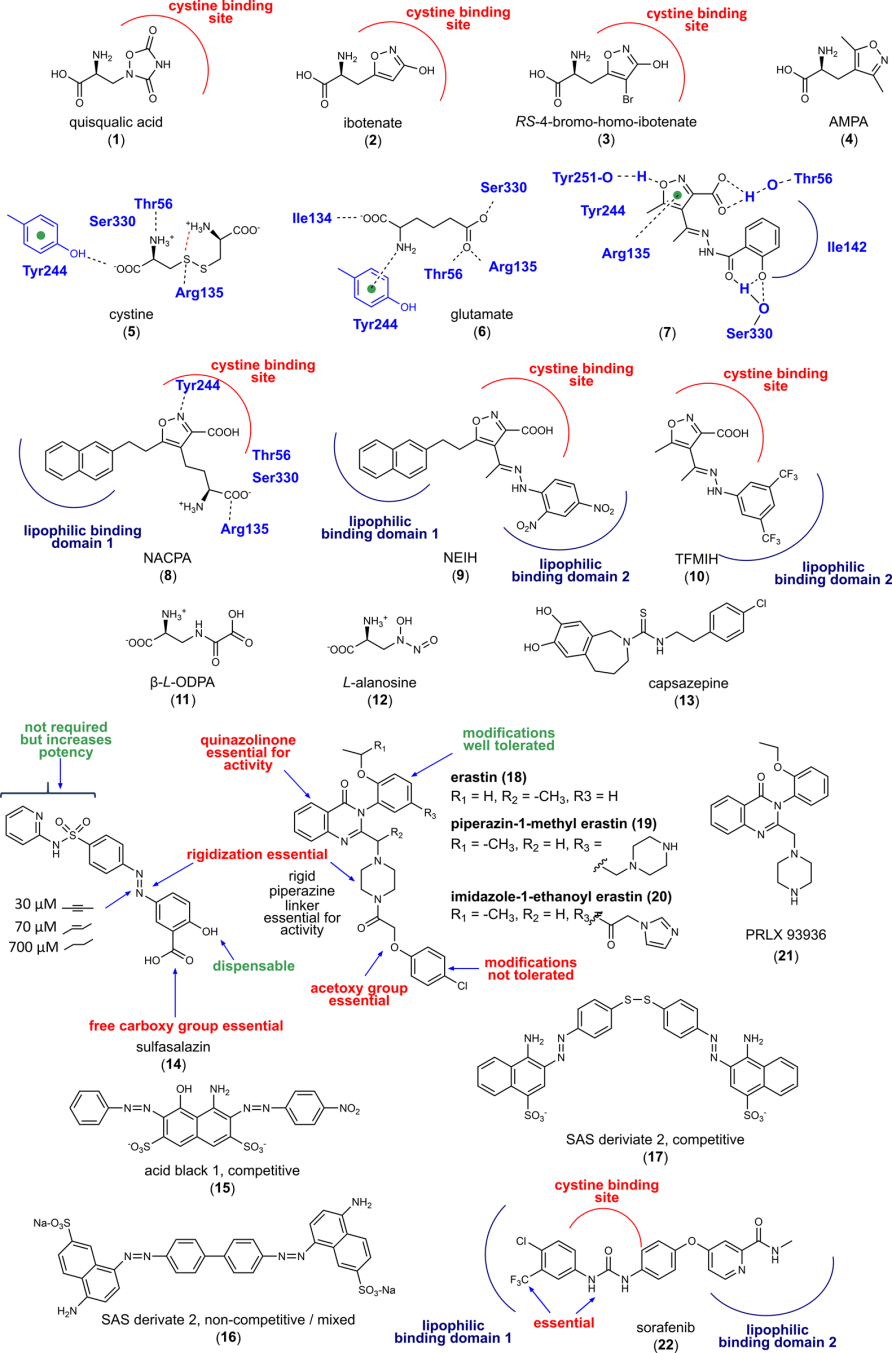
Inhibitors of system X_c_^−^. [Color figure can be viewed at wileyonlinelibrary.com]

Compound **1** from *Quisqualis indica* and ibotenate (**2**) from *Amanita muscaria* and *Amanita pantherine* are analogues of α‐amino‐3‐hydroxy‐5‐methyl‐4‐isoxazole‐4‐propionic acid (AMPA, **4**) (Figure 2), an endogenous activator of ionotropic glutamate receptors (AMPA receptors).^144^ They are substrates of system X_c_^−^ and inhibit the antiporter (*K*_i_ = 5 µM).^144^ System X_c_^−^ transports ibotenate with comparable efficiency to the endogenous substrate cystine but is less efficient for bromohomoibotenate (**3**) and **1**. Nuclear magnetic resonance analysis suggests that the endogenous substrate cystine (**5**) adopts a ring‐like conformation that is stabilized by an intracellular hydrogen bond between sulfur and the amino group, an orientation that aligns with the pentacyclic system of **1** and **2** (Figure 2).^144^

According to docking studies on an X_c_^−^ homology model, the α‐amino acid head group of **5** and glutamate (**6**) interact with Tyr244 of SLC7A11, whereas the distal carboxy groups bind to Thr56, Arg135, and Ser330, with Arg135 being located near to the X_c_^−^ glutamate binding site (Figure 2).^145^ Within a series of isoxazole derivatives synthesized by the authors, compound **7** was most active and inhibited glutamate uptake by 50% at 500 µM.^145^ Computational calculations indicate four principal interactions that govern the binding of compound **7** to SLC7A11: (i) a hydrogen bond between isoxazole‐3‐carboxylate and Thr56, (ii) π–π interaction of the isoxazole ring with Arg135, (iii) lipophilic interactions with Ile142, Tyr244, and Il134, and (iv) a hydrogen bond between the phenolic hydroxy group and Ser133.

Structural optimization of the conformationally restrained isoxazole scaffold yielded derivatives with bulky lipophilic residues in 3‐ and/or 4‐position (NACPA (**8**), NEIH (**9**), and TFMIH (**10**); *K*_i_ = 3–100 µM) (Figure 2),^146^ which efficiently inhibit system X_c_^−^ but are no longer substrates. Interestingly, the substitution pattern determines whether system X_c_^−^ inhibition is competitive (one lipophilic substituent) or noncompetitive (two lipophilic substituents). The authors propose that the isoxazoles target two lipophilic pockets adjacent to the substrate binding site. Given the structural similarities to glutamate, it is not surprising that **1** and derivatives show many cross‐reactivities, in particular with glutamate receptors of the central nervous system.^147^

### 5.2 β‐*N*‐oxalyl‐l‐α‐β‐diaminopropionic acid (**11**)

The excitotoxin **11** (Figure 2) from *Lathyrus species* (e.g., *L. sativus, L. clymenum*, and *L. cicera*) activates non‐N‐methyl‐d‐aspartate receptors (*K*_D_ = 1.3 µM).^148^, ^149^ Later, compound **9** was identified as a competitive inhibitor and alternative substrate of system X_c_^−^ in rat LRM55 glia cells and human SNB‐19 glioma cells (80%‐85% inhibition of glutamate transport at 500 µM), and it has been speculated that an intracellular accumulation of **11** enlarges the target profile to thus far unknown proteins.^149^

### 5.3 l‐Alanosine (**12**)

The amino acid antimetabolite **12** (Figure 2) from *Streptomyces alanosinicus* carries a 3‐hydroxynitrosamine group in the side chain and has pronounced antitumoral activity in vitro and in vivo.^150^ Correlations between SLC7A11 gene expression and the potency of 1400 anticancer agents over 60 human cancer cell lines revealed **12** as inhibitor and substrate of system X_c_^−^ with half maximal inhibitory concentration (IC_50_) values ranging from 0.86 to 53 µM in different carcinoma cell lines.

### 5.4 Capsazepine (**13**)

The thiourea derivative **13** (Figure 2) derives from capsaicin, the active ingredient of chili pepper, and competitively inhibits vanilloid receptor‐1 (TRPV‐1), though with moderate potency (IC_50_ = 0.42 µM).^151^ Compound **13** reversibly binds to system X_c_^−^ (EC_50_ = 3 µM), elevates ROS levels in human MDA‐MB‐231 breast cancer cells (at 25 µM), and triggers cell death at high concentrations (10% at 25 µM **13**; 90% at 100 µM **13**).^152^

### 5.5 Sulfasalazin (**14**)

The Food and Drug Administration (FDA)‐approved anti‐inflammatory drug **14** (brand name Azulfidine, Salazopyrin, Sulazine, etc.) was identified in 1985 as a moderate inhibitor of system X_c_^−^ (IC_50_ = 30 µM) (Figure 2).^153^ Several clinical studies with **14** for the (supportive) treatment of breast cancer and brain tumors are ongoing (www.clinicaltrials.gov, NCT03847311, NCT01577966, NCT04205357). While the diazo group of **14** has to be cleaved to yield the anti‐inflammatory active compound mesalazine, this cleavage is detrimental to system X_c_^−^ inhibition.^154^ The poor metabolic stability of the diazo group thus limits the application of **14** as anticancer drug.^153^ structure–activity relationship (SAR) studies revealed that the replacement of the diazo group by a linear alkyne (Figure 2) is tolerated without loss of activity (IC_50_ = 30 µM) and improves metabolic stability. Replacement by ethenediyl (Figure 2) is accepted with slightly decreased inhibitory potency (IC_50_ = 70 µM), whereas the saturated ethane bridge (Figure 2) abolishes system X_c_^−^ inhibition (IC_50_ = 700 µM), which indicates that rigidization is essential for activity. The free carboxylic acid of **14** is also important for target interaction; methyl esters are not active. Removal of the phenolic alcohol group is tolerated and potentially enhances metabolic stability by eliminating phase II metabolism. Together, the structural optimization of **14** yielded derivatives with improved metabolic stability, but their affinity to system X_c_^−^ remained low. Accordingly, compound **14** less potently inhibits cystine uptake in human HT‐1080 fibrosarcoma and human Calu‐1 non‐small‐cell lung cancer (NSCLC) cells (EC_50_ = 32 µM) than erastin (EC_50_ = 0.3 µM).^54^ We conclude that **14** induces ferroptosis in various cancer cell lines but has major limitations as a drug (template) for ferroptosis‐based anticancer therapy.

Supposedly inspired by the isoxazole derivatives **8, 9**, and **10**, analogues of **14** with lipophilic moieties (**15–17**) were designed that might address the same lipophilic binding pockets as proposed for the substituted isoxazoles (Figure 2).^155^ With increasing length and lipophilicity of the substituents, the binding mode switches from competitive to noncompetitive, similar to what was observed for aryl‐substituted isoxazoles, which further strengthens the hypothesis that allosteric sites are located close to the substrate pocket. The thus optimized derivatives of **14** gained inhibitory potency (2.5‐fold) but are of limited therapeutic value for the treatment of glioblastoma because they cannot cross the blood‐brain barrier.^155^

### 5.6 Erastin **(18)** and analogues **19** and **20**

By screening small molecules that activate iron‐dependent cell death in oncogenic‐RAS‐harboring cancer cells, **18** (Figure 2) was identified as system X_c_^−^ inhibitor with the novel scaffold.^88^, ^118^ Compound **18** (10 µM) induces ferroptosis in N‐RAS‐mutant HT‐1080 fibrosarcoma cells by inhibiting system X_c_^−^ and lowering the cellular levels of GSH,^156^, ^157^ which is needed by GPX4 as cofactor to reduce lipid hydroperoxides. With an IC_50_ value of 1.4 µM, compound **18** inhibits cystine uptake in Slc7a11‐overexpressing MEFs more potent than other systems X_c_^−^ inhibitors (IC_50_ = 26.1 µM for **14**, 4.4 µM for 2‐(*S*)‐(4‐carboxyphenyl)glycine [CPG] and 15.4 µM for **1**).^158^ The potency of ferroptosis inducers, including **18**, strongly differs between cell lines and experimental settings. For example, human BJ‐TERT/LT/ST/RAS^G12V^ tumorigenic fibroblasts are considerably more sensitive to **18** (EC_50_ = 1.25 µM) than primary BJ fibroblasts (EC_50_ > 5 µM, selectivity > 8).^159^ Modifications of **18** are only tolerated at the ethoxy‐phenyl moiety, with a biphenyl substituent increasing the potency 10‐fold (Figure 2). Modifications at other sites, in particular at the rigid piperazine linker, reduced or abrogated system X_c_^−^ inhibition (40% inhibition of glutamate release at 10 µM in human HT‐1080 fibrosarcoma cells).^54^, ^160^ Note that a rigid linear structural element is also present in derivatives of **14** and in the isoxazoles **8, 9**, and **10** (where isoxazole was suggested to mimic cystine) and seemingly represents a common feature of potent system X_c_^−^ inhibitors. It is tempting to speculate that these rigidized inhibitors occupy the same binding site.

**Figure 3:**
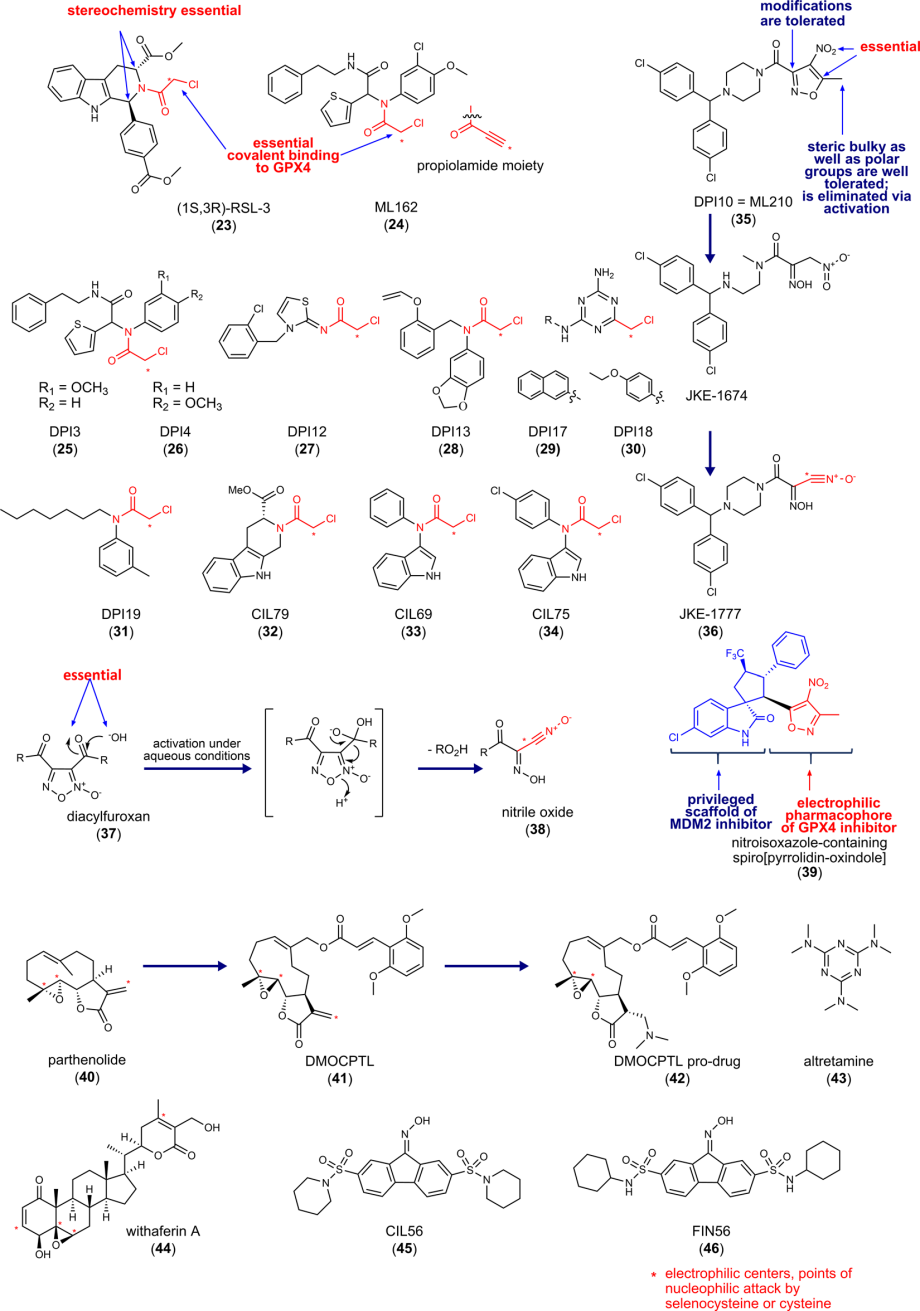
Inhibitors of glutathione peroxidase 4. MDM2, mouse double minute 2. [Color figure can be viewed at wileyonlinelibrary.com]

Compound **18** irreversibly inhibits system X_c_^−^,^158^ which is unique among system X_c_^−^ inhibitors and not shared by **14**, CPG, and **1**.^158^ Thus, **18** is a serendipitously discovered targeted covalent inhibitor (TCI).^134^ These inhibitors have a bond‐forming group with relatively low reactivity and additional residues that noncovalently bind the target.^134^ The additional residues are required for the exact positioning of the inhibitor, which, after binding, reacts rapidly with the noncatalytic residue, in most cases a cysteine. The acetoxy group is the most likely point of attack for reactive cysteines in **18**, which might explain why this moiety is indispensable for inhibitory activity. Of note, the acetoxy linker resembles the chloroacetamide group of GPX4 inhibitors (Figure 3), which covalently bind to selenocysteine and cysteine. Where **18** exactly binds system X_c_^−^ is not known. Mutation of six endogenous cysteine residues (Cys86, Cys158, Cys197, Cys271, C327, and Cys435) in SLC7A11 did not result in a loss of inhibitory activity, which suggests that **18** either reacts with alternative sulfhydryl groups or binds to amino acid residues that were not investigated.^158^ Further studies are required to elucidate the binding mode of **18**.

The poor water solubility and metabolic stability of **18** limit its application in vivo. Introduction of piperazin‐1‐methyl (**19)** or imidazole‐1‐ethanoyl (imidazole ketone erastin **20)** at 5‐position of the ethoxy‐phenyl ring yields derivatives of **18** with 16‐fold (**19**) and threefold (**20**) improved water solubility (Figure 2).^54^ In addition, system X_c_^−^ inhibitory activity was strongly enhanced for **20** as compared to **18** in human BJeLR fibroblasts (**20**: IC_50_ = 3 nM; **18**: IC_50_ = 625 nM).^10^ When given intraperitoneally (ip) at 23 or 40 mg/kg, **20** reduced tumor size in a human B cell lymphoma SUDHL6 cell xenograft model in mice.^161^ Referring to the pharmacokinetic profile, the authors stated that **20** might not be suited to counter rapid tumor growth in vivo. The analogue of **18**, PRLX 93936 (**21**), was subjected to two clinical trials investigating safety, efficiency, and pharmacokinetics in patients with advanced solid tumors and multiple myeloma in 2007 and 2012 (www.clinicaltrials.gov, NCT00528047 and NCT01695590). The outcome has not been published. Notably, compound **21** lacks the 4‐chloro‐phenoxyethanoyl moiety, which is considered to be essential for system X_c_^−^ inhibition by **18**.

### 5.7 Sorafenib (**22**) and analogues

Compound **22** is a multikinase inhibitor with anticancer properties that inhibits system X_c_^−^ as subordinate target (Figure 2).^54^ SARs studies with 87 analogues of **22** indicate that similar features are required for system X_c_^−^ inhibition than for binding of **22** to kinases like B‐RAF. Accordingly, the ferroptosis‐inducing activity was diminished by removing the lipophilic *meta*‐CF_3_ group that points toward the hydrophobic pocket of the kinase or by modifying the urea structure that forms two hydrogen bonds between the urea substructure and Glu^501^ of B‐RAF.^162^ These findings allow for two interpretations. Either **22** binds to a pocket of system X_c_^−^, which resembles the interaction site at the kinase, or inhibits a yet unknown kinase that lowers X_c_^−^ antiporter activity.^54^ Of note, the urea structure is also an integral part of the 1‐oxa‐2,4‐diazolidine‐3,5‐dione ring of **1** that was replaced by isoxazole in **8, 9**, and **10**. These scaffolds mimic the preferred conformation of cystine and were proposed to target the system X_c_^−^ substrate binding site and additionally extend to lipophilic allosteric regions.^147^ We speculate that **22**, which consists of a urea core with two lipophilic substituents, shares this binding (Figure 2). Recently, analogues of **22** were described that trigger multiple forms of cell death, including ferroptosis, apoptosis, and autophagy, depending on compound concentration and incubation time.^163^

Novel synthetic small molecule inhibitors of system X_c_^−^ with cytotoxic effects on various cancer cell lines (lung, cervix, muscle, colon, breast, and prostate; EC_50_ = 5–10 µM) were identified in 2019. Strongest effects were observed for cells with the mesenchymal phenotype (e.g., human HT1080 fibrosarcoma cells and human Rh30 and Rh41 rhabdomyosarcoma cells), whereas epithelial cells (MCF7, HCT116) were only slightly affected.^164^

### 5.8 Other inhibitors of system X_c_^−^

Cystine uptake via system X_c_^−^ can be competitively blocked by compounds that mimic substrates of the antiporter, such as *l*‐glutamate, α‐aminoadipate, *l*‐homocysteate, *l*‐serine‐O‐sulfate, and *l*‐cystathionine^165^ as well as CPG, 2‐(*S*)‐(4‐sulfophenyl)glycine, 2‐(*S*)‐[4‐(4‐carboxyphenyl)phenyl]glycine, and 2‐*(S*)‐(5‐sulfothien‐2‐yl)glycine.^144^, ^158^ Moreover, mercurial reagents, such as *p*‐chloromercuribenzoic acid and *p*‐chloromercuribenzenesulfonic acid potently inhibit system X_c_^−^ activity by interacting with Cys327 of SLC7A11.^158^

## 6 GPX4 INHIBITORS

Eight glutathione peroxidase (GPX) isoenzymes exist in humans; five of them are selenoproteins with selenocysteine in the active center.^166^ Selenocysteines are markedly more nucleophilic than cysteines,^167^ which adds to their enzymatic reactivity and renders them prone to electrophilic attacks. GPX4 is unique among GPX isoenzymes for its ability to accept complex phospholipid hydroperoxides as substrates,^168^ which can be explained by the monomeric rather than tetrameric structure and the lack of a surface‐exposed loop lining the active site that is present in other GPX isoenzymes.^169^ The flat binding cleft of GPX4 consists of long, apolar residues that surround the catalytic tetrad (Sec46, Gln81, Trp136, and Asn137) and extends into a positively charged surface consisting of basic amino acids (K48, K135, and R152).^169^, ^170^ With regard to the substrates of GPX4, the apolar region (Met156, Pro155, Gly154, I1e29, Leu130, Ala133, I1e34, Phe78, and Gly79) likely supports the binding of lipid hydroperoxides, whereas the positively charged area might interact with the negatively charged phosphate in phospholipids. Interestingly, GPX4 has a structural motif that consists of Cys148, Gln123, and Trp119 and resembles the catalytic tetrad but is located on the opposite side of the enzyme.^169^ Site‐directed mutagenesis studies suggest that this motif does not contribute to catalysis, though it might be involved in binding ligands that allosterically modulate GPX4 activity. In fact, two synthetic activators of GPX4 have recently been found to bind to this putative allosteric site.^171^, ^172^

### 6.1 Direct inhibitors of GPX4

#### 6.1.1 Chloroacetamide derivatives

RSL3 (**23**) (Figure 3) was identified in tumorous fibroblast‐derived cell lines as a small molecule with oncogenic RAS‐selective lethality (RSL) in 2008, and GPX4 was identified as a target in 2014.^69^, ^118^ In contrast to **18**‐like compounds, which lower GPX4 activity by depleting the cosubstrate GSH, (1*S*,3*R*)‐RSL3 (**23**) directly inhibits GPX4 by covalently binding to Sec46 in the active center via the chloroacetamide motif.^69^ (1*S*,3*R*)‐RSL3 (50 nM) suppresses GPX4 activity in human COH‐BR1 breast cancer cells that overexpress GPX4 (L7G4 variant). The two stereogenic centers of **23** are important for inhibitory activity. While the (1*S*,3*R*)‐stereoisomer potently inhibits GPX4, the (1*R*,3*S*)‐enantiomer, and the (1*S*,3*S*)‐ and (1*R*,3*R*)‐diastereomers are more than 100‐fold less active. Moreover, they do not show a preference for killing human BJ‐derived fibroblasts with an oncogenic RAS mutation (H‐RAS^G12V^) over BJ‐derived fibroblasts with wild‐type H‐RAS.

In a large‐scale screen with over 1,000,000 compounds, 14 ferroptosis‐inducing compounds were identified with similar chemical structure, 7 of them have an electrophilic chloroacetamide moiety and inhibit GPX4 (Figure 3; **24–31**).^69^ The compounds selectively suppress cell growth in human H‐RAS^G12V^‐expressing BJ fibroblasts with GI_50_ values ranging from 0.02 to 0.3 µM (compare with **23**: GI_50_ = 0.15 µM).

The hit compound ML162 (**24**) was subjected to SARs studies, which focused on the substitution pattern at the thiophene, chloromethoxy, or phenethylamine moiety but did not lead to more potent or selective analogs. ML162 surpassed **23** in inducing cytotoxicity (0.025 vs. 0.1 µM, respectively), although both compounds were comparably efficient in inhibiting the GPX4‐dependent degradation of phosphatidylcholine hydroperoxides in the cellular context.^69^, ^173^

Another screen for cytotoxic agents with 3169 compounds identified 10 compounds that induce ferroptosis. Three of them (**32–34)** possess again an electrophilic chloroacetamide moiety and closely resemble the structure of **23**, with compound **32** only lacking the 4‐(methoxycarbonyl)phenyl moiety and consequently one of the stereogenic centers.^174^

Nonfavorable pharmacodynamic properties as well as high reactivity to off‐target proteins are major disadvantages of strong electrophiles like chloroacetamides.^4^ Weak electrophiles like acrylamides or fumarates might improve target selectivity but are not compatible with covalent GPX4 inhibition, despite the high nucleophilicity of selenocysteine,^4^ likely due to slow reaction kinetics. Increasing the enzyme resident time by exploiting noncovalent interactions might circumvent these limitations.

The 4‐nitro‐1,2‐oxazol derivative *DPI10* (also named ML210, **35**), identified in the above‐mentioned large‐scale screen,^4^ inhibits GPX4 in a cellular system despite lacking an electrophilic chloroacetamide moiety but fails to reduce GPX4 activity under cell‐free conditions. Subsequent SARs studies guided the structural optimization toward compound **35** which selectively induces ferroptosis in H‐RAS^G12V^ BJ cells.^173^ Compound **35** is a masked electrophile with a nitroisoxazole system that is converted to the metabolite JKE‐1674 and subsequently to the reactive nitrile oxide (**36**) (Figure 3), which forms a covalent adduct with GPX4.^4^ Masking the reactive electrophile as prodrug improves the selectivity of **35** as compared to **23** and **24**, which both show high off‐target activity in a spectrometry‐based proteomics screen.^4^ Notably, the proteins interacting with **35** differ from those **23** and **24** binds to. Compound **34** mainly interacts with abundant proteins, for example, tubulin, whereas **23** and **24** both have a more defined off‐target profile. Masking the reactive group in **35** also makes the inhibitor more stable, however at the expense of inhibitory activity.^4^ Replacement of chloroacetamide in **23, 24, 28**, and **31**, by nitroisoxazole resulted in inactive compounds except for **31**.^175^ This finding indicates that concrete structural elements apparently underlie the intracellular enzymatic activation of the heterocyclic nitro group as nitril oxide. Eaton et al.^176^ described further nitrile oxide prodrugs with a diacylfuroxan scaffold (**37**) (Figure 3), but the proteome‐wide reactivity of these compounds rather excludes the masking of nitrile oxide as diacylfluroxan is an effective mean to enhance the selectivity for GPX4. The prodrugs seem inherently unstable and are subjected to multiple decomposition pathways. Together, masked electrophiles are valuable probes to study GPX4 function but have not been developed into promising drug candidates thus far.^176^

Compound **24**, the most potent GPX4 inhibitor identified to date, was used as a template in a systematic study to search for possible replacements for the chloroacetamide warhead.^175^ Among the 25 electrophilic groups tested, only propiolamides retained the ability to induce ferroptotic cell death. In derivatives of **35**, chloroacetamide, propiolamide, and trimethylsilyl propiolamide warheads are tolerated as substitutes for the original 5‐methyl‐4‐nitro‐3‐isoxazolyl moiety. Propiolamides, which are highly reactive and effectively bind thiol nucleophiles, were expected to face similar challenges in selectivity, pharmacokinetics, and stability to chloroacetamides. It was therefore surprising that cotreatment with the ferroptosis inhibitor ferrostatin‐1 raised the IC_50_ value of the propiolamide derivative to **24** stronger than for the lead compound, which might hint toward a superior GPX4 selectivity.^175^

**Figure 4:**
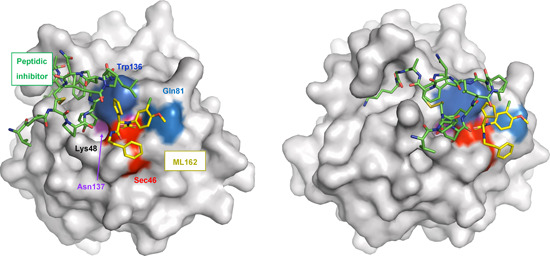
Human GPX4 (C66S) in complex with ML162 (*S* enantiomer). The surface of GPX4 with bound (*S*)‐ML162 (depicted with yellow carbons) (PDB entry 6HKQ) was superimposed to a structure that shows the peptide inhibitor GXpep3 (depicted with green carbons) bound to GPX4 (U46C) (PDB entry 5h5s). The surface of the catalytic tetrad is colored red (Sec46), blue (Gln81), dark blue (Trp136), and purple (Asn137). Two different orientations are shown. Reproduced with permission of the International Union of Crystallography.^177^ GPX4, glutathione peroxidase 4; PDB, Protein Data Bank. [Color figure can be viewed at wileyonlinelibrary.com]

Because of the challenging bacterial expression of selenocysteine‐containing proteins, most crystal structures available for GPX4 used the GPX4^U46C^ mutant that lacks the catalytic selenocysteine. Moosmayer et al.^177^ recently disclosed the crystal structure of wild‐type human GPX4 in complex with the covalent inhibitor **24** (Figure 4). Compound **24** does interestingly not only covalently bind to Sec46 but also to Cys66, which underlines the limited selectivity of chloroacetamide warheads. This finding implies that **24** likely reacts with a broad spectrum of proteins within the cysteinome. Further studies were conducted on the GPX4^C66S^ mutant, in which the reactive cysteine Cys66 was replaced by serine to prevent heterogeneous covalent modifications.^177^ Compound **24** is located at the shallow surface pit near the catalytic tetrad, where it interacts with Gln81, Trp136, and Asn137 of the catalytic tetrad. Note that GPX4 inhibitory peptide GPX4 binding peptides (GXpep)‐3 described later in this section occupies a different surface area of GPX4 (Figure 4).^177^ The cocrystal structure of GPX4 with **24** confirmed the lack of a well‐defined deeper binding pocket but revealed a subpocket adjacent to Sec46, which is located between Lys48 and Trp136 and might be occupied by inhibitors to enhance selectivity.^177^

An elegant cell‐based study on the binding mode of diverse direct and indirect GPX4 inhibitors recently addressed the impact of the most reactive cysteine residues in GPX4 (Cys2, Cys10, Cys37, Sec46, Cys66, Cys75, Cys75, Cys107, and Cys148) on enzyme activity.^178^ Interestingly, it was found that GPX4 inhibition does not exclusively rely on the covalent coupling to active site Sec46 but also involves covalent modification of Cys66 (which is close to the active site) and Cys10.^178^ For example, the direct GPX4 inhibitors RSL3 and ML162 selectively target Sec46 and Cys66 and, in case of ML162, additionally bind to the less reactive GPX4^Sec46Cys^ mutant, for which the active center selenocysteine was replaced by a less reactive cysteine. Covalent binding to Cys66 induces conformational changes and subjects GPX4 to degradation. In addition, Cys10, as well as Cys66, are important for the regulation of GPX4 activity under limited GSH supply, when cells were treated with the system X_c_^−^ inhibitor **20**. It was suggested that GPX4 forms a locked inactive dimeric form of GPX4 under limited GSH conditions via Cys10 (GPX4–Cys10–Sec46–GPX4) that is regenerated into active GPX4 by reductive cleavage involving Cys66 from a third GPX4 protein. Other indirect GPX4 inhibitors, that is, FIN56 and FINO2, do not bind to Cys66. In summary, a complex regulatory system exists in cells that fine‐tunes the activity of GPX4 under distinct cellular conditions.

#### 6.1.2 Nitroisoxazole derivatives

Novel nitroisoxazole‐containing spiro[pyrrolidine‐oxindoles] (**39**) were designed as dual inhibitors of GPX4 and mouse double minute 2 (MDM2) (Figure 3) to induce both ferroptosis and apoptosis in cancer cells. Cellular thermal shift assays indicate that these compounds covalently bind to GPX4. The dual inhibitors exhibit inhibitory effects on MDM2 and efficiently reduced the tumor volume in a murine MCF‐7 xenograft model at 25 mg/kg (ip).^179^

#### 6.1.3 GPX4 inhibitory peptide GXpep3 (Val–Pro–Cys–Pro–Tyr–Leu–Pro–Leu–Trp–Asn–Cys–Ala–Gly–Lys)

The crystal structure of GPX4 in complex with the GXpep 1–3 has been resolved for a mutant enzyme (Cys37Ala, Cys93Arg, Cys134Glu, Cys175Val, Cys64Ser, Cys102Ser, and Sec73Cys, and an N‐terminal SUMO‐tag) and revealed noncovalent interaction.^180^ GXpep1 and GXpep2 bind to a site far from the active center of GPX4, do not induce conformational changes, and are inactive. In contrast, GXpep3 occupies a cavity close to the catalytic site and inhibits GPX4 activity with an IC_50_ of 10 µM (Figure 4). The interaction of GXpep‐3 and GPX4 involves (i) hydrogen bonds between the C‐terminal residue (Lys14) of GXpep3 and the main‐chain of Ile156 and side‐chain of Lys162 and Arg179 in GPX4, (ii) hydrophobic interactions between the Trp9 side chain of GXpep‐3 and Trp163 and Pro182 of GPX4 as well as (iii) a water‐mediated hydrogen bond between the Tyr5 side chain of the inhibitor and the main‐chain of Asn164 in GPX4 close to the active center. Peptides with substituted Tyr5 side chain that covalently target selenocysteine Sec73 have been proposed to possess superior GPX4 inhibitory activity,^180^ which is in line with the TCI concept described in Section 5.^134^ Thus, the noncovalent interactions of the peptide with GPX4 might align the inhibitor in the binding cleft, thereby favoring a subsequent reaction between the electrophilic group and the protein.

#### 6.1.4 Parthenolide derivative DMOCPTL (**41**)

The naturally occurring anticancer germacranolide‐type sesquiterpene lactone parthenolide (**40**) was used as a template to design **41**, which has, like **40**, an exocyclic α,β‐unsaturated carbonyl function as well as an epoxide as reactive groups (Figure 3).^181^ Compound **41** reduces the viability of the human triple‐negative breast cancer cell lines MDA‐MB‐231 and SUM159 (EC_50_ = 0.2–0.5 µM). The ferroptosis inhibitors deferoxamine (DFO) and ferrostatin‐1 as well as the apoptosis inhibitor Z‐VAD‐FMK attenuated the cytotoxic effect of **41**, whereas inhibition of autophagy (3‐methyladenine [3‐MA]) and necroptosis (necrostatin‐1) was without effect. While the induction of apoptosis by **41** was ascribed to an upregulation of EGR1, the drop in GPX4 protein levels seems to be responsible for ferroptosis induction.^181^ Of note, the transcription factor EGR1 is known to be a negative regulator of GPX4 protein expression.^71^ Compound **41** directly binds to GPX4 and promotes ubiquitination and subsequent degradation of the enzyme, as confirmed by pull‐down assays and GPX4/compound **41** colocalization studies using a fluorescence‐labeled derivative of **41**. Docking studies suggest that **41** is tightly bound to the active site of GPX4 with the styryl ring of **41** extending into a hydrophobic pocket. Proposed key interactions between **41** and GPX4 are (i) strong hydrophobic interactions of the styryl ring with Trp163 and Pro182 of GPX4 and (ii) hydrogen bonds between the oxygen of the methoxy group and the Lys162 and Ile156 side chains of GPX4. Whether **41** has additional targets besides GPX4 that contribute to ferroptosis induction is unknown. Speculations about further targets are supported by the parental compound **40** that activates NRF2 (Figure 3)^132^, ^182^, ^183^ and inhibits thioredoxin reductase (IC_50_ = 2.5–5 µM).^184^ Inhibition of the latter by **40** has been linked to apoptosis in human Hela cervix carcinoma cells.^184^ Considering the important role of the thioredoxin/thioredoxin reductase system in GSH regeneration and protection from ferroptosis,^3^, ^185^ we speculate that the ferroptosis‐inducing activity of **40** and potentially **41** at least partially relies on the inhibition of these pathways. Due to the poor pharmacokinetics of **41**, a prodrug (**42**, Figure 3) was subjected to preclinical studies. In mice bearing tumors derived from mouse 4T1 breast cancer cells, the prodrug **42** (1 mg/kg, iv, 6× every other day) decreased Gpx4 and increased Egr1 protein levels and inhibited tumor growth. Oral application (50 mg/kg) of **42** prolonged survival without any obvious toxic effects (drop in body weight). Together, compound **42** is a promising candidate for the development of drugs directed against breast cancer, which might include aggressive, metastatic forms like triple‐negative tumors.

#### 6.1.5 Altretamine (**43**)

The FDA‐approved anticancer drug **43** was recently identified as a GPX4 inhibitor by network perturbation analysis, which combines hybrid computational and experimental studies (DeMAND) and aims at elucidating genome‐wide molecular mechanisms of small molecules.^186^ Compound **43** inhibits the GPX4‐dependent reduction of phosphatidylcholine hydroperoxide in a cell‐free assay, although at a relatively high concentration (500 µM).^186^ Considering that **43** is an alkylating agent, the GPX4‐inhibitory activity might derive from the covalent modification of selenocysteine in the active center.

#### 6.1.6 Withaferin A (**44**)

*Withania somnifera* and other members of the Solanaceae family (e.g., *Acnistus arborescens*) possess anti‐inflammatory, antioxidative, and stress‐adaptive properties and are traditionally used in ayurvedic medicine to treat tumors and ulcers.^187^ The bufadienolide **44** (Figure 3), isolated from the roots of these plants, covalently binds to GPX4 at micromolar concentrations (10 µM).^188^ Mutagenesis studies indicate that **44** interacts preferentially with cysteines but not with the selenocysteine of GPX4.^188^ Compound **44** exhibits three reactive sites, which are attacked by nucleophilic cysteines, two α,β‐unsaturated carbonyl groups, and a highly reactive epoxide. Moreover, compound **44** affects various other pathways (e.g., NRF2 and NF‐κB signaling, cytoskeleton dynamics, cell cycle regulation, and kinase cascades), whose contribution to ferroptotic cell death versus other forms of cell death remains to be investigated.^189^

#### 6.1.7 Comparative discussion

Taken together, potent GPX4 inhibitors have been identified that covalently bind to selenocysteine in the active center. Since these inhibitors require reactive electrophilic groups with rapid reaction kinetics and hardly engage noncovalent interactions, they lack selectivity and metabolic stability. To date, none of the GPX4 inhibitors is applicable for in vivo use due to low solubility and poor pharmacokinetics.^31^ To circumvent these drawbacks, pro‐drugs have been developed that are intracellularly converted into electrophiles, however at the expense of potency.^4^ GPX4 has a flat binding pocket, which allows the enzyme to accept bulky phospholipids (embedded into bilayers) as substrates. Identification of a conventional small‐molecule inhibitor that forms noncovalent interactions with GPX4 might therefore be challenging. A large database screen with over 1,000,000 compounds actually identified only nine electrophilic inhibitors that covalently bind to the catalytic selenocysteine of GPX4. Additional noncovalent interactions were low or absent. Note that inhibitors that simply rely on an electrophilic moiety for GPX4 inhibition are poorly compatible with selectivity, as confirmed by cysteinome analysis.^4^ On the other hand, it should be taken into account that databases used for screening rather include conventional small molecule inhibitors, whereas noncovalent inhibitors of GPX4 might demand structural features that are more complex and not covered. GPX4 inhibition by the peptide GXpep3 indicates that the development of noncovalent inhibitors of GPX4 is feasible and also the parthenolide derivative seems to form noncovalent interactions with GPX4 according to modeling studies.^180^, ^181^ Noncovalent binding to GPX might be accomplished by mimicking either the cosubstrate GSH, which is a small peptide containing three amino acids, or the natural substrates of GPX4, that is, oxidized PUFAs. The high chemical diversity of natural products might make a difference and pave the way to success in this challenging field. Promising for targeting GPX4 appear peptide‐like structures and oxylipins, in particular when combined with electrophilic warheads, such as α,β‐unsaturated ketones.

### 6.2 Indirect inhibitors of GPX4

Interference with GPX4 expression is an indirect strategy to lower GPX4 activity. Inhibitors of NRF2, which we discuss in detail in Section 8 employ this mechanism. The small molecules CIL56 (**45**) and FIN56 (**46**) (Figure 3) also lead to a drop in GPX4 protein levels but engage a different mechanism. The two compounds potently induce ferroptotic cell death in human HT1080 fibrosarcoma cells (EC_50_ = 0.1–0.4 µM), with **46** being more selective toward oncogenic H‐RAS‐mutant cells (H‐RAS^G12V^).^174^ Both compounds stimulate GPX4 degradation by a nonproteasomal mechanism, which might involve the activation of acetyl‐CoA carboxylase (ACC) 1, as suggested from compensation studies using the acetyl‐CoA carboxylase ACC1 inhibitor 5‐tetradecyloxy‐2‐furonic acid.^100^, ^174^ The exact mechanisms remain elusive. Compound **46** also activates squalene synthase (Figure 1), thereby depleting coenzyme Q10. Further studies are required to evaluate the potential of **45** and **46** for in vivo application.^31^ Note that the natural product derivative FINO2 (**97**) described in Section 9.1 also indirectly inhibits GPX4 activity.

### 6.3 Combined indirect GPX4 inhibitors and HO‐1 inducers

The role of NRF2 activation on ferroptosis is bivalent. NRF2 activators have been described to counteract lipid peroxidation and ferroptosis^190^ but also to have antitumoral activity associated to ferroptotic cell death.^48^ The latter is counterintuitive since ferroptosis relies on the oxidative damage of cellular lipids against which classical NRF2 target genes provide protection.^138^ The enigmatic mechanisms behind the proferroptotic activity might be related to the point of attack of NRF2 activators within the NRF2 signaling pathway, the responsiveness of the selected model system, cell type‐specific NRF2 target gene profiles, or off‐target effects. Among the critical determinants that define the cellular fate upon NRF2 activation is HO‐1 as well as a simultaneous suppression of GPX4 levels. The NRF2‐target gene HO‐1 degrades heme to biliverdin/bilirubin, carbon monoxide, and ferrous iron, which then regulate cell death or mediate cytoprotection.^191^ The ferroptosis‐inducing activity of HO‐1 predominantly depends on the release of ferrous iron, whereas biliverdin, bilirubin, and carbon monoxide have overall antioxidant, antiapoptotic, anti‐inflammatory, and stress‐protective activity.^192^ HO‐1 seems to be a double‐edged sword, with both tumor‐inhibiting and ‐promoting activity depending on the metabolic status of the cancer cells and the tumor microenvironment. Studies on the pharmacological or genetic induction of HO‐1 support this hypothesis. On the one hand, HO‐1 expression enhanced ferroptotic cancer cell death upon cystine deprivation (compound **18**).^193^ On the other hand, HO‐1‐stimulating small molecules rather had the opposite effect and prevented ferroptosis in noncancer cells.^194^, ^195^

#### 6.3.1 Curcumin (**47**) and derivatives **48** and **49**

The yellow dye from curcuma, **47** (Figure 5), is a Michael acceptor and pan–assay interference compound, for which a broad spectrum of activities has been described in vitro and in vivo, including antioxidative, anticancer, and antimetastatic properties.^196^ Compound **47** induces cell death in a variety of cancer cell lines, mainly by inducing apoptosis. Ferroptosis has recently been suggested to contribute to cell death induction by **47** in human breast cancer cell lines (EC_50_ = 50–100 µM), based on the protective effect of ferrostatin‐1 and DFO.^197^ Thus, compound **47** induced the accumulation of intracellular iron, enhanced lipid peroxidation, decreased glutathione levels, and regulated ferroptosis target genes on a transcriptome level. Accordingly, the piperidone‐based C5‐curcuminoid EF24 (**48**) (Figure 5) (EC_50_ = 1–2 µM) and the cyclobutanmethyl‐substituted C7‐curcuminoid ALZ003 (**49**) (Figure 5) (at 2–10 µM) decreased the viability of human U2OS and Saos‐2 osteosarcoma cells^198^ and human U87MG glioma cells,^199^ respectively, but did not show cytotoxic effects on primary human astrocytes.^199^ While **49** also induced apoptosis at micromolar concentrations (2–5 µM) in addition to ferroptosis,^199^ the cytotoxic effects of **48** were exclusively diminished by ferrostatin‐1 but neither by apoptosis (Z‐VAD‐FMK), pyroptosis (necrosulfonamide), nor autophagy inhibitor (MRT68921).^198^ Cell death induction by **49** was ascribed to ubiquitination‐dependent degradation of AR, which represses GPX4 expression and subsequently raises lipid hydroperoxide levels.^199^ Knockdown of AR induced lipid hydroperoxide formation, whereas overexpression upregulated GPX4 and had the opposite effect. Importantly, AR expression is elevated in human glioblastoma compared to normal brain tissue and further increased in temozolomide‐resistant cells. Accordingly, both temozolomide‐sensitive and ‐resistant glioblastomas were significantly inhibited by **49**, while cytotoxic effects were not observed in normal astrocytes.^199^

Figure 5Combined indirect glutathione peroxidase 4 inhibitors and heme oxygenase‐1 inducers. PEITC, phenylethyl isothiocyanate; TCBQ, tetrachloro‐1,4‐benzoquinone. [Color figure can be viewed at wileyonlinelibrary.com]

**Figure:**
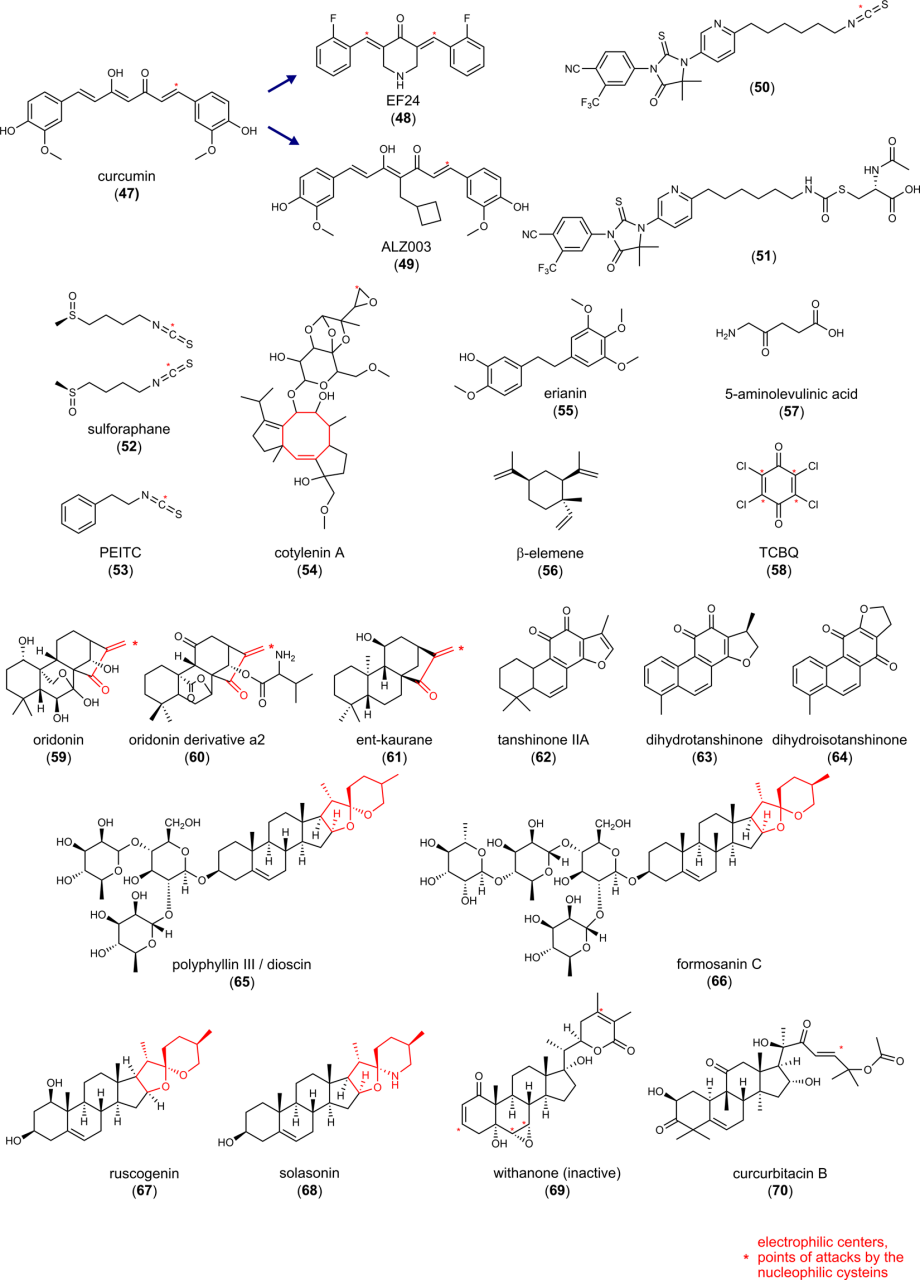

**Figure:**
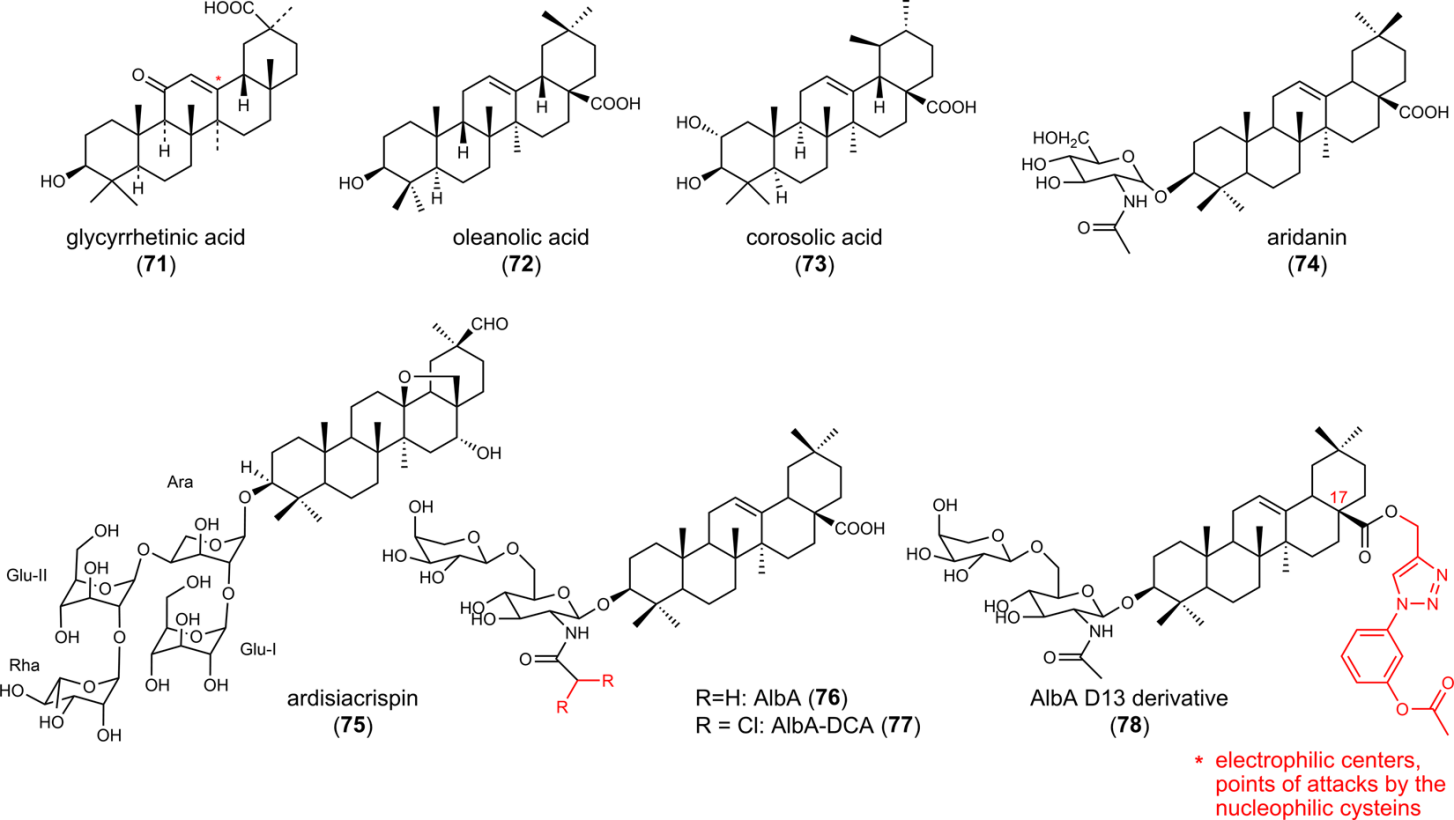

#### 6.3.2 Hybrid AR antagonists (**50** and **51**)

The AR is known to (i) contribute to redox homeostasis,^200^ (ii) be upregulated in tumor tissues, and (iii) promote drug resistance,^201^ but its role in ferroptosis is insufficiently understood. The combination of structural elements for AR inhibition (enzalutamide) and AR degradation (PEITC, **53**) yielded isothiocyanate **50** (Figure 5), which was further optimized to the *N*‐acetylcysteine derivative **51** (Figure 5).^202^ Within 72 h, the hybrid compounds reduced the viability of human VCaP prostate cancer cells that abundantly express full‐length wild‐type AR as well as a splice variant (**50**: EC_50_ = 3.9 µM; **51**: EC_50_ = 6.1 µM). Compound **51** decreased the expression of the AR, elevated free iron levels, induced lipid peroxidation, and triggered the expression of the NRF2 target gene HO‐1. Cotreatment with the GSH biosynthesis inhibitor buthionine sulfoximine (BSO) (to compensate for higher GSH biosynthesis and regeneration upon NRF2 activation) enhanced these effects and showed pronounced cytotoxic activity already within 24 h, which was prevented either by ferrostatin‐1 or the iron chelator DFO. As described for **79**, the upregulation of HO‐1 seems to be central for the ferroptosis‐inducing activity of **51**, as the HO‐1 inhibitor zinc protoporphyrin (ZnPP)‐9 diminished the response. Qin et al.^202^ speculated that the combined treatment of **51** and BSO unfolds its anticancer properties by downregulating the AR and inducing ferroptosis by NRF2‐dependent expression of HO‐1.

Most recently, overexpression and knockdown studies revealed AR as a major regulator of GPX4 expression in the luminal AR (LAR) subtype of triple‐negative breast cancer cells, which strongly express AR and GPX4.^203^ AR has been proposed to directly bind to the GPX4 promotor. Based on these findings, we speculate that impaired GPX4 expression contributes to the ferroptosis‐inducing activity of compounds **50** and **51**.

These findings strengthen the mechanistic link between AR signaling, GPX4, and ferroptosis and we anticipate that AR inhibition and degradation also contribute to the ferroptosis‐inducing activity of the hybrid isothiocyanate‐ and *N*‐acetylcysteine‐containing AR antagonists **50** and **51** described above. Compound **48** seems to engage a similar mechanism, given the decrease of GPX4 protein expression.^198^ On the other hand, compounds **50** and **51** induced ferroptosis by upregulating HO‐1,^202^, ^204^ and also **47** and **48** enhanced HO‐1 expression,^198^ which closes another gap. Based on these studies, we hypothesize that hybrid isothiocyanate‐containing AR antagonists (**50, 51**) and curcuminoids (**47**–**49**) share a common ferroptotic mechanism: GPX4 is downregulated through suppression of AR (and potentially further mechanisms), while HO‐1 expression is induced, likely as a result of impaired AR signaling and/or NRF2 activation. Note that **47** is a KEAP1 inhibitor that protects NRF2 from proteasomal degradation.^205^ In a murine xenograft model using intracranially transplanted wild‐type and temozolomide‐resistant human U87MG glioblastoma cells, compound **49** (20–80 mg/kg, iv) significantly reduced tumor size and prolonged survival.

#### 6.3.3 Sulforaphane (**52**)

Mustard oil glycosides are abundant in cruciferous vegetables and taken up by food.^134^, ^206^ When getting in contact with the enzyme myrosinase upon cell damage, mustard oil glycosides are converted into isothiocyanates. These electrophiles have manifold targets and, amongst others, reversibly bind to KEAP1 at Cys151.^134^, ^206^ The isothiocyante **52** (Figure 5) is a major component responsible for the proposed anticarcinogenic effects of broccoli (*Brassica oleracea*, Brassicaceae)^207^ and represents one of the most potent natural activators of the NRF2 pathway. Preclinical in vivo studies suggest (neuro)protective effects of **52** in Parkinson's disease (5 mg/kg, ip),^208^ Huntington's disease (0.5 mg/kg, daily injection),^209^ Alzheimer's disease (30 mg/kg, ip, daily)^210^ and multiple sclerosis, as well as traumatic injuries (5–50 mg/kg, ip).^211^ In addition to these protective effects on nontransformed cells, **52** triggers the death of cancer cells from different origins.^212^ In small‐cell lung cancer, compound **52** (20 µM) induced lipid peroxidation and ferroptosis, which was diminished by ferrostatin‐1 and DFO.^212^ Cytotoxic effects of **52** on normal 16 HBE bronchial epithelial cells were instead very low. Along these lines, compound **52** decreased the viability of cancerous but not normal prostate epithelial cells.^213^ The exact mechanisms responsible for the selective lethality of the electrophilic compound **52** against cancer cells remain elusive. We suggest that **52** differentially affect redox‐dependent signaling pathways depending on the cellular iron and redox status. In this context, compound **52** increased the cellular iron pool, depleted GSH, and decreased the expression of the NRF2 target gene SLC7A11.^212^ Given that **52** is a potent NRF2 activator^214^ that strongly induces HO‐1 expression,^213^ it is tempting to speculate that **52** unfolds its ferroptosis‐inducing activity in cancer cells, at least partially, by upregulating HO‐1.

#### 6.3.4 Phenylethyl isothiocyanate (**53**)

The phenylethy isothiocyanate **53** (Figure 5) is prevalent in cruciferous vegetables and commonly found in *Brassica species*.^215^ Antioxidative, anti‐inflammatory, and cytoprotective activities are well documented for compound **53**; the latter in diverse in vitro (20 µM) and in vivo (1 mg/kg, −12.5 mg/kg, ip) organ injury models.^216^, ^217^ On the other hand, compound **53** induces NRF2 signaling,^218^ as known for other isothiocyanates, and triggers multiple forms of cell death, including ferroptosis in murine K7M2 osteosarcoma cells.^219^ Inhibitors of ferroptosis (ferrostatin‐1, liproxstatin‐1, DFO), apoptosis (Z‐VAD‐FMK) and necroptosis (necrostatin‐1) diminished the cytotoxic effects of **53** (EC_50_ = 30 µM) to a similar extent, whereas inhibition of autophagy (bafilomycin A1, 3‐MA) was less effective. Treatment with **53** resulted in ROS formation and lipid peroxidation, increased the labile iron pool, and lowered cellular GSH levels. Mechanistic studies revealed an enhanced ferritin heavy chain (FTH) 1 and SLC7A11 messenger RNA (mRNA) and decreased GPX4 protein expression but did not address HO‐1.^219^ It was found in another study that **53** (10 µM) induces HO‐1 expression in human HCT116 human colon adenocarcinoma cells, which was blocked by coincubation with the iron chelator DFO. At high dosage, compound **53** (60 mg/kg, ig, once daily) reduced tumor weight in a K7M2 xenograft mouse model.^219^ Increasing the dose to 90 mg/kg surprisingly lowered the antitumoral efficacy. Clinical trials (phase I–III, 2004‐2011) indicate that **53** is well‐tolerated (www.clinicaltrials.gov, NCT00968461, NCT00005883, NCT00691132, NCT01790204, NCT03700983, NCT03034603, and NCT02468882).

The fusicoccane diterpene glycoside cotylenin A **(54)** (Figure 5) isolated from the fungus *Alternaria brassicicola* ^215^ sensitizes cancer cells to **53**. Treatment of human MIAPaCa‐2 and PANC‐1 pancreatic cancer cells with either **53** (4–6 µM) or **54** (24–32 µM) moderately triggered ROS production (twofold to fivefold), but only **53** decreased cell viability (by 40%). The combination of **53** and **54** showed strong synergistic effects, both inducing ROS formation (20‐fold) and inducing cell death (80%). Cytotoxic effects were prevented by *N*‐acetyl cysteine or the water‐soluble α‐tocopherol derivative trolox, and cell death induction was significantly attenuated by the membrane radical trapping agents ferrostatin‐1 and liproxstatin‐1 or the iron chelator DFO. Inhibition of other forms of cell death like apoptosis (Z‐VAD‐FMK, Q‐VD‐OPH), necroptosis (necrostatin‐1), or autophagy (3‐metyladenine and chloroquine) was not effective, which indicates that the cytotoxic effects of a combinatory treatment with **53** and **54** selectively unfolds its activity by inducing ferroptotic cell death.

#### 6.3.5 Erianin (**55**)

The substituted dibenzylic compound **55** (Figure 5), isolated from *Dendrobium chrysotoxum*, substantially reduced the viability and slightly the proliferation of human H460 and H1299 lung cancer cells at concentrations of 50–100 nM.^220^ However, compound **55** also has cytoprotective activities, as shown for normal rat NRK‐52E kidney epithelial cells under high glucose‐induced oxidative injury.^221^ In cancer cells, low concentrations of compound **55** (12.5–100 nM) augmented intracellular ROS and MDA levels, depleted intracellular GSH, and decreased the expression of GPX4, SLC7A11, and SLC40A1. Very high concentrations of compound **55** (320 µM) additionally lowered the expression of NRF2 as well as of the NRF2 target proteins FTH1 and HO‐1.^222^

Cotreatment with cysteine derivatives (N‐acetyl cysteine or GSH) and ferroptosis inhibitors (liproxstatin‐1 or ferrostatin‐1) diminished the drop in cell viability and proliferation by **55**,^220^, ^222^ whereas inhibition of apoptosis (Z‐VAD‐FMK), necroptosis (necrostatin‐1), or autophagy (chloroquine) failed to suppress the cytotoxic effect.^222^ Interestingly, the calmodulin (CaM) antagonist calmidazolium attenuated the inhibitory effect of **55** on the proliferation of lung cancer cells, which indicates that changes in Ca^2+^/CaM signaling regulate **55**‐induced ferroptosis.^220^ Compound **55** is effective in vivo and significantly suppressed tumor growth at high doses (100 mg/kg, ip, once daily) in murine xenograft models, either using H460^220^ or human KU‐19–19 bladder carcinoma cells.^222^

Since **55** has been reported to enhance the expression of NRF2 and its target genes HO‐1, SOD1, and SOD2 in vivo (20 mg/kg, ip, once daily),^223^ we consider it likely that the NRF2/HO‐1 axis contributes to the ferroptotic mechanism of the compound. Whether the NRF2/HO‐1 pathway is activated in vivo (as shown in vitro for low and potentially pharmacologically relevant **55** concentrations) or downregulated (as observed for high compound concentrations) remains unclear. In this context, the expression of two other (ferroptosis‐related) NRF2 target genes, GPX4 and SLC7A11, is decreased by **55** in cell‐based studies (at low concentrations)^220^ and in tumors from murine xenografts.^222^ Such an opposing regulation of SLC7A11 and GPX4 relative to major NRF2 target genes (GCLC, GCL modifier subunit [GCLM], NAD(P)H quinone dehydrogenase 1 [NQO1], HO‐1) has, however, previously been observed, for example, for phorbol 12‐myristate 13‐acetate (PMA)‐treated human THP‐1 monocytic leukemia cells,^224^ and is not necessarily an indicator for NRF2 inhibition.

#### 6.3.6 β‐Elemene (**56**)

The natural sesquiterpene **56** (Figure 5) is prevalent in diverse medicinal plants, such as the Chinese herb *Curcuma wenyujin*.^225^ At high concentrations (612 µM), **56** induced cell death in K‐RAS‐mutant HCT116 colorectal cancer cells (CRC) when combined with cetuximab (25 µg/ml), a monoclonal antibody against epidermal growth factor receptor (EGFR). Cytotoxic effects were strongly reduced by the ferroptosis inhibitors ferrostatin‐1, liproxstatin‐1, or DFO but neither by the apoptosis inhibitor Z‐VAD‐FMK, the necroptosis inhibitor necrostatin‐1 nor the autophagy inhibitor 3‐MA. The combinatory treatment with **56** and cetuximab depleted intracellular GSH, downregulated GPX4, SLC7A11, and SLC40A1 expression, and increased ROS and MDA levels. Expression of HO‐1, another NRF2 target gene, was instead upregulated, which is in line with our assumption that the NRF2/HO‐1 axis contributes to ferroptosis induction. Compound **56** also affects multiple other signaling pathways with important roles in tumorigenesis, among others Wnt‐β‐catenin (at 200 µM), Notch (at 250 µM), mitogen‐activated protein kinase (MAPK; at 100–400 µM), and autophagy cascades (at 50–250 µM), which likely contribute to the overall antitumoral activity. In an orthotopic colon cancer xenograft model in mice, compound **56** together with cetuximab (50 mg/kg, iv, every 6 days, each) triggered cell death of drug‐resistant cancer cells (HCT116‐luc cells) and improved survival.^225^ Mechanistically, the combination of **56** and cetuximab lowered the expression of GPX4, SLC7A11, and SLC40A1 in grafted K‐RAS‐mutant HCT116 CRC cells.^225^ Compound **56** is currently under investigation in phase 2 and 3 clinical trials for the treatment of cancer, including glioblastoma and lung cancer, either as monotherapy or in combination with tyrosine kinase inhibitors to treat tumors with acquired drug resistance (www.clinicaltrials.gov, NCT02629757, NCT03123484). Conclusively, **56** has pronounced anticancer properties but it is unknown to which extent ferroptosis contributes to cell death induction in vitro and in vivo.^226^

#### 6.3.7 5‐Aminolevulinic acid (**57**)

The naturally occurring, nonproteinogenic amino acid **57** (Figure 5) has anticancer properties and is in clinical use for photodynamic therapy of glioblastoma, skin, and bladder cancer.^227^, ^228^, ^229^ Cancer cells preferentially metabolize **57** into the photoactive protoporphyrin IX, which produces ROS upon light irradiation, whereas nontransformed cells lack the machinery for the effective conversion of **57** into the toxic metabolite.^230^ Note that **57** decreased cancer cell survival at high concentrations (EC_50_ = 200–500 µM) also without being photoactivated. Direct cytotoxic effects of **57** on human KYSE‐30 esophageal squamous carcinoma cells (EC_50_ = 400 µM) were attenuated by ferrostatin‐1 but not by the apoptosis inhibitor Z‐VAD‐FMK. Despite the high effective concentrations in vitro, oral gavage of **57** (30 mg/kg, daily) efficiently reduced the growth of KYSE‐30‐grafted tumors in mice, which is likely related to the favorable bioavailability and high turnover of amino acids by cancer cells. Mechanistically, **57** decreased GPX4 and increased HO‐1 protein expression both in vitro (at 50–100 µM) and in vivo (at 30 mg/kg, po, daily).

#### 6.3.8 Tetrachloro‐1,4‐benzoquinone (**58**)

The chlorinated benzoquinone **58** (Figure 5) increased MDA levels, elevated the labile iron pool, and induced cell death in rat PC12 adrenal gland cells (40% cell viability at 20 µM), which was diminished by ferrostatin‐1.^231^ Compound **58** has previously been reported to activate the NRF2 pathway^232^ and to increase SLC7A11 and FTH1 and decrease GPX4 expression.^231^

#### 6.3.9 Oridonin (**59**)

The ent‐kaurene‐type diterpenoid **59** (Figure 5), a major active component of extracts from *Isodon rubescens*,^233^ shares the saturated phenanthrene scaffold and the α,β‐unsaturated ketone bridge with **61**. Compound **59** (27 µM) decreased the viability of human TE1 esophageal cancer cells (by 50%), which was diminished by cotreatment with DFO,^233^ and inhibited the growth of esophageal squamous cell carcinoma in vivo in patient‐derived xenografts (40 mg/kg, po).^234^ Labile iron, MDA, and ROS levels were significantly increased by **59** (15–30 µM), while GPX4 activity and GSH/glutathione disulfide (GSSG) ratios were decreased.^233^ Cytotoxic effects of **59** seem to depend on the inhibition of the γ‐glutamyl cycle, which is important for GSH regeneration.^235^ Thus, **59** formed covalent adducts with free cysteine in a cell‐free assay and inhibited the activity of γ‐glutamyl transpeptidase 1 (GGT1), which converts extracellular GSH to cysteinylglycine—a crucial step for the reuse of GSH‐derived amino acids for intracellular GSH biosynthesis.^235^ Whether cysteine adducts serve as inhibitory product mimetics of GGT1 or whether **59** directly binds to GGT1 or other proteins with exposed nucleophilic (seleno)cysteines has not been addressed. In support of the latter hypothesis, compound **59** was identified as a weak inhibitor of thioredoxin reductase (IC_50_ = 50–100 µM).^236^ Note that multiple other mechanisms independent from ferroptosis have been described for **59**, which might add to its antitumoral properties. For example, compound **59** interferes with the cell cycle, apoptotic pathways, and autophagy at micromolar concentrations (15–54 µM) in various cancer cell lines.^234^, ^237^

#### 6.3.10 Oridonin derivative a2 (**60**)

The valinyl ester **60** (Figure 5), which differs from **59** by the degree and position of ring oxygenation, reduced cell proliferation in gastric cancer cell lines (GC_50_ = 1–15 µM) in an iron‐dependent manner.^238^ Similar to the parental compound **59** the derivative **60** (at 2.5–10 µM) increased labile iron levels and lipid ROS production and decreased GPX4 expression. Other NRF2 target genes like SLC7A11, HO‐1, GCLC, and FTH1 were instead upregulated. Compound **60** (5–20 mg/kg, iv) reduced the tumor size in patient‐derived tumor xenograft mouse models on GC (by 30%–80%), being more efficient than 5‐fluoro uracil.

#### 6.3.11 ent‐Kauranes (**61**)

The *ent*‐kaurane family (Figure 5) comprises complex tetracyclic diterpenoids that are found in liverwoods (*Jungermannia species*) as well as higher plants (*Isodon species*).^239^ Compound **61** and analogues induced cell death in various cell lines (EC_50_ = 0.9–5.1 µM), including human A549 lung carcinoma cells, HepG2 hepatocarcinoma, HBE bronchial epithelial, 768‐O renal adenocarcinoma, and A2780 ovarian cancer cells.^239^ Ferroptosis and apoptosis contribute to cell death to a similar extent, as suggested by inhibitor studies using ferrostatin‐1, DFO, and Z‐VAD‐FMK. The α,β‐unsaturated ketone is essential for the cytotoxic activity of **61** and directly reacts with GSH. Compound **61** accordingly lowered cellular GSH (EC_50_ = 2.5 µM) and raised lipid hydroperoxide levels. In addition, **61** interacts with PRDX1/2, as shown for a biotinylated derivative by immunoprecipitation. Compensation studies in PRDX1/2‐overexpressing A549 cells suggested PRDX1/2 as a functional target of **61**. When combined with cisplatin (32.5 µM), compound **61** (1.25 µM) synergistically induced membrane peroxidation and cell death in vitro. Accordingly, coadministration of **61** (10 mg/kg, ip) and cisplatin (4 mg/kg, ip) every 3 days showed superior antitumoral efficacy in a cisplatin‐resistant A549 cell xenograft model in mice.

In summary, the ent‐kauranes **59–61** share an exocyclic α,β‐unsaturated ketone, which predestines them to interact with (seleno)cysteine redox switches in proteins. The physiological relevance of such modifications is supported by recent studies showing that compound **61** forms covalent adducts with PRDX1/2 and GSH, respectively. Less understood is whether electrophilic ent‐kauanes share a common ferroptotic mechanism. Recent reports suggested that (i) compound **61** targets PRDX1/2, (ii) compound **59** inhibits the activity of GGT1, and (iii) its derivative **60** downregulates GPX4 expression. In addition, the major ferroptosis regulator NRF2 is an established target of kaurane diterpenoids (5–25 µM)^240^, ^241^ but rather considered to mediate their organ‐protective functions against injury.^242^ Given the bivalent function of NRF2 in ferroptosis, we propose that excessive activation of the NRF2/HO‐1 axis along with GPX4 repression by ent‐kauranes instead initiates cell death. Experimental proof for this hypothesis is lacking so far. In view of the close structural similarity of **59–61** together with the fact that the studies on **61**,^239^ **59**,^233^ and **60** ^238^ did not exclude other putative targets, we further speculate that ent‐kauranes unfold their ferroptosis‐inducing activities through a common mechanism, namely by covalently targeting redox‐regulated proteins, including PRDX1/2, GGT1, NRF2, and TXNRD.

#### 6.3.12 Tanshinone IIA (**62**)

The partially saturated phenanthrofuran **62** (Figure 5) from the Chinese herb *Salvia miltiorrhiza* (Danshen) induced cell death in human GC cells, that is, BGC‐823 (EC_50_ = 2.8 µM) and NCI‐H87 cells (EC_50_ = 3.1 µM)^243^—effects that were diminished by ferrostatin‐1. Compound **62** increased lipid hydroperoxide levels, lowered SLC7A11 protein expression, and reduced cellular GSH concentrations, which was ascribed to an upregulation of p53. Silencing of p53 strongly diminished these proferroptotic adaptions and prevented compound **62**‐induced ferroptotic cell death. These studies point to a major role of p53 in mediating the cytotoxic effect of **62**. In a BGC‐823 xenograft mouse model, compound **62** (50 mg/kg, ip every other day) significantly reduced the tumor weight (2.5‐fold), upregulated p53 expression and lipid peroxidation, and decreased GSH levels. Effects of **62** on tumor growth and ROS production were prevented by ferrostatin‐1 in vivo, which strongly suggests that the mechanism of **62** relies on the induction of ferroptotic cell death. On the other hand, compound **62** also stimulated apoptosis in human MGC803 and SGC7901 GC cells at comparable concentrations (EC_50_ = 1.5 µM)^244^ than needed to trigger ferroptosis.^243^ These data suggest that compound **62** induces a mixed cell death program of apoptosis and ferroptosis, with a preference for one or the other mechanism dependent on the cell line. Causative for the decrease in cancer cell stemness is the induction of ferroptosis, at least in SGC‐7901 and BGC‐823 GC cells at high concentrations of **62** (EC_50_ = 250–550 µM).^245^ Cancer stem cells promote tumor reconstitution and propagation and are linked to chemoresistance, cancer recurrence, and metastasis.^246^ Three clinical trials currently investigate the efficacy of **62** in the treatment of polycystic ovary syndrome (NCT01452477), pulmonary hypertension (NCT01637675), and acute myocardial infarct (NCT02524964) (www.clinicaltrail.gov). A fourth study investigates an extract enriched in **62**, tetraarsenic tetrasulfide, and indirubin in promyelocytic leukemia (NCT02200978). Besides being an inductor of ferroptosis or apoptosis, compound **62** (at 50 nM) also potently inhibited **18**‐ and **23**‐induced ferroptotic cell death, namely in normal human coronary artery endothelial cells by activating NRF2 signaling.^247^ This finding underscores the beneficial bivalent properties of NRF2 activators in normal versus cancer cells.

#### 6.3.13 15,16‐Dihydrotanshinone I (**63**)

Another bioactive compound from the tanshinone series, **63** (Figure 5), was isolated from tanshen (*Salvia miltiorrhiza*)^248^ and differs from **62** by the degree of unsaturation and a number of methyl substituents. Compound **63** inhibited cell proliferation in human U251 and U87 glioma (GC_50_ = 10 µM),^248^ breast cancer (GC_50_ = 1 µM),^249^ colon cancer (GC_50_ = 12.5 µM),^250^ and GC cells (GC_50_ = 12.5 µM),^251^ but seems to be less potent than **62**, at least in GC cells, where a direct comparison is possible. Studies on glioma cells showed that protein expression of GPX4, as well as cellular GSH/GSSG ratios, are significantly decreased by **63**, while ACSL4 expression and MDA levels were elevated, though both effects only manifested at high concentrations of **63** (100 µM).^248^ The **63**‐induced drop of GPX4 and GSH and increase in MDA levels was reduced by ferrostatin‐1, which suggests that **63** combines low micromolar cytostatic activity with a moderate potential to induce ferroptosis in glioma cells. On the other hand, **63** rather induced apoptosis in breast and colon cancer cells^249^, ^250^ and suppressed cell proliferation in GC cells via a c‐Jun N‐terminal kinase/p38 MAPK‐dependent pathway.^251^ Because of the high concentrations of **63** (100 µM) that were applied to study ferroptosis as compared to the low EC_50_ values (1–12 µM) required to stimulate apoptosis, we consider the latter as the predominate form of cell death induced by **63**.

#### 6.3.14 Dihydroisotanshinone I (**64**)

While **62** and **63** have a phenanthro[1,2‐*b*]furan core, compound **64** (Figure 5) isolated from *Salvia miltiorrhiza* is a phenanthro[3,2‐*b*]furan.^252^ Compound **64** suppressed the proliferation of human breast cancer cell lines (MCF‐7 by 80%; MDA‐MB‐231 by 70%) at low micromolar concentrations (5 µM) within 24 h. Higher concentrations of **64** or prolonged incubation times were required to inhibit cell viability measured as mitochondrial dehydrogenase activity.^253^ Compound **64** (10 µM) significantly reduced the protein expression and activity of GPX4, decreased the cellular GSH/GSSG ratio, and substantially increased MDA levels,^252^ which suggests that ferroptosis contributes to cell death induction. However, compound **64** (5 µM) also rapidly activated caspase‐3, a major executive caspase in apoptotic cell death.^253^ Administration of **64** to mice (30 mg/kg, ip, every 2 days) effectively suppressed the growth of MCF‐7‐ and HCT‐116‐grafted tumors.^254^

Together, tanshinone derivatives have in common that they induce ferroptosis as well as apoptosis but there are also substantial differences. While **62** and **64** trigger both cell death programs to a similar extent at single‐digit micromolar concentrations, compound **63** shows signs of ferroptosis only at high concentrations (100 µM) and rather induces apoptosis. Of note, tanshinones have antioxidative and anti‐inflammatory properties and are cytoprotective by activating the NRF2 axis.^247^, ^255^

#### 6.3.15 Polyphyllin III/dioscin (**65**) and formosanin C (**66**)

The diosgenin saponins **65** and **66** (Figure 5) from *Paris formosana* and *Dioscorea species* have immunoregulatory, anti‐inflammatory, antibacterial, and anticancer properties.^256^, ^257^, ^258^ For compound **65**, it has been shown that cell death induction relies on multiple mechanisms, involving apoptosis, ferroptosis, autophagy, and pyroptosis.^258^, ^259^ The extent by which the different pathways contribute to cell death varies between cell lines. In human hepatocarcinoma cells (HepG2 and Hep3B, **66**, EC_50_ = 2.5–10 µM),^260^ melanoma cells (**65**, EC_50_ = 5 µM),^261^ and MDA‐MB‐231 breast cancer cells (**65**, 72 h: EC_50_ = 2.5 µM; 24 h: EC_50_ = 8 µM), ferroptosis was identified as major cell death pathway activated by **65** and **66**.^257^ The decrease in cell viability by **65** was accompanied by an accumulation of lipid hydroperoxides. Ferrostatin‐1 diminished the cytotoxic effect of **65**, whereas cotreatment with apoptosis (Z‐VAD‐FMK), necroptosis (necrosulfonamide), and autophagy inhibitors (3‐MA) was not effective.^257^ Why only the iron chelator ciclopirox but not DFO impaired breast cancer cell death by compound **65** is not fully understood.^257^ Cells treated with **65** develop typical morphological and molecular hallmarks of ferroptosis: the mitochondrial membrane density and lipid peroxidation increase, the number of mitochondrial cristae decrease, and GSH levels drop.^257^ Synergistic cytotoxic effects on melanoma cells were observed for the combination of **65** with various chemotherapeutic agents (rapamycin, cisplatin, dacarbazine, and vemurafenib). On the other hand, compound **65** only slightly affected the viability of normal human immortal keratinocytes (HaCaT cells).^261^

In melanoma cells, lipid peroxidation and iron accumulation by **65** seem to depend on the downregulation of ferroportin (an NRF2 target gene) and upregulation of transferrin; two major proteins in iron metabolism, which regulate the iron transport from and into cells.^261^ Ferroportin represents the sole cellular efflux transporter for nonheme iron,^37^ and transferrin is an iron transport protein that is critically involved in cellular iron supply.^262^ Interestingly, compound **65** hardly affected the cellular availability of two other iron regulatory NRF2 target proteins, the transferrin receptor, and ferritin,^261^ whereas another study observed more pronounced cytotoxic effects of **66** on HepG2 versus Hep3B cells and ascribed this difference to a lower expression of FTH1.^260^ Ferritin consists of two subunits, ferritin light chain (FTL) and FTH1, and is an iron storage protein that nucleates oxidized iron, thereby limiting iron for the Fenton reaction. While FTH1 has an iron‐binding site as well as ferroxidase activity, FTL lacks enzymatic properties.^260^, ^263^ Since FTH1 expression is associated with autophagy, the authors speculated that the cytotoxic effects of **66** are mediated by the induction of autophagy and ferritinophagy. Given that **66** differs from **65** only by an additional rhamnopyranoside in the glycon, the different responses on cellular ferritin levels are surprising, and it cannot be excluded that the apparent failure of **65** to decrease ferritin expression might be related to experimental issues, for example, the antibody specificity for either FTL or FTH. In fact, Lin et al.^260^ applied an antibody directed against total ferritin to investigate the effect of **65** on ferritin expression, whereas Xie et al.^261^ used an anti‐FTH1 antibody for studies on compound **66**.

The cytotoxic activity of **65** in human MDA‐MB‐231 breast cancer cells partially depends on the upregulation of ACSL4, as suggested by knockdown experiments.^257^ Moreover, **65** induced the expression of the system X_c_^−^ component SLC7A11 via the transcription factor Krüppel‐like factor (KFL) 4, which counteracts the induction of ferroptotic cell death. Silencing of either KLF4 or system X_c_^−^ or inhibition of SLC7A11 by **14** sensitized MDA‐MB‐231 cells to **65**‐induced ferroptosis. Administration of **65** (10 mg/kg, ip) to mice with grafted MDA‐MB‐231 cells accordingly reduced the tumor burden, and cotreatment with **14** (200 mg/kg) prevented the upregulation of KFL4 and SLC7A11 and exhibited superior antitumoral activity. Of note, KLF4 has recently been shown to activate the NRF2 axis,^264^ which raises the question of whether KLF4‐dependent NRF2 activation represents a common mechanism that favors induction rather than protection from ferroptosis by installing a specific NRF2‐target gene profile (low GPX4, high SLC7A11, and HO‐1). In support of this hypothesis, compound **65** decreased GPX4 protein levels. We speculate that **65** acts similar to the HO‐1‐inducers described above, even though the consequences of HO‐1 remain elusive.

As observed for other ferroptosis‐inducing NRF2 activators, compound **65** inhibited stress‐induced cell death in normal cells via the Nrf2/HO‐1‐axis, for example, in primary rat type II alveolar epithelial cells (115–230 nM),^265^ rat NRK‐52E kidney epithelial cells (115–230 nM),^266^ rat H9C2 cardiomyoblasts (60–230 nM),^267^ and mouse AML‐12 liver cells (60–230 nM).^268^ Animal models on organ injuries confirmed that **65** is, among others, protective against lung ischemia/reperfusion injury,^265^ doxorubicin‐induced nephrotoxicity,^266^ doxorubicin‐induced cardiotoxicity,^267^ and doxorubicin‐triggered hepatotoxicity.^268^

#### 6.3.16 Ruscogenin (**67**)

The pentacyclic triterpene **67** with spiroketal scaffold (Figure 5) from the root of *Ophiopogon japonicus* increased the labile iron pool, elevated ROS levels and induced ferroptosis in human pancreatic cancer cell lines (BxPC‑3, SW1990, PANC‑1, and AsPC‑1; EC_50_ = 10 µM).^262^ Accumulation of free iron in cancer cells was ascribed to an upregulation of transferrin and downregulation of ferroportin. The iron chelator DFO accordingly diminished the cytotoxic effect of **67**. In mice with grafted BxPC‐3 cells, compound **67** (5 and 10 mg/kg, iv, twice a week) efficiently reduced the tumor weight (by 25%–50%).

#### 6.3.17 Solasonine aglycon (**68**)

The aglycon of the major steroidal alkaloid solasonine (Figure 5) from *Solanum melongena* induced ferroptotic cell death in human HepG2 hepatocarcinoma cells (at 15 ng/ml = 0.036 µM), which was suppressed by cotreatment with ferrostatin‐1 or the iron chelator DFO.^269^ Compound **47** efficiently downregulated GPX4 protein expression, rose cellular ROS levels and inhibited at 15 mg/kg (single dose; application route is not given) tumor growth (4.5‐fold) in a murine xenograft model of HepG2 cells.

Note that the HO‐1‐inducing terpenoids **65, 66**, and **67** shares the spiroketal scaffold with **100** and **101** (see Section 9.3). The latter two compounds were reported to induce ferroptosis by iron chelation and ferritin degradation in lysosomes. Given the structural similarity, we assume that common ferroptosis‐inducing mechanisms exist for these spiroketals, which might include NRF2/HO‐1 activation and interference with iron metabolism, though the experimental proof is still lacking.

#### 6.3.18 Withaferin A (**44**)

In addition to its ability to directly bind to GPX4 (at 10 µM), compound **44** induced ferroptosis in human IMR‐32 neuroblastoma cells already at low concentrations (1 µM) by enhancing HO‐1 protein expression via NRF2.^188^ Inhibition of HO‐1 by ZnPP protected from compound **44**‐induced cytotoxicity. Compound **44** decreased the expression of the NRF2 target gene GPX4,^188^ and increased lipid peroxidation, which was diminished by ferrostatin‐1 and DFO.^270^ Increasing concentrations of **44** (10 µM) interestingly attenuated the upregulation of HO‐1.^188^ Compound **44** (1 µM) covalently binds KEAP1 at Cys151 and four other cysteines in the C‐terminal domain, thereby activating NRF2.^270^ The positioning of the epoxide at C5/C6 of ring B seems to be essential for NRF2 activation since the structurally related withanolide **69** (epoxide at C6/C7, (Figure 5) is inactive. Note that **44** affects a multitude of other signaling pathways, which makes it difficult to define functional mechanisms that contribute to ferroptosis induction.

#### 6.3.19 Cucurbitacin B (**70**)

The tetracyclic triterpenoid **70** (Figure 5) present in Cucurbitaceae and Scrophulariaceae families induces cell death in the human breast, ovarian, liver, lung, and CRC lines, being most active on human CNE1 nasopharyngeal carcinoma cells (EC_50_ = 16 nM).^271^ The ferroptosis inhibitors DFO, ferrostatin‐1, or ciclopirox neutralize the cytotoxic effects. Compound **70** evokes morphological changes that are characteristic of ferroptosis, which include shrunken mitochondria, increased mitochondrial membrane density, and a reduced number of mitochondrial cristae. Labile iron and lipid hydroperoxide levels were substantially elevated upon treatment with **70** (50 nM), whereas GSH levels as well as GPX4 and YAP protein expression (at 0.1 µM) were decreased.^271^ The latter is a proto‐oncogenic transcriptional coactivator that upregulates multiple ferroptosis regulators and promotes ferroptosis.^55^ Moreover, compound **70** caused a G2/M cell cycle arrest, most likely by disrupting microtubule dynamics, which was proposed to contribute to ferroptosis induction.^271^ Further studies are needed to elucidate the role of microtubule (**70**) as drug targets in the context of ferroptosis. Besides its ferroptotic activity, compound **70** (at 0.5 µM) also has cytoprotective functions by activating the NRF2/HO‐1 axis, as shown for cocultures of primary mouse neurons and astroglia.^272^ In a CNE1 mouse xenograft model, compound **70** (1 mg/kg, ip, 3× weekly) reduced tumor growth (sixfold) more efficiently than the first‐line drug gemcitabine (25 mg/kg, 3× weekly). The high antitumoral efficacy in vivo together with potentially low toxicity render **70** a promising ferroptosis‐inducing drug candidate and lead structure for further structural optimization.

#### 6.3.20 Glycyrrhetinic acid (**71**)

The oleanene‐type pentacyclic triterpene saponine **71** (Figure 5), a major bioactive compound isolated from *Glycyrrhiza glabra*, possesses broad antitumoral activities in different types of cancer.^273^ Remarkably, compound **71** induced ferroptotic cell death in human MDA‐MB‐231 breast cancer cells (EC_50_ = 25 µM) without substantially stimulating apoptosis or necroptosis. On the one hand, the cytotoxic effects of **71** were ascribed to an upregulation of the NOX subunit p47^phox^ and a subsequent increase in NOX activity. On the other hand, **71** impaired the expression of SLC7A11, and decreased cellular GSH levels and GPX activity (without affecting GPX4 protein expression).

Inhibitory effects on ferroptosis are also evident for **71**. In TNF‑α/*D*‐galactosamine‐challenged normal human L02 liver cells, compound **71** restored NRF2, GPX4, and HO‐1 levels, reduced lipid peroxidation, and diminished the decrease of cell viability, however only at very high concentrations (1–2 mM).^274^ To which extent ferroptosis contributes to cell death induction by TNF‑α/*D*‐galactosamine has not been addressed in this study. Despite the highly effective concentrations in vitro, compound **71 (**15–60 mg/kg, po) significantly reduced hepatic lipid peroxidation, lowered the ferrous iron content, and partially restored GSH levels as well as GPX4, NRF2, and HO‐1 expression in a mouse model of LPS/*D*‐galactosamine‑induced acute liver injury.^274^

#### 6.3.21 Oleanolic acid (**72**)

The pentacyclic triterpenoid **72** (Figure 5) from the olive tree exerts organ‐protective functions by targeting NRF2,^275^ but can also trigger ferroptosis.^276^ Thus, compound **72** reduced the viability of human HeLa cervix carcinoma cells (EC_50_ = 10–20 µM) and inhibited tumor growth, when these cells are grafted in mice (40 and 80 mg/kg, ip).^276^ Labile iron, ROS as well as MDA levels were increased and GSH levels and GPX4 expression decreased. Inhibitory effects of **72** on cell viability and proliferation were ascribed to an upregulation of ACSL4, an acyl‐CoA synthetase isoenzyme that activates PUFAs for incorporation into cellular lipids. Knockdown of ACSL4 lowered compound **72**‐induced cytotoxic effects and ROS formation and, interestingly, partially restored GPX4 protein levels.

#### 6.3.22 Corosolic acid (**73**)

The pentacyclic triterpenoid **73** (Figure 5) isolated from *Lagerstroemia speciose* has high structural similarity to **72** and is cytotoxic for various cancer cells.^277^ Compound **73** (IC_50_ = 5‐10 µM) induced ROS as well as lipid hydroperoxide formation and triggered cell death in human Caki renal carcinoma cells. Prominent cytotoxic effects were also observed in other human renal carcinoma, breast cancer and hepatocellular carcinoma cell lines but not in primary human mesangial cells, which suggests that cancer cells are more sensitive to **73** than nontransformed cells. Despite the accumulation of lipid hydroperoxides, cell death seems not to be driven by ferroptosis because DFO and ferrostatin‐1 failed to block the cytotoxic effects of **73**, which is not readily understood but might be related to the intracellular site where membrane peroxidation takes place.^278^ In support of this hypothesis, α‐tocopherol, with potentially different subcellular distribution, prevented **73**‐induced ROS formation, lipid peroxidation as well as cell death. Alternative cell death programs, such as apoptosis and necroptosis, seem not to contribute to the cytotoxic activity of **73**.

#### 6.3.23 Aridanin (**74**), ardisiacrispin B (**75**), albiziabioside A (**76**, AlbA), and its derivatives **77** and **78**

Three further oleanane‐type natural products (**74** from *Tetrapleura tetraptera*, **75** from *Ardisia kivuensis*, and **76** from *Albizia inundata*) (Figure 5) have recently been reported to raise intracellular ROS levels and induce ferroptosis.^279^, ^280^, ^281^ Compounds **74** and **75** are cytotoxic against a broad range of human breast cancer, colorectal carcinoma, glioma, lymphoblastoma, and mouse melanoma cell lines as well as several drug‐resistant phenotypes, either with P‐glycoprotein and BCRP (ABC transporters) overexpressed, p53 deleted, or EGFR or B‐RAF mutation‐activated. EC_50_ values for cell death induction were in the low micromolar range. The cytotoxic effects of the two natural products were partially decreased by the iron chelator DFO and the lipid peroxidation inhibitor ferrostatin‐1, which suggests that ferroptosis contributes to cell death induction. Compounds **75** and **74** additionally induced apoptosis and, as shown for **74**, necroptosis.^279^, ^280^

The natural oleanane triterpenoid saponin **76** exhibits selective lethality on diverse cancer cell lines as compared to nontransformed cells^282^ but the potency is moderate (16–22 µM). To obtain synergistic cytotoxic effects on cancer cells, **76** was coupled to the phosphoinositide‐dependent kinase inhibitor dichloroacetic acid (DCA).^281^ Alb‐DCA **(77)** (EC_50_ = 0.43–3.9 µM) was more active than the parental **76** and selective for cancer cells, revealing only weak cytotoxic effects on human brain microvascular endothelial cells (**76**: EC_50_ = 37.1 µM, **77**: EC_50_ = 38.2 µM) and normal human hepatic cells (LO2, **76**: EC_50_ = 33.2 µM, **77**: EC_50_ = 53.1 µM).^281^ Another approach to optimize the structure of **76** focused on esters of the carboxylic acid at C17 and yielded compound AlbA‐D13 (**78**).^282^ This derivative showed superior cytotoxicity to **76** in human colorectal, hepatocarcinoma, breast cancer, lung cancer, and gastric adenocarinoma cell lines (EC_50_ = 5–11 µM) and was sixfold more selective for cancer than nontransformed cells, that is, brain microvascular endothelial, colon epithelial and liver cells as well as keratinocytes. Compound **77**‐ and **78**‐induced cytotoxicity was reduced by DFO or ferrostatin‐1, which points toward a ferroptotic component of cell death. Moreover, compounds **78** (5 µM), **76** (10 µM), and more effectively the conjugate **77** (2 µM) elevated MDA levels and decreased GPX4 expression in human HCT116 CRCs^282^ or in human MCF‐7 breast cancer cells.^281^ Compound **77** (2 mg/kg, iv, every second day), **76** (10 mg/kg, iv, every second day), and **78** (20 mg/kg, iv, daily) significantly inhibited tumor growth in an MCF‐7 (**76** and **77**) or HCT116 (**78**), murine xenograft model, with **77** being more efficient than 5‐fluoro uracil. Notably, compound **78** significantly upregulated p53, which is required to exert cytotoxic activity. In 2016, Wei et al.^283^ described further derivatives of **76**, which selectively killed HCT116 cells (EC_50_ = 7.6 µM) and were neither cytotoxic to cancer cells from other origin nor normal cells investigated (up to 50 µM), though ferroptosis has not been addressed.

The molecular targets that underlie ferroptosis induction by the pentacyclic triterpenoids **74**, and **75** as well as **76–78** are poorly understood. Given the well‐documented NRF2‐inducing activity of **71**–**73** and the characteristic decrease of GPX4 expression by **76**–**78** together with the p53‐stimulating activity of **78**, we speculate that all members of this small molecule class share with the combined activation of the NRF2/HO‐1 and p53 a common (though not necessarily exclusive) ferroptotic mechanism. Further studies are needed to clarify whether the postulated NRF2 activation substantially contributes to ferroptosis induction by this compound class.

#### 6.3.24 Comparative discussion

**Figure 6:**
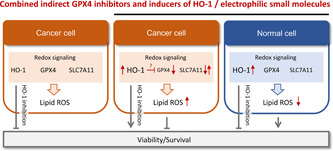
Molecular characteristics related to cancer cell‐selective lethality shared by small molecules indirectly inhibiting GPX4 and inducing HO‐1. Genetic or pharmacological inhibition of HO‐1 decreases the viability of cancer cells under basal conditions. When cancer cells are, however, exposed to electrophilic small molecules that activate NRF2 and evoke a strong expression of HO‐1, the accumulation of lipid hydroperoxides and a drop in cell survival are diminished by selective HO‐1 inhibition. Whether the often associated increase of HO‐1 and decrease of GPX4 are independent events or functionally linked is poorly understood. In nontransformed, normal cells, the NRF2/HO‐1 axis instead rather protects from lipid peroxidation and cell death. This antiferroptotic activity seems at least partially to be mediated by HO‐1. GPX4, glutathione peroxidase 4; HO‐1, heme oxygenase‐1; NRF2, nuclear factor erythroid 2‐related factor 2; ROS, reactive oxygen species. [Color figure can be viewed at wileyonlinelibrary.com]

In summary, structurally diverse small molecules trigger ferroptosis by inducing HO‐1 expression, most likely as result of NRF2 activation. At a first glance, it seems paradoxical to induce ferroptosis by activating NRF2, which upregulates adaptive pathways against oxidative and electrophilic stress, such as GSH biosynthesis. Many of the compounds in this section accordingly have cytoprotective functions in nonmalignant cells in addition to their ferroptosis‐inducing abilities, and two of them (**62, 71**) have actually been reported to prevent ferroptosis in normal cells.^247^, ^274^, ^284^ The cytotoxic activity of HO‐1‐inducing small molecules on cancer cells seems to rely on the concomitant drop in GPX4 levels, which is shared by the compounds here presented (Figure 6).

Different mechanisms seem to contribute to the drop of GPX4 levels. First, electrophilic compounds that activate the NRF2/HO‐1 axis also covalently bind to GPX4, thereby promoting proteasomal degradation, as shown for compound **41**.^181^ However, other mechanisms seem to contribute as well, since compound **44** decreased GPX4 protein levels at concentrations below those required for targeting GPX4.^188^ Second, curcuminoids (**47–49**) as well as the hybride isothiocyanates **50** and **51** downregulate GPX4 by antagonizing AR.^199^, ^202^ The important role of AR in regulating GPX4 expression has recently been confirmed by AR overexpression in HEK‐293 cells and AR knockdown in ferroptosis‐resistant triple‐negative LAR breast cancer cells, and this counter‐regulation correlates with ferroptosis sensitivity.^203^ Third, GPX4 levels are suppressed likely following p53 activation, as suggested for tanshinones (**62**–**64)** and compounds **78** and **92**.^243^, ^282^, ^285^ Compound **92** is described in Section 8 because the evidence is missing that the HO‐1/NRF2 axis is targeted.

Small molecules that lower GPX4 and induce HO‐1 expression gain antitumoral activity when combined with inhibitors of GSH biosynthesis or regeneration. In support of HO‐1 promoting ferroptosis, its inhibition by ZnPP protected human HT‐1080 fibrosarcoma cells from the system X_c_^−^ inhibitor **18**.^193^ Silencing of HO‐1 instead enhanced **18**‐induced cytotoxicity in human HepG2 cancer cells, which rather points toward antiferroptotic properties of HO‐1.^43^ Increasing evidence suggests that HO‐1 expression offers selective cytotoxicity against cancer cells over nontransformed cells. The latter even seem to benefit from abundant HO‐1 expression in terms of survival. On the other hand, moderate HO‐1 expression also sustains cancer cell survival by counteracting apoptosis and stimulating angiogenesis,^286^ and HO‐1 inhibition has been suggested as a promising strategy in anticancer therapy.^287^ Taken together, activators of the NRF2/HO‐1 axis that additionally reduce GPX4 expression have promising cancer cell selectivity and, when given as combination therapy with conventional drugs, might even protect normal cells. However, their cytotoxic effects seem to depend on the genetic background and metabolic state of the cells as well as the extent and potential duration of HO‐1 upregulation.^288^ The bivalent role of HO‐1 on ferroptosis is not fully understood but seems to be tightly associated with the iron state of cancer cells.^289^ We speculate that the higher iron status of cancer cells compared to normal cells as well as their dependency on GPX4‐protective mechanisms against lipid peroxidation contribute to the selective toxicity of combined GPX4‐suppressing and HO‐1‐inducing compounds toward malignant cells. Future studies will be required to explore the potential and safety of drug candidates that simultaneously target GPX4 and the NRF2/HO‐1 axis in cancer patients.

A drop in GPX4 levels is expected to be more challenging for cancer cells than for normal cells because of their high metabolic turnover that is associated with oxidative stress. Nonetheless, many covalent GPX4 inhibitors are toxic to both cancer and normal cells, potentially because they are confronted by poor selectivity. Preferentially decreasing GPX4 protein levels might therefore be a more proficient and safer strategy to induce ferroptosis than direct inhibition of GPX4 through covalent binding.^2^ The exact mechanisms that deplete cells from GPX4 are only partially understood. Distinct ferroptosis inducers (**45** and **46**) indirectly inhibit GPX4 by reducing the enzyme's cellular availability, for example, by stimulating nonproteasomal degradation. On the other hand, the curcuminoids **47–49** and the hybrid AR antagonists **50** and **51** likely decrease GPX4 levels by interfering with AR signaling.

Recent evidence suggests that GPX4 inhibition may also affect cells of the immune system and negatively impact antitumor immunity.^290^ Two immune‐suppressive strategies have been proposed to limit or even outweigh the anticancer activity of the direct GPX4 inhibitor **23** in vivo. On the one hand, compound **23** kills tumor‐infiltrating CD8^+^ T cells (besides cancer cells) that are important for the antitumor immune response. On the other hand, ferroptotic cell death fosters the release of immune‐suppressive factors (including prostaglandin E_2_) by pathophysiologically activated neutrophils, which further dampens the immune response. Future studies are needed to clarify whether these negative effects are (i) related to direct inhibition of GPX4, (ii) might be circumvented by compounds with superior cancer cell selectivity, and (iii) are limited to distinct cancer entities. These new insights into cancer–immune interactions also offer access to new therapeutic strategies. Silver‐coated NRF2‐targeting ZVI‐nano particles (ZVI@Ag), for example, induce ferroptosis in human A549 lung cancer cells while simultaneously increasing antitumor immunity in immune‐competent mice. Mechanistically, ZVI@Ag (i) reduces the number of regulatory T cells, a subpopulation of CD4^+^ cells with immunosuppressive properties, (ii) enhances the cytolytic activity of CD8^+^ cells by downregulating the negative immune checkpoints PD‐1 and cytotoxic T‐lymphocyte‐associated protein 4, and (iii) reprograms protumor M2 to antitumor M1 macrophages.^291^ The biogenic ferroptosis inducer **102** also appears to enhance anticancer immunity by increasing the populations of CD8^+^ T cells, natural killer cells, and natural killer T cells.^292^ Altogether, these findings suggest that the detrimental immune‐suppressive effects of **23** are related to direct GPX4 inhibition rather than being a general feature of ferroptosis‐inducing small molecules. Critical issues that need to be tackled are the in vivo efficacy of ferroptosis‐inducing compounds in immunocompetent model systems along with the ferroptosis susceptibility of different types of immune cells.

## 7 ACTIVATORS OF THE NRF2/HO‐1 AXIS

This chapter summarizes small molecule NRF2 activators, which do not fall into the categories described in Section 6.3, either because they do not additionally repress GPX4 expression or because the experimental proof is (still) lacking.

### 7.1 Bay 11‐7085 (**79**)

The 2‐sulfonyl‐acrylnitril derivative **79** (Figure 5) inhibits IκBα and has antiproliferative and proapoptotic activity in various cancer cell lines.^204^ The cytotoxic effects of **79** on human MDA‐MB‐231 breast cancer cells are independent of NF‐κB signaling and related to ferroptosis induction (EC_50_ = 5 µM). As suggested from HO‐1 inhibition by ZnPP, compound **79** activates NRF2 and triggers ferroptosis through the markedly upregulated NRF2 target gene HO‐1. Compound **79** further increased SLC7A11 protein expression, as expected from NRF2 activation, but did not elevate the expression of GPX4 (within 8 h). Inhibition of system X_c_^−^ by **18** enhanced the cytotoxic effect of **79**, likely by compensating for the upregulation of SLC7A11.

### 7.2 Piperlongumine (**80**)

**Figure 7:**
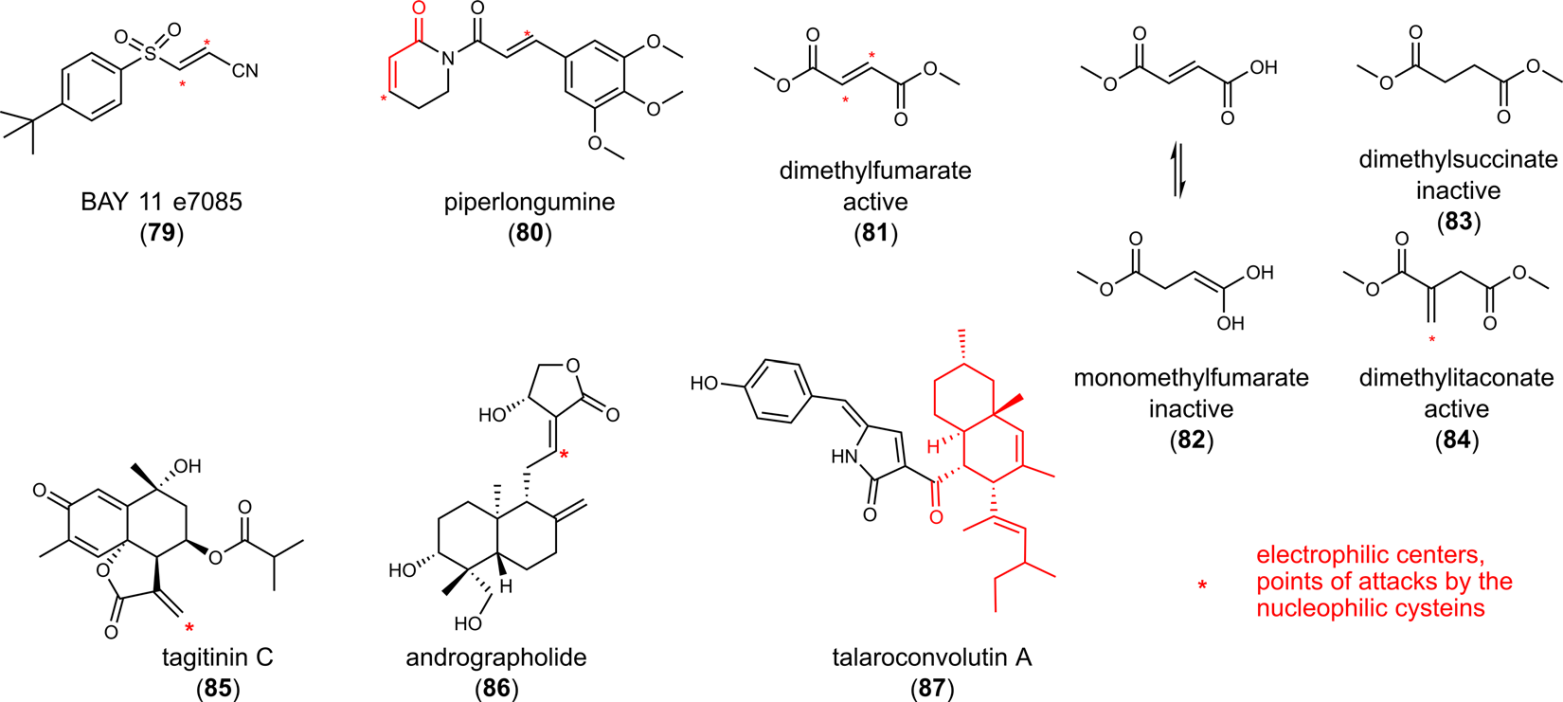
Inducers of the nuclear factor erythroid 2‐related factor 2/heme oxygenase‐1 axis [Color figure can be viewed at wileyonlinelibrary.com]

The alkaloid **80** (Figure 7; 5–10 µM) isolated from *Piper longum* induced cell death in pancreatic cancer and breast cancer cells but hardly affected normal human MCF‐10A breast epithelial cells.^293^, ^294^ Compound **80** has two electrophilic α,β‐unsaturated carbonyl functions, directly bind to KEAP1 and activate NRF2. The selective cytotoxicity against cancer cells has been ascribed to an upregulation of HO‐1 protein expression via the NRF2 axis.^295^ Compound **80** upregulated HO‐1 both in human MCF‐7 breast cancer cells as well as nontransformed MCF‐10A cells, but with different consequences on cell death. Thus, pharmacological inhibition or knockdown of HO‐1 decreased the cytotoxic effect of **80** in MCF‐7 cells but enhanced cell death induction in MCF‐10A cells. These findings suggest that HO‐1 expression is protective in normal cells but death promoting in cancer cells, which might provide an explanation for the cancer cell‐selective cytotoxicity of **80** and other NRF2‐activating compounds.^295^ Lipophilic radical traps (ferrostatin‐1, liproxstatin‐1) or iron chelators (DFO, ciclopirox) but not apoptosis (Z‐VAD‐FMK) or necroptosis inhibitors (necrostatin‐1) impaired the cytotoxic activity of **80** on human PANC‑1 pancreatic cancer cells.^293^ While this profile points toward a ferroptotic mechanism, it should be noted that also the water‐soluble antioxidant *N*‐acetyl cysteine blocked cell death induction by **80**. The increase of intracellular ROS levels upon treatment with **80** was ascribed to the direct inhibition of glutathione *S*‐transferase and the decrease of intracellular GSH levels. Another study reported that **80** binds to (*K*_d_ = 22 µM) and inhibits thioredoxin reductase 1 (IC_50_ = 5–10 µM) and induces ROS‐mediated apoptosis in human GC cells (EC_50_ = 10–15 µM).^296^ The NRF2 target gene thioredoxin reductase 1 is a flavoprotein that uses NADPH to regenerate thioredoxin and is implicated in the protection from ferroptosis.^185^, ^297^ Docking studies suggest a covalent coupling of **80** to Cys498 in the redox‐active center of the enzyme. As observed for other ROS‐inducing natural products, **80** also induces other forms of cell death besides ferroptosis, including apoptosis, autophagy, and necrosis, seemingly dependent on the cell type/line and experimental condition.^298^, ^299^

### 7.3 Dimethyl fumarate (**81**, brand name Tecifidera)

The dimethyl ester of fumarate **81** (Figure 5), an intermediate of the citric acid cycle, was initially approved for the treatment of psoriasis and in this course randomly discovered to ameliorate symptoms of multiple sclerosis.^137^ Compound **81** is in clinical use in multiple sclerosis and under clinical investigation (single and combination therapy) for the treatment of various types of cancer, for example, refractory chronic lymphocytic leukemia, small lymphocytic lymphoma, cutaneous T cell lymphoma and glioblastoma (www.clinicaltrial.gov, NCT02784834, NCT02337426, NCT02337426, NCT02546440).

The impact of **81** on ferroptosis is ambiguous.^300^ On the one hand, compound **81** (20 µM) inhibited ferroptosis in nontumorous damaged or stressed cells, along with increasing GSH levels and decreasing lipid peroxidation.^284^ Compound **81** also elevated GSH levels in mouse MLE‐12 lung epithelial cells undergoing seawater‐induced ferroptosis, which was ascribed to an activation of the Nrf2 pathway.^284^ On the other hand, the germinal center B‐cell‐like (GCB) subtype of the diffuse large B‐cell lymphoma (DLBCL) cell line has low GSH and GPX4 levels and strongly expresses 5‐LOX, which renders these subtype susceptible to ferroptotic cell death by **81** (20 µM).^301^ The activated B‐cell‐like (ABC) subtype is also vulnerable to **81** (20 µM), but cell death does not involve ferroptosis and depends on the inhibition of NF‐κB and Janus kinase (JAK)/signal transducer and activator of transcription 3 signaling.

The Michael acceptor **81** influences cell signaling through its reactivity toward nucleophiles. The less reactive monomethyl fumarate (**82**) and the nonelectrophilic dimethyl succinate (**83**) accordingly failed to impair DLBCL cell proliferation, whereas the structurally related Michael acceptor dimethyl itaconate (**84**) was comparably active (Figure 5).^301^ Chemical proteomic mapping identified 40 cysteine residues among 2400 cysteines of the cysteinome that are modified by **81**.^302^ Succination of IκB kinase at Cys179 and JAK at the highly conserved Cys257 explains the suppression of NF‐κB and JAK survival signaling by **81**, and depletion of GSH by covalent coupling with **81** accounts for the induction of ferroptosis in the GCB subtype of lymphoma cells. Interestingly, compound **81** (25 µM) enhanced the mRNA expression of the NRF2 target gene HO‐1 in human primary peripheral blood mononuclear cells,^303^ and we hypothesize that this mechanism contributes to the ferroptosis‐inducing activity and cancer cell selectivity of **81**. However, other mechanisms contribute to the cytotoxicity of **81** as well, and they are not necessarily limited to ferroptosis. For example, compound **81** induced cell death in human colorectal HCT116 and SW480 cancer cells (at 25–100 µM) through a nonferroptotic pathway dependent on HIF‐2α,^304^ which underlines the importance of the cellular origin, (epi)genetic background, and/or metabolic state in defining the antitumoral mechanism and effectiveness of (electrophilic) small molecules. When administered to mice with xenografted HCT116 and SW480 cells, compound **81** (10 mg/kg, ip) sensitized the tumor to a hypoxic mimetic but failed to reduce tumor weight on its own.^304^

### 7.4 Tagitinin C (**85)**

The heliangolide‐type sesquiterpene lactone **85** (Figure 7) is commonly found in the Asteraceae family of plants and known for its antitumor, antiparasitic, antiviral, antifibrotic, and cardioprotective properties.^305^ Compound **85** triggered cell death in human CRCs (EC_50_ = 15 µM) and showed synergistic cytotoxicity when combined with the X_c_^−^ inhibitor **18**.^306^ Cell death was attenuated by DFO and ferrostatin‐1 within 12 h but neither by inhibitors of apoptosis (Z‐VAD‐FMK), autophagy (3‐MA), nor necroptosis (necrostatin‐1). Compound **85** rapidly induced lipid peroxidation and reduced GSH levels, which was suggested to be mediated by protein kinase R‐like ER kinase (PERK)‐dependent NRF2 activation. Consequently, the NRF2 target genes HO‐1, SLC7A11, NQO1, FTH1, GCLC, and GCLM were upregulated, while GPX4 mRNA levels did not substantially change. Of note, DFO and ferrostatin‐1 failed to prevent compound **85‐**induced cell death at 24 or 48 h of incubation, which indicates that other forms of cell death dominate the cytotoxic activity at later time points.

### 7.5 Andrographis extract standardized to 20% andrographolide (**86**)

Protective effects of the diterpenoid andrographolide (Figure 7), which is a major bioactive component of andrographis extracts, have been described in various models of organ injuries in vitro (5–25 µM)^307^ and in vivo (20 mg/kg sc, 10 mg/kg ip, 30–120 mg/kg po).^308^ On the other hand, *Andrographis paniculata* extracts were cytotoxic on human HCT116 and SW480 CRCs (EC_50_ = 10 and 20 µg/ml, respectively) and sensitized cell lines with acquired resistance to 5‐fluoro uracil.^309^ Cell death triggered by the andrographis extract was ascribed to a caspase 9‐dependent induction of apoptosis and the initiation of ferroptosis via the NRF2/HO‐1 axis. The relative contribution of these cell death programs to the overall loss in cell viability is still elusive. The andrographis extract (125 mg/kg, ip, every second day) effectively reduced tumor weight in a xenograft mouse model with 5‐fluoro uracil‐resistant HCT116 cells (fourfold). Coadministration of the extract and 5‐fluoro uracil (30 mg/kg, ip) was only marginally more efficient. Another study showed that compound **86** (25–100 mg/kg, ig administration) protects from 5‐fluoro uracil‐induced intestinal mucositis in tumor‐bearing mice with grafted H22 hepatocellular carcinoma cells.^310^ These findings are in line with the dual role of NRF2/HO‐1‐inducing small molecules in anticancer treatment.^48^, ^191^ On the one hand, NRF2/HO‐1 activators seem to synergize with conventional chemotherapeutics to reduce cancer cell survival. On the other hand, they beneficially reduce the cytotoxicity of anticancer drugs in (distinct) nontransformed cells.

### 7.6 Talaroconvolutin A (**87**)

The azaphilone **87** (Figure 7) was isolated from the endophytic fungus *Talaromyces purpureogenus* inhabiting *Panax notoginseng*.^311^ Compound **87** efficiently triggered cell death in various CRC cell lines (human HCT116, SW480, and SW620; EC_50_ = 8–12 µM) without stimulating apoptosis. ROS levels increased and morphological changes characteristic for ferroptosis were induced, which includes membrane perforations and ruptures, shrunken mitochondria and a decline in the number of mitochondrial cristae.^311^ Cytotoxic effects of **87** were attenuated by the ferroptosis inhibitor ferrostatin‐1 and the iron chelator deferiprone. Compound **87** enhanced lipid peroxidation and changed the expression of proteins that are tightly associated with ferroptosis (e.g., FTL, SLC7A11, HO‐1, epidermis‐type lipoxygenase 3, GSS, ACSL5). Effects on NRF2 were not investigated but the expression profile strongly suggests that **87** interferes with the NRF2 axis. The authors ascribed the ferroptotic activity of **87** to the downregulation of SLC7A11 (a component of system X_c_^−^) and upregulation of epidermis‐type lipoxygenase 3 (a lipoxygenase isoenzyme i.e. highly expressed in epidermis), as confirmed by knockdown and overexpression studies. Moreover, compound **87** (6 mg/kg, ip, every other day) decreased tumor weight by 30% in a murine xenograft model using human HCT116 cells.

## 8 NRF2 INHIBITORS

**Figure 8:**
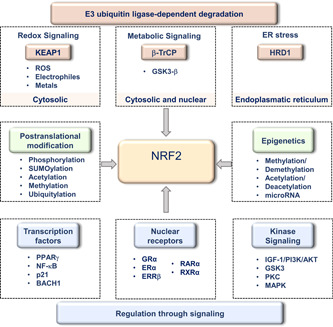
NRF2‐regulatory pathways. The transcription factor NRF2 consists of multiple domains that are under tight regulatory control. In complexes with KEAP1, NRF2 is ubiquitinated and degraded by the proteasome. Electrophilic and oxidative modifications of cysteine residues in KEAP1 lower the affinity to NRF2, and factors like p21 or NF‐κB hamper the binding of KEAP1 to NRF2. Ubiquitin‐dependent NRF2 degradation is further determined through the availability of the substrate recognition component of the SKP1‐cullin 1‐F‐box protein E3 ligase complex β‐TrCP, which responds to metabolic changes and is regulated by GSK3‐β. Another NRF2‐regulatory factor is the E3 ubiquitin‐protein ligase HRD1 which participates in ER‐associated degradation during ER stress. A variety of signaling cascades regulate the expression, nuclear availability, DNA‐binding affinity, and transactivation activity of NRF2, and also posttranslational and epigenetic modifications essentially contribute to the regulation of NRF2 activity. These pathways and factors represent potential targets for NRF2‐inhibiting small molecules. AKT, protein kinase B; BACH1, BTB domain and CNC homolog 1; ERRβ, estrogen‐related receptor β; ERα, estrogen receptor α; GRα, glucocorticoid receptor α; GSK3‐β, glycogen synthase kinase‐3β; HRD1, HMG‐CoA reductase degradation protein 1; IGF‐1, insulin‐like growth factor 1; MAPK, mitogen‐activated protein kinase; NF‐κB, nuclear factor κ‐light‐chain‐enhancer of activated B cells; NRF2, nuclear factor erythroid 2‐related factor 2; PI3K, phosphatidylinositol 3‐kinase; PKC, protein kinase C, PPARγ, peroxisome proliferator‐activated receptor γ; RARα, retinoic acid receptor α; RXRα, retinoid X receptor α. [Color figure can be viewed at wileyonlinelibrary.com]

Manifold signaling pathways regulate NRF2 activation,^42^ as exemplarily outlined in Figure 8. Of particular importance are the E3 ubiquitin ligase complexes CUL3‐RBX1‐KEAP1, SCF/β‐TrCP, and HRD1, which essentially control the availability of NRF2 by marking the transcription factor for ubiquitin‐dependent degradation.^312^ Binding of specific factors to either KEAP1 or NRF2 interrupts the interaction of the two proteins, which prevents NRF2 from being degraded.^42^ The CDK inhibitor p21 for example, competes with KEAP1 for the binding to NRF2, whereas the phosphorylated autophagy substrate p62 binds to KEAP1 and disrupts the interaction with NRF2.^42^ NRF2 transcription is in addition enhanced by diverse oncogenic and stress‐activated pathways (e.g., via K‐RAS, B‐RAF, phosphatidylinositol 3‐kinase/protein kinase B, extracellular signal‐regulated kinase, p38 MAPK), the GRP78‐PERK branch of the unfolded protein response as well as the energy sensor AMPK.^132^ Not only the expression but also the activity of the transcription factor NRF2 is also under tight control. NRF2 is regulated by (i) the import into and export from the nucleus, (ii) phosphorylation, (iii) transactivation by dimerization with different transcription factors or nuclear receptors, and (iv) the affinity to antioxidant response elements (ARE), the DNA binding sites for NRF2.^42^

The heterogeneity in the mechanisms that regulate NRF2 signaling is reflected by the diversity of small molecules that interfere with NRF2 activation.^48^, ^132^ For a comprehensive overview of natural products that target NRF2, we want to refer to recent review articles.^46^, ^48^, ^132^, ^312^, ^313^, ^314^, ^315^, ^316^, ^317^, ^318^, ^319^, ^320^ Here, we place focus on those NRF2 inhibitors, which explicitly trigger ferroptotic cell death, and on ferroptosis‐stimulating compounds, which we anticipate to inhibit NRF2 based on their effect on NRF2 target gene expression. Ferroptosis‐inducing natural products, for which inhibition of NRF2 is less obvious, are instead listed in Section 9.

### 8.1 ML385 (**88**)

**Figure 9:**
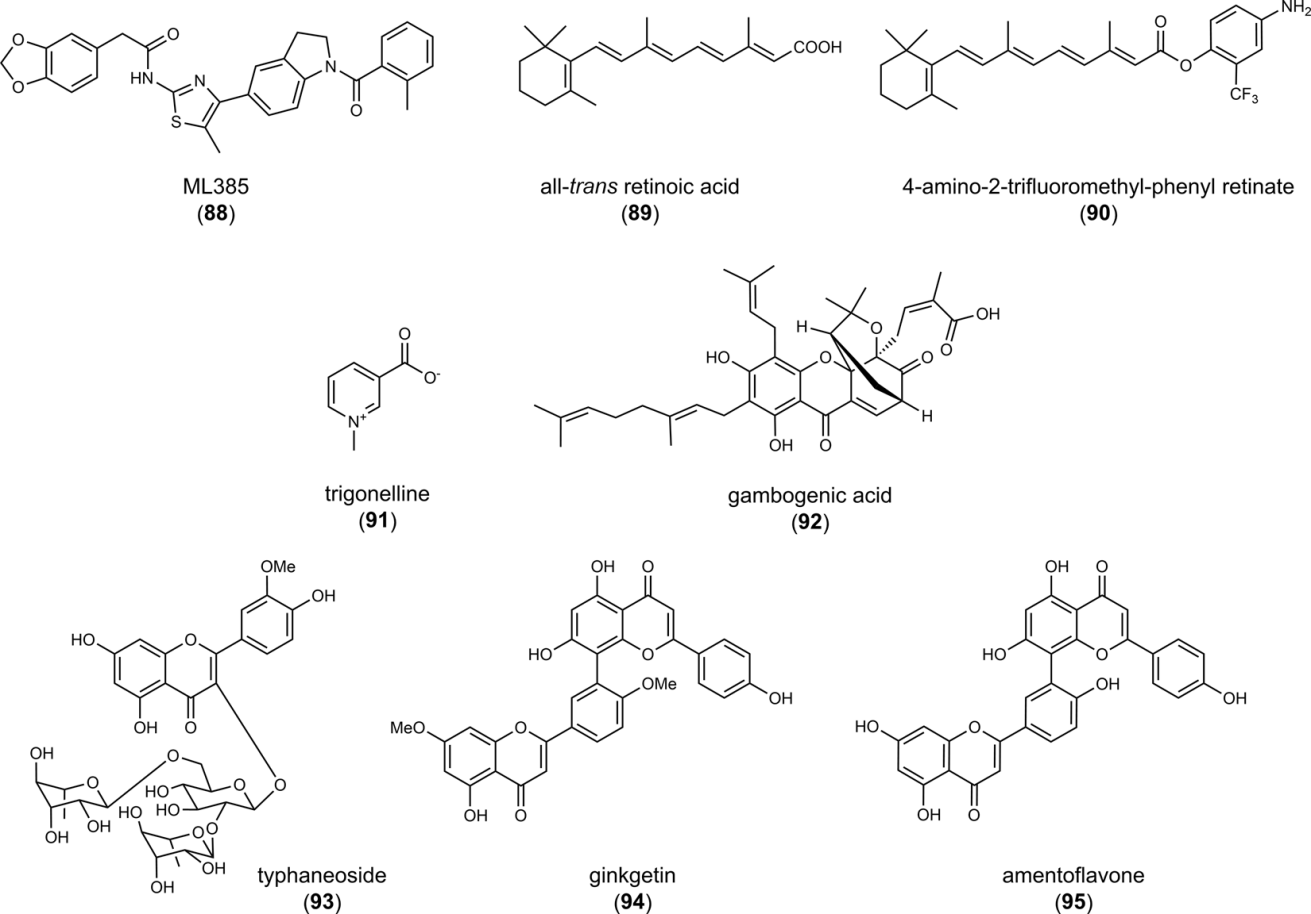
Nuclear factor erythroid 2‐related factor 2 inhibitors

In a large‐scale screen with 400,000 compounds, **88** (Figure 9) was recently discovered as an inhibitor of NRF2.^321^ Compound **88** enhanced seawater exposure‐induced ferroptosis (at 20 µM), increased MDA and lipid hydroperoxide formation and decreased GSH levels as well as Gpx4 mRNA expression in MLE‐12 mouse lung epithelial cells.^284^ Mechanistically, compound **88** directly binds to the Neh1 DNA binding domain of NRF2 and disrupts the binding of the NRF2–V‐Maf avian musculoaponeurotic fibrosarcoma oncogene homologue complex to the ARE sequence within the NRF2 target gene promoters.^321^ Note that **88** is the only known direct NRF2 inhibitor. Other compounds indirectly suppress NRF2 signal transduction by targeting upstream pathways or inducing proteasomal degradation (Figure 8).

The strong impact of KEAP1 mutations on the cellular susceptibility to **88** supports that NRF2 inhibition is causative for ferroptosis induction. Thus, compound **88** more effectively suppressed colony formation in human H460 lung carcinoma cells with inactivating KEAP1^D262H^ mutation and high NRF2 activity (GC_50_ = 1.5 µM) than in the wild‐type counterpart (H460 cells with knock‐in of wild‐type KEAP1; GC_50_ = 8–16 µM). Moreover, **88** inhibited the expression of the NRF2‐target gene NQO1 in lung cancer cells (by 55% at 5 µM)^321^ and showed pronounced cytotoxic activity (at 10 µM) in a cell line with inactivating KEAP1^G333C^ mutation. Growth‐inhibitory effects on nontumorigenic lung epithelial cells (BEAS2B) were instead not evident up to a concentration of 25 µM.

In therapy‐resistant NSCLC with KEAP1 deficiency, **88** (5 µM) substantially enhanced the cytotoxicity of doxorubicin and taxol.^321^ When combined with the antitumoral drugs paclitaxel, doxorubicin, or carboplatin, **88** synergistically attenuated colony formation of human A549 and H460 lung cancer cells and induced caspase activation. This finding suggests that **88**, which does not induce apoptosis per se, triggers supportive mechanisms, potentially at the crosslink between ferroptosis and apoptosis, where also NRF2 is located.

Compound **88** (30 mg/kg, ip daily) suppressed tumor growth in xenografts of A549 and H460 cells in mice comparably to carboplatin (5 mg/kg, ip), and the combination of the two cytotoxic agents showed synergistic effects. The latter was ascribed to an accumulation of carboplatin in the tumor, most likely as a result of the impaired NRF2‐dependent upregulation of ABCG2 (BCRP) and potentially other efflux transporters.^321^, ^322^

### 8.2 All‐*trans* retinoic acid (**89**) and the derivative 4‐amino‐2‐trifluoromethyl‐phenyl retinate (**90**)

Both compounds **89** and **90** (10 µM, Figure 9) downregulated NRF2 protein levels (**89**: by 30% at 10 µM, **90**: by 50% at 10 µM) and induced cell death (EC_50_ = 1–10 µM) in human NB4 acute myeloid leukemia (AML) cells.^323^ The cytotoxic effect of **90** was associated with an increase in lipid hydroperoxide and MDA levels (0.1–10 µM), a depletion of the GSH pool, and a reduced expression of NRF2 target genes, such as GPX4, ferritin, and SOD1, which was reversed by ferrostatin‐1.^323^ Effects of **89** on ferroptosis markers were not investigated. In a xenograft mouse model using NB4 cells, compound **90** (10 mg/kg, ip) efficiently decreased GPX4 and elevated COX‐2 expression in the tumor. In addition, NRF2 forms a complex with the retinoic acid receptor in the presence of **89** (Figure 9), which disables the binding to the ARE sequence and thereby disrupts NRF2 signaling,^42^ a mechanism that likely also applies to **90**. Together, compounds **89** and **90** inhibit NRF2‐dependent gene expression and, as shown for **90**, induce ferroptosis.^323^

### 8.3 Trigonelline (**91**)

The coffee alkaloid **91** (Figure 9) is a metabolite of niacin and present in many plants including *Coffea species* (Rubiaceae) and *Trigonella foenum‐graecum* (Fabaceae).^324^ Compound **91** decreased nuclear NRF2 protein levels in the human pancreatic carcinoma cell lines Panc1, Colo357, H6c7, and MiaPaca2 as well as NRF2 activity in a reporter gene assay at submicromolar concentrations (0.1–1 µM), which was ascribed to an impaired import of NRF2 into the nucleus.^324^ Neither nuclear NRF2 export nor total cellular NRF2 protein levels were influenced by **91**. By inhibiting NRF2, **91** sensitized pancreatic cancer cell lines as well as grafted tumors in mice to apoptosis upon cotreatment with the topoisomerase II inhibitor etoposide. The potential of **91** (150–300 µM) to overcome ferroptosis‐resistant mechanisms in cancer was confirmed in human head and neck cancer cell lines.^325^ Compound **91** markedly enhanced their sensitivity to the GPX4 inhibitor **23**, further increased lipid peroxidation by interfering with NRF2 signaling, and decreased tumor growth in a mouse xenograft model.^325^ Together, combination therapy of conventional anticancer drugs and **91** promises superior efficacy in the treatment of resistant tumors.

### 8.4 Gambogenic acid (**92**)

The bioactive natural product **92** (Figure 9) isolated from the dry resin of *Garcinia hanburyi* (Gamboge) showed marked toxicity in human A375 (EC_50_ = 1.5 µM) and A2058 (EC_50_ = 0.9 µM) melanoma cells that undergo transforming growth factor(TGF)‐β‐induced EMT. Nonstimulated cells with lower metastatic capacity (without TGF‐β treatment) were instead less sensitive (A375: EC_50_ = 4 µM; A2058: EC_50_ = 1.5 µM).^285^ EMT is a dedifferentiation process of epithelial cells, in which they lose their polarity and obtain features of mesenchymal stem cells, such as the capacity to differentiate into multiple tissue lineages. Cells undergoing EMT are characterized by decreased cell–cell contacts, an elevated migratory and invasive potential, and acquired resistance against cell death by apoptosis.^326^ On the other hand, cancer cells with mesenchymal features are more susceptible to ferroptosis,^87^, ^327^, ^328^ most likely due to an upregulation of enzymes involved in the activation and membrane incorporation of long‐chain PUFAs.^87^, ^329^

Cell death induced by compound **92** is inhibited by the ferroptosis inhibitor ferrostatin‐1, the iron‐chelating agent DFO as well as an autophagy inhibitor but not by an inhibitor of apoptosis.^285^ The morphology and microstructure of mitochondria accordingly show hallmarks of ferroptosis (i.e., smaller mitochondria and an increase in membrane density) as well as autophagy (i.e., presence of autophagy bodies). Compound **92** increased membrane peroxidation (levels of membrane hydroperoxides and the lipid peroxidation end product MDA) and decreased GPX4 and SLC7A11 protein expression, which was ascribed to an upregulation of p53.^285^ Moreover, GSH levels were decreased, as was the activity of SOD. These findings strongly suggest an efficient suppression of NRF2 signaling by **92** and hint toward a putative role of p53 in mediating this effect.

### 8.5 Typhaneoside (**93**)

The bioactive component **93** (Figure 9; 40 µM), isolated from pollen of *Typha angustata*, triggered cell death in AML cells (Kas‐1, HL60, and NB4).^330^ The loss of cell viability was accompanied by elevated lipid hydroperoxidation, depleted GSH levels, and reduced GPX4 mRNA expression. Cotreatment with ferrostatin‐1 or DFO diminished the cytotoxic effect. While this profile is in line with NRF2 inhibition, experimental proof is lacking, and the cytotoxic mechanism of **93** remains speculative. Despite a relatively low potency in vitro, Compound **93** showed favorable in vivo activity in an HL60 cell BALB/c xenograft mouse model (10–40 mg/kg, ip, once daily). However, the role of ferroptosis in the overall cytotoxicity of **93** is still diffuse, and other forms of cell death, i.e., autophagy and apoptosis, participate as well.

### 8.6 Ginkgetin (**94**)

The biflavonoid **94** (Figure 9) from *Ginkgo biloba* synergizes with the anticancer drug cisplatin in the treatment of therapy‐resistant NSCLC. Compound **94** induced autophagic, apoptotic and to a limited extent ferroptotic cell death, and enhanced cytotoxicity by cisplatin through a ferroptotic mechanism.^331^ Cancer cells seem to be more sensitive to compound **94**‐induced cell death than nontransformed cells. Thus, compound **94** suppressed more potently the viability of human A549 epithelial lung cancer (EC_50_ = 5 µM) than of human nontumorigenic BEAS‐2B epithelial lung cells (EC_50_ = 150 µM), whereas cisplatin was equally effective on both cell lines (EC_50_ = 7.5 µM). The cytotoxicity of **94** was reduced by GPX4 overexpression and marginally by the ferroptosis inhibitors UAMC‐3203 (a derivative of ferrostatin‐1) and DFO. Effects were more pronounced for cotreatment with **94** and cisplatin. Lou et al. proposed that the cell death programs are hierarchically linked under these conditions, with ferroptosis promoting apoptosis, as indicated by a loss in mitochondrial membrane potential, serine externalization, and the activation of initiator and effector caspases.^331^ Compound **94** (5 µM) elevated the labile iron pool, ROS formation, and lipid peroxidation, while decreasing GPX4 and HO‐1 expression as well as the cellular GSH to GSSG ratio. The role of NRF2 in this process has been challenged because NRF2 activators, either sulforaphane (**52**) or dimethyl fumarate (**81**), did not diminish **94**‐induced cytotoxicity. On the other hand, cisplatin (5 µM) increased ARE‐mediated NRF2 transcriptional activity (2.6‐fold), and cotreatment with **94** abolished this effect. In an NSCLC xenograft mouse model using human A549 lung cancer cells, a single treatment with either **94** (30 mg/kg, ip, once daily) or cisplatin (3 mg/kg, ip, 2–3 times per week) reduced tumor weight by 50%. Cotreatment of cisplatin and **94** was considerably more effective (80% reduction of tumor weight), which underlines the potential of combining conventional anticancer drugs with proferroptotic agents in the treatment of NSCLC.

### 8.7 Amentoflavone (**95**)

The bioactive biflavonoid **95** (Figure 9) from *Selaginalla species* stimulated cell death in human U251 and U373 glioma cells (at 10–20 µM) and, when given ip (40–80 mg/kg), effectively reduced the size of U251 xenograft tumors in mice (by 50%–75%), while not affecting the viability of primary human astrocytes in vitro.^332^ DFO or ferrostatin‐1 is protected from cytotoxic effects by amentoflavone, which points toward a ferroptotic mechanism. The authors suggest that the induction of ferroptosis is related to a decreased expression of FTH, which evokes changes in iron homeostasis that are associated with an mammalian target of rapamycin (mTOR)/AMPK/p70SK‐dependent induction of autophagy. Compound **95** (20 µM) further increased iron, MDA and lipid hydroperoxides and decreased cellular GSH levels. Whether **95** acts via the NRF2 axis was not investigated. However, we consider such a mechanism likely due to structural similarities of **95** and **94** (Figure 9). The two compounds share the same dimeric scaffold and only differ in the methylation of polyphenolic hydroxyl groups.

### 8.8 Comparative discussion

In summary, an emerging number of studies demonstrate a beneficial role of NRF2 inhibitors in drug‐resistant cancer therapy, both for cultured cell lines in vitro and grafted cancer cells in vivo. Except for **88**, all inhibitors derive from natural sources or are natural product‐inspired compounds. Another synthetic NRF2 inhibitor, AEM1, was recently identified in a large‐scale screen,^333^ but neither was the target/binding site of AEM1 identified nor the effect on ferroptosis investigated. The exact mechanisms of how natural products interfere with the NRF2 axis often remain obscure, and heterogeneous indirect mechanisms are likely involved. Several natural products like brusatol—a well‐known natural NRF2 inhibitor—and **91** have nonselective anticancer activity and inhibit NRF2 signaling both in normal and tumor‐derived cells.^333^ Other natural products like **94** instead exhibit selectivity toward cancer cells as compared to normal cells, thereby being superior to the synthetic drug candidate **88**. However, it should be taken into account that comparisons in the selectivity for cancer cells over nontransformed cells can only be crude estimates when based on different experimental systems with varying susceptibility to ferroptosis. Our overall impression is that compounds more specifically interfering with NRF2 signaling may have advantages in inducing selective cancer cell lethality. The mechanisms are not fully understood but likely involve a reduced capacity to adapt to redox stress and detoxify xenobiotics (e.g., conventional anticancer drugs), which is important for tumor resistance.^48^ In fact, a broad spectrum of cancer cells from different origin rely on an activated NRF2 axis.^138^ Considering that many of the here described small molecules have multiple targets, detailed mechanistic studies are urgently needed to estimate the relevance of NRF2 inhibition for their ferroptosis‐inducing and/or antitumoral activity.

## 9 FERROPTOSIS‐INDUCING NATURAL PRODUCTS WITH DIVERSE MECHANISMS

Phospholipid and neutral lipid hydroperoxides accumulate during ferroptosis and are degraded to metabolites with truncated fatty acid chains and reactive aldehydes that may disrupt the membrane architecture.^101^ Polyunsaturated acyl chains are oxidized by nonenzymatic mechanisms, LOX, or dependent on cytochrome P450 oxidoreductases to lipid hydroperoxides.^36^, ^96^, ^334^ Defense systems against membrane peroxidation involve GPX4 that reduces peroxidized fatty acids to nonreactive alcohols using GSH as cofactor^335^ and radical‐trapping antioxidants, such as CoQ_10_.^82^, ^336^ Reported small molecules that induce ferroptosis either inhibit GPX4, deplete GSH, oxidize iron,^32^, ^336^, ^337^ or incorporate PUFAs into phospholipids or neutral lipids.^101^ In Sections 5 and 6, we discuss system X_c_^−^ and GPX4 inhibitors that directly interfere with lipid hydroperoxide detoxification via GPX4 or indirectly by depleting intracellular GSH pools. Section 6.3 highlights potential NRF2 activators that exert their ferroptosis‐inducing activity by inducing HO‐1 expression and lowering GPX4 levels along with engaging multiple other mechanisms, such as an interference with GSH biosynthesis or iron metabolism. Additional small molecules that activate or inhibit NRF2 but do not fit into these categories (either because they do not target system X_c_^−^/GPX4 or experimental proof is lacking) are summarized in Sections 7 and 8. The following section concentrates on natural products and derivatives, which induce ferroptosis, either by oxidizing ferrous iron, interfering with the antioxidative defense system, or through other known or unknown mechanisms. The latter is a heterogeneous group that requires more detailed investigation and may include, among others, GPX4, system X_c_^−^ and NRF2 inhibitors as well as iron‐oxidizing compounds or modulators of the membrane fatty acid composition. Sensitizing to ferroptosis was not a sufficient criterion for being included in this chapter if the natural product does not possess marked cytotoxic activity itself at micromolar concentrations. Included are only natural products that have explicitly been shown to trigger ferroptosis (e.g., in compensation experiments with ferroptosis inhibitors), even though many other natural products exist that induce cell death and/or interfere with pathways that are critical for ferroptosis. We also excluded natural products that employ multiple cell death programs, where in sum ferroptosis is only marginally involved.

Besides natural products, an increasing number of established anticancer drugs have been identified to induce ferroptotic cell death.^338^ However, the contribution of ferroptosis to their overall cytotoxic activity is often low (e.g., for cisplatin), with only a few exceptions. Ferroptosis is substantially induced by siramesine (sigma receptor agonist) in combination with lapatinib (an inhibitor of the tyrosine kinases EGFR and HER2),^339^ neratinib (a potent irreversible pan‐tyrosine kinase inhibitor),^340^ and apatinib (a selective inhibitor of the tyrosine kinase vascular endothelial growth factor receptor‐2).^341^ Moreover, multiple FDA‐approved drugs with recorded cytotoxic/anticancer properties like the hypoglycemic drug metformin^342^ or the antifungal drug itraconazole^343^ have been reported to induce ferroptosis in addition to their primary mode of action. Recent review articles give a comprehensive overview of the current knowledge in these fields.^344^, ^345^

### 9.1 Compounds that trigger iron oxidation

ROS formation (which is triggered by pleiotropic natural products) is not sufficient to induce ferroptosis,^88^, ^346^ and an increase of ROS levels or the inhibition of antioxidative enzymes might also be deleterious for noncancerous cells resulting in severe side‐effects during anticancer therapy. ROS inducers acting on ferrous iron and enhancing the Fenton reaction effectively trigger lipid peroxidation, preferentially induce ferroptosis as compared to other cell death programs, and might show selectivity for cancer cells due to higher iron levels in tumors as compared to healthy tissue.^124^, ^125^ Even general ROS inducers might have some preference for cancer cells, given their increased ROS tonus as compared to normal cells.^6^ Nevertheless, ROS‐inducing drug candidates have to be carefully evaluated for their cytotoxicity on normal cells, tissues, and organs.

#### 9.1.1 Plakinic acid (**96**) and its synthetic derivative FINO2 (**97**)

**Figure 10:**
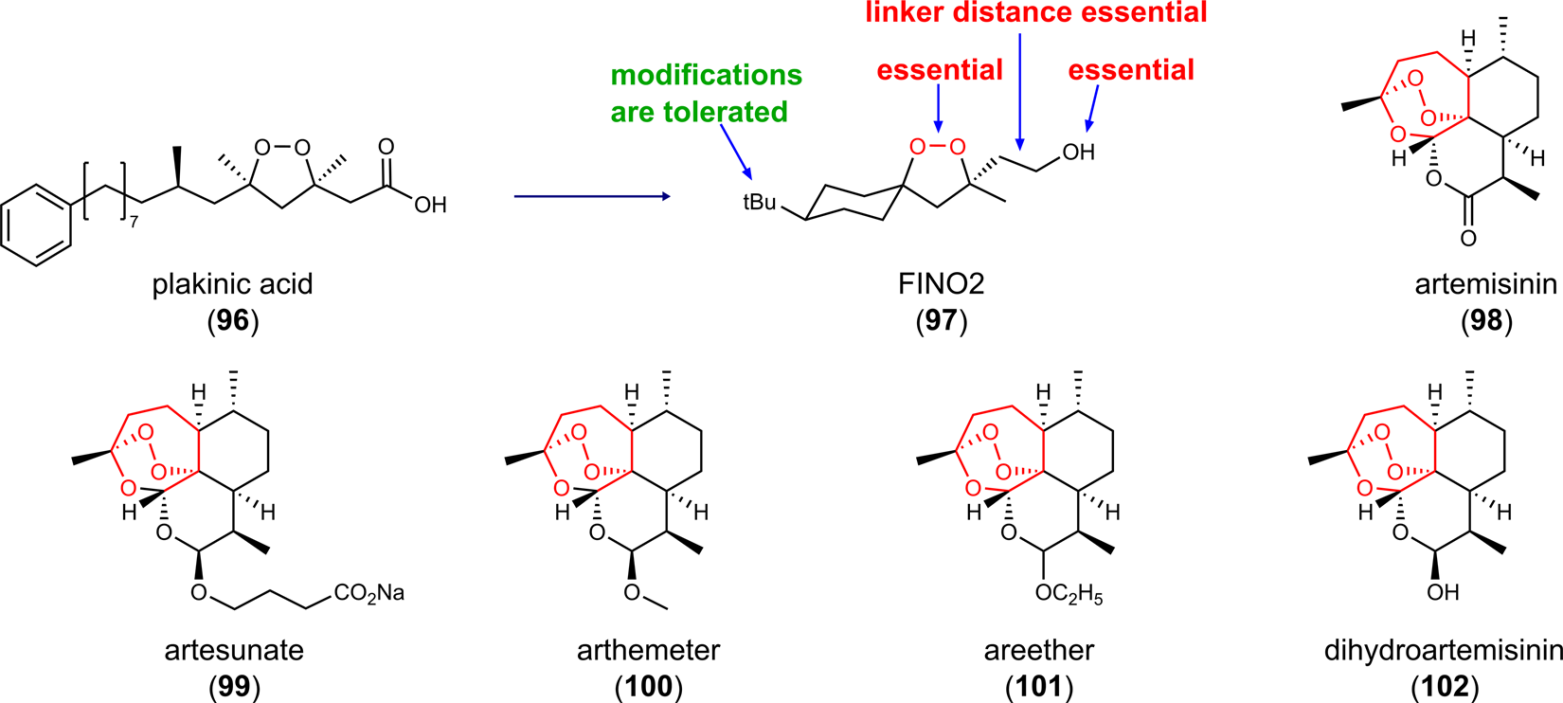
Inducers of (lipid) reactive oxygen species formation [Color figure can be viewed at wileyonlinelibrary.com]

The natural product **96** (Figure 10) found in sponges (*Plactortis species*) served as a lead scaffold for the synthesis of endoperoxides, yielding **97**.^337^ The dioxolane **97** induced lipid hydroperoxide generation and iron‐dependent cell death in various cancer cell lines, surpassing artemisinin, a naturally occurring endoperoxide described below.^347^ Ferroptosis inhibitors (ferrostatin‐1, liproxstatin‐1, DFO, baicalain, trolox) fully prevented cell death by **97** (2–10 µM) in human BJ‐ELR fibroblast cancer cells and human HT108 fibrosarcoma cells.^337^, ^347^ Neither apoptosis, necroptosis nor autophagy were triggered by the endoperoxide. Compound **97** oxidized ferrous iron, induced lipid peroxidation and lowered GPX4 activity through an unknown mechanism, without reducing GSH levels. Direct inhibition of GPX4 has been excluded, and also GPX4 protein expression was only slightly decreased.^337^, ^347^ Compound **97** effectively induced cell death in oncogenic, transformed human BJ‐ELR fibroblasts (EC_50_ = 2 µM),^337^ and cell lines with inactive or lost p53 gene, such as human NCI‐H522 and HOP‐92 NSCLC cells and HL‐60 leukemia cells (EC_50_ = 1.2–2.2 µM).^347^ Cancer cells seem to be more susceptible to **97** than normal cells, as suggested from the high effective concentrations required to induce cell death in immortalized (noncancerous) human BJ‐hTERT fibroblasts (noncytotoxic at 20 µM).^347^ Compound **97** requires both the endoperoxide and the proximate polar head group (alcohol) to induce cytotoxicity, and neither replacing the alcohol with nonpolar substituents nor increasing the distance between the alcohol and endoperoxide is tolerated (Figure 9).^337^ Modifications of the *tert*‐butyl moiety were instead accepted. These structural requirements suggest that **97** has to acquire a defined orientation at membrane interphases that place the endoperoxide in close proximity to the *bis*‐allylic double bonds of esterified PUFAs. It is tempting to speculate that **97** is anchored in cellular membranes and that the polar head group is orientated toward the aqueous phase while the lipophilic core extends toward the inner membrane, where the endoperoxide initiates the generation of hydroxyl radicals via the Fenton reaction.

#### 9.1.2 Artemisinin (**98**), its derivatives artesunate (**99**), arteether (**100**), artemether (**101**), and the metabolite, dihydroartemisinin (**102**)

The sesquiterpene lactone ***98*** (Figure 9) from *Artemisia annua* has been used in traditional Chinese medicine for two millennia and is FDA‐approved as antimalarial drug, as is artesunate, a derivative with improved water solubility.^10^, ^348^ The pharmacological activity of **98** and its derivatives have been intensively investigated during the last 2 decades, and several phase I/II trials have been conducted (www.clinicaltrial.gov, NCT02633098, NCT03093129, NCT00764036, NCT02353026, NCT02354534, NCT03792516, NCT03100045, NCT04098744, NCT02786589, NCT05478239, NCT02304289).^349^ These studies indicate prominent antitumoral efficacy combined with generally low toxicity and adverse effects and highlight the endoperoxide (1,2,4‐trioxane) as an essential pharmacophore.^348^ Interested readers are encouraged to consult a recent survey article that describes the potential of **98** in anticancer therapy.^348^, ^350^, ^351^

Here, we focus on mechanisms likely contributing to the proferroptotic activity of **98**. Compound **98** and multiple derivatives exhibit potent cytotoxic activity in cancer cell lines and tumor models, which was partially ascribed to the induction of ferroptotic cell death.^348^, ^352^, ^353^, ^354^, ^355^, ^356^ The antimalarial potential of **98** is linked to its ability to react with free ferrous ions.^348^ Most likely, the endoperoxide is cleaved by ferrous iron, leading to the formation of reactive free radicals via the Fenton reaction. The combination of **98** with ferrous iron accordingly increased the cytotoxic activity of cancer cells, as expected from an iron‐dependent mechanism.^352^ Further proof that labile iron plays an important role in the mechanism of **98** comes from the cotreatment with the iron chelator DFO that diminished the anticancer activity of **102**.^357^ Of note, compound **102** and its derivatives induced a plethora of other mechanisms involved in ferroptosis but also other forms of cell death, including DNA damage, modulation of gene expression, and interference with mTOR, NF‐κB, MAPK, and Wnt/β‐catenin signaling.^349^ Since many of this survival, oncogenic and stress‐adaptive pathways contribute to tumor resistance, polypharmacological inhibitors like **97** might be advantageous for the treatment of therapy‐resistant cancer.^348^ Along these lines, artesunate efficiently killed apoptosis‐resistant pancreatic ductal adenocarcinoma cells by triggering ferroptosis.^352^

The metabolite of **98**, compound **102**, was recently reported to upregulate GPX4 expression as part of an antiferroptotic feedback loop.^358^ Compound **102** activated PERK that led to an upregulation of ATF4 and subsequent expression of the heat‐shock protein HSPA5.^358^ Inhibition of PERK, ATF4, and HSPA5 (either using a pharmacological inhibitor or siRNA) substantially increased the sensitivity of cultured and grafted glioma cells to **102**, which indicates that targeting the PERK branch of the unfolded protein response might be an effective means to increase the vulnerability of tumors against ferroptosis induction by **98** and derivatives.

### 9.2 Modulators of cysteinyl–cystinyl homeostasis

A large variety of natural products suppresses redox‐dependent processes. Compounds that inactivate thioredoxin or inhibit thioredoxin reductases are described in detail in recent survey articles.^297^, ^359^, ^360^ Other natural products target GSH, PRDX1/2, GGT1, and further players in redox homeostasis.^361^ Thioredoxin reductases are, like GPX4, selenoproteins with a nucleophilic, active site selenocysteine, which forms covalent adducts with electrophilic compounds.^359^ Among the natural products that potently inhibit thioredoxin reductases are phenylpropanoids and polyphenols (e.g., **47** and curcuminoids),^362^ quinoids (e.g., plumbagin and **80**),^296^ terpenoids (e.g., **40, 59** and mitomycin),^184^, ^236^ and diverse other structural classes (e.g., gambogic acid).^297^ Note that distinct thioredoxin reductase inhibitors (**47**, the curcumin derivative **48, 80**) also activate NRF2 as described in Section 6.3. Thioredoxin reductase inhibition most likely involves covalent binding of the inhibitor to selenocysteine and/or cysteines of the active site, as originally proposed for **80** (see Section 7).^296^ Such electrophilic groups are also present in NRF2 activators that covalently bind to the NRF2 inhibitor KEAP1.^48^ It is, therefore, not surprising that compounds with electrophilic groups target both proteins, KEAP1 and thioredoxin reductase. Other factors involved in redox homeostasis, such as PRDXs and GGT1, also contain redox‐active cysteines, either in the active center or as redox sensors, which offer prominent sites for electrophilic attack, as exploited by **61** and **59** (see Section 6.3). The large majority of small molecules that target cysteinyl‐cystinyl homeostasis (**47, 48, 59, 61, 80**) also induce HO‐1 expression and are therefore listed in Sections 6.3 and 7. In this section, we discuss the thioredoxin‐targeting ferroptocide (**99**), which seems not to share this dual mechanism.

#### 9.2.1 Ferroptocide (**104**)

**Figure 11:**
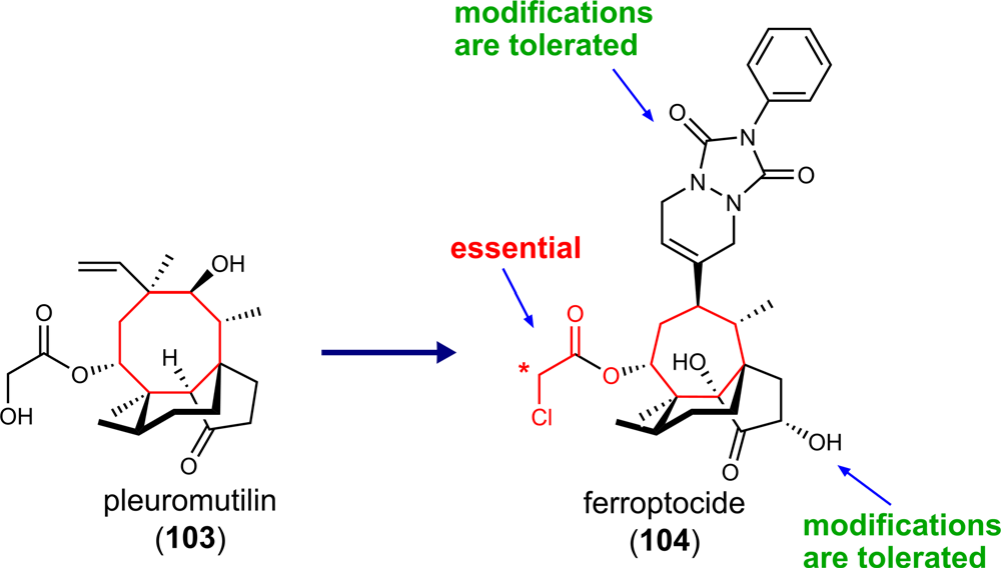
Ferroptosis‐inducing ferroptocide covalently targets thioredoxin [Color figure can be viewed at wileyonlinelibrary.com]

Applying the complexity‐to‐diversity (CtD) strategy to the diterpene natural product pleuromutilin (**103**) yielded compound **104** (Figure 11), a ferroptosis inducer with varying cytotoxic potency between cell lines (human ovarian ES‐2 cells: EC_50_ = 1.6 µM; MDA‐MB‐231: EC_50_ = 25 µM).^3^ The CtD strategy aims at producing complex and diverse compound libraries from natural products by ring distortion reactions that create novel scaffolds.^3^ SAR studies indicate that the electrophilic chloroacetoxy group is essential for the cytotoxic activity of **104**, whereas the secondary alcohol as well as N4 and N9 of the partially saturated benzo[*a*]triazole ring system are promising sites for structural optimization. Compound **104** selectively inactivated thioredoxin and increased membrane peroxidation in human ES‐2 ovarian clear cell carcinoma cells similar to thioredoxin silencing.^3^ Thioredoxins are important redox‐cofactors of the antioxidative defense system, which (i) lower the redox tonus, (ii) decrease oxidative protein modifications, and (iii) reduce cystine to cysteine for GSH biosynthesis.^147^ The five cysteines in thioredoxins represent possible sites for covalent binding of the reactive chloroacetoxy moiety of **104**, two of them form the redox‐active cysteine pair in the active center (Cys32 and Cys35).^3^ Site‐directed mutagenesis identified Cys32 and Cys35 as well as the adjacent cysteine Cys73 as sites of modification. Although the chloroacetoxy moiety of **104** resembles the chloroacetamide in GPX4 inhibitors (Section “GPX4 inhibitors”), compound **104** does not inhibit GPX4.^3^

Thioredoxin 1 and thioredoxin reductase 1 are upregulated in various forms of cancer^363^ and associated with excessive proliferation, metastasis, and protection from apoptosis.^360^ Moreover, there are several lines of evidence that the thioredoxin system is involved in drug resistance.^364^ Targeting the thioredoxin system thus seems to be another promising strategy to trigger ferroptosis and combat (therapy‐resistant) cancer.

### 9.3 Natural products that trigger ferroptosis through other mechanisms

#### 9.3.1 Salinomycin (**105**) and the derivative ironomycin (**106**)

**Figure 12:**
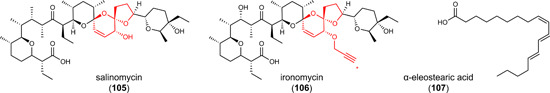
Increasing the lysosomal iron load or inducing spontaneous lipid peroxidation [Color figure can be viewed at wileyonlinelibrary.com]

The tricyclic spiroketal **105** (Figure 12) was originally isolated from *Streptomyces albus* and is a monovalent ionophor and antibiotic.^365^ Compound **105** (EC_50_ = 1 µM) and the more potent derivative **106** (EC_50_ = 0.1 µM) induced ferroptotic cell death in transformed human HMLER mammary epithelial cells (with high CD44 expression) but were hardly active on human primary breast cells. The cytotoxic effect was attenuated by ferrostatin‐1 but not by apoptosis (Z‐VAD‐FMK) or necroptosis inhibitors (necrostatin‐1). Compounds **105** and **106** were enriched in lysosomes and efficiently bound ferrous iron, which depleted iron in the cytosol. Enhanced lysosomal degradation of ferritin further increased the lysosomal iron load, and the free ferrous/ferric ions seemingly elevated ROS formation preferentially in lysosomes as well as cellular lipid peroxidation. Of note, both ionophores have a rather low affinity to ferrous iron and form iron complexes that are still Fenton‐active in contrast to the tight Fe^2+^ complexes of DFO and DFO that are redox‐inactive. Both, compounds **105** (3 mg/kg, ip, daily) and **106** (1 mg/kg, ip, daily) efficiently inhibited tumor growth in a Werner syndrome patient‐derived SV40 fibroblast cell xenograft model in mice.

#### 9.3.2 PUFAs and conjugated linolenic acids

PUFAs like dihomo‐γ‐linolenic acid (≥500 µM) and fragmented oxidized phosphatidylcholines (1–10 µM), for example, 1‐palmitoyl‐2‐(5'‐oxo‐valeroyl)‐*sn*‐glycero‐3‐phosphocholine, have been shown to induce ferroptosis in human fibrosarcoma cells^366^ and rat primary cardiomyocytes,^367^ respectively. Conjugated linolenic acid (CLA) with three conjugated double bonds, such as α‐eleostearic acid (**107**) (Figure 12), have antioxidative, antiatherogenic, antiobese, anti‐inflammatory, and antitumoral activities^368^ and are more effective in inducing ferroptosis than PUFAs with isolated double bonds. They are synthesized in high amounts in bitter gourd (*Momordica charantia*)^369^ and promegranate seeds^370^ and are also produced by distinct (intestinal) bacteria.^371^ Compound **107** is incorporated into triglycerides by ACSL1, increases lipid peroxidation, and induces cell death in many human cancer cell lines (>100 investigated), including triple‐negative breast cancer cells (EC_50_ = 10 µM).^101^ The mechanism by which triglyceride‐bound **107** causes cell death is not fully understood. It has been hypothesized that **107** undergoes spontaneous or enzymatic oxidation when its concentration exceeds a distinct threshold, which then propagates autooxidation by chain reaction that is facilitated by the conjugated system and potentially leads to polymerization. The anticancer activity of CLA was studied in an aggressive triple‐negative breast cancer orthotopic xenograft model, where compound **102**‐rich tung oil (100 µl, po, 5× per week) decreased tumor mass as well as lung metastatic invasion.

### 9.4 Natural products that trigger ferroptosis through unknown mechanisms

#### 9.4.1 Resibufogenin (**108**)

**Figure 13:**
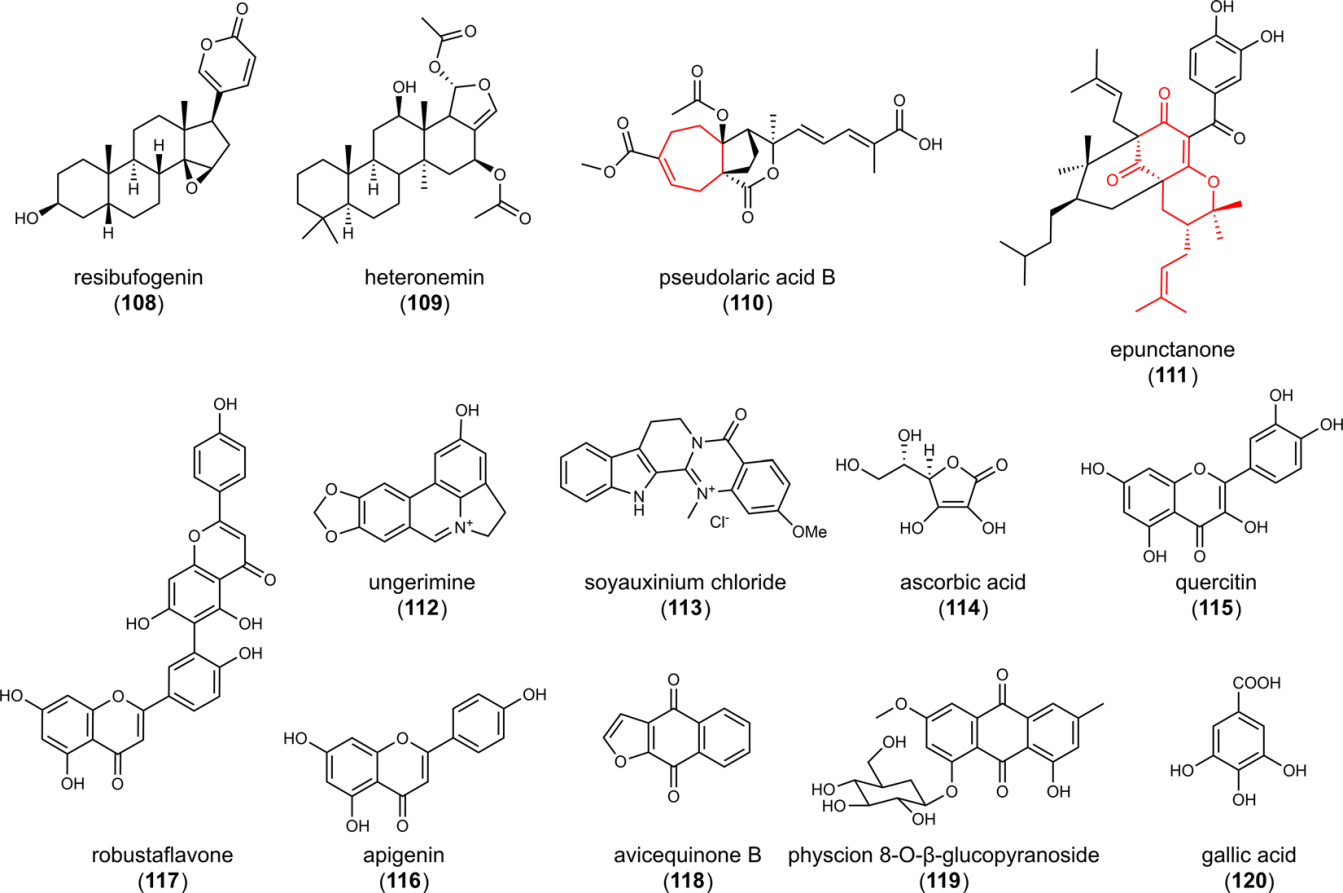
Inducers of (lipid) reactive oxygen species formation that trigger ferroptosis through less characterized mechanisms [Color figure can be viewed at wileyonlinelibrary.com]

The bufadienolide **108** (20–40 µM) (Figure 13) isolated from Asiatic toad (*Bufo gargarizans*) increased MDA, iron, and ROS levels, decreased GPX4 expression, reduced the availability of GSH and triggered ferroptosis in human HT29 and SW480 CRC cells.^372^ Administration of **108** (5 and 10 mg/kg, ip) to mice suppressed growth and metastasis of human SW480 CRC cells through RIP3‐mediated necroptosis in a xenograft model.^373^ Another study using this CRC cancer model reported that oxidative stress is induced and tumorigenicity is reduced at high dosage of **108** (80 mg/kg, injected in the tumor each day).^372^ It seems that **108** engages different cell death programs, and ferroptosis might not be the predominant form in vivo. Note that **108** has the same bufadienolide scaffold as **44**, a natural GPX4 inhibitor described in Section 6. Moreover, similar to **44**, it contains a highly reactive endoperoxide albeit at a different position.

#### 9.4.2 Heteronemin (**109**)

The pentacyclic triterpenoid **109** (Figure 13) found in the sponge *Hippospongia species* showed profound cytotoxicity in various cancer cell lines (EC_50_ = 0.1–2.4 µM).^374^, ^375^ Compound **109** induced caspase‐dependent apoptosis as well as ferroptosis.^375^ The latter was diminished by ferrostatin‐1 as well as liproxstatin‐1 and associated with reduced GPX4 protein levels.^375^ The development of **109** as a drug candidate was hampered by severe toxic effects in mice already at 1 mg/kg (application route not given) and a lethal dose of 5 mg/kg (application route not given).

#### 9.4.3 Pseudolaric acid A (**110**)

The diterpene acid **110** (2 µM, (Figure 13) from the root and trunk bark of *Pseudolarix amabilis* ^376^ induced lipid peroxidation and cell death (EC_50_ = 1 µM) in rat C6 and human SHG‐44 glioma cells. MDA generation was prevented by ferrostatin‐1 or the iron chelator DFO. Compound **110** (10 mg/kg, daily for 8 days, ip) was also active on grafted C6 glioma cells in mice and markedly reduced the tumor volume (eightfold).^376^ Compound **110** decreased SLC7A11, cysteine, and GSH levels enhanced the expression of NOX4, which reduces oxygen to superoxide radical anions, rose the labile iron pool, and surprisingly upregulated GPX4 in diverse rat and human glioma cell lines (C6, SHG‐44, U87, U251) and/or grafted tumors in mice. Note that an increase in GPX4 expression was also observed for the artemisinin metabolite **102**.^358^ In addition, compound **110** enhanced p53 expression and phosphorylation but showed similar cytotoxicity and ferroptotic signatures (SLC7A11, GPX4 and transferrin expression) in p53 wild‐type (U87) and p53 mutant (U251) cells,^376^ which questions a major role of p53 in compound **110**‐induced ferroptosis.

#### 9.4.4 Epunctanone (**111**), ungerimine (**112**), and soyauxinium chloride (**113**)

The benzophenone **111** from *Garcinia epunctata* and the two alkaloids **112** from *Crinum zeylanicum* and **113** from *Araliopsis soyauxii* (Figure 13) have recently been identified to induce ferroptosis.^279^, ^280^, ^377^, ^378^, ^379^ These compounds have cytotoxic activity (EC_50_ = 3.6–111 µM) against diverse cancer cell lines from the breast, colon, lymphatic tissue, brain, and skin, which includes drug‐resistant phenotypes that either overexpress ABC transporters (P‐glycoprotein and BCRP), have activating mutations in EGFR or B‐RAF, or have p53 deleted. The iron chelator DFO and the lipid peroxidation inhibitor ferrostatin‐1 decreased the cytotoxic effects of the natural products, which suggests a major role of ferroptosis in cell death induction. Whether the observed increase in intracellular ROS levels is associated with enhanced membrane peroxidation was not investigated. Other forms of cell death were triggered likewise, albeit with variable profiles for the different natural products. Compound **111** induced apoptosis (necroptosis and autophagy were not investigated),^280^, ^377^ **112** activated apoptosis and autophagy,^378^ and **113** initiated apoptosis as well as necroptosis.^279^, ^379^ Note that **111** shares a prenylated bicyclic ring system with **87** (see Section 7) and that we proposed above that **87** modulates NRF2 activation based on changes in NRF2 target gene expression. Respective mechanistic hints are lacking for **113**.

#### 9.4.5 Vitamin C (**114**)

Preclinical studies suggest that the antioxidant **114** (Figure 13) also acts as pro‐oxidant, disturbs the cellular redox balance, and has anticancer activity at supraphysiological concentrations in the millimolar range.^380^ Compound **114** (1 mM) exhibits cytotoxic, antiproliferative activity in human ATC 8505 C thyroid carcinoma cells,^381^ which was diminished by the iron chelator DFO but neither by apoptosis (Z‐VAD‐FMK) nor necroptosis inhibitors (necrostatin‐1).^381^ Mechanistically, GPX4 levels were decreased and MDA levels as well as the labile iron pool increased, which was ascribed to ferritinophagy and subsequent FTH degradation.

#### 9.4.6 Flavonoids—quercetin (**115**) and apigenin (**116**)

The flavonoids **115** and **116** (Figure 13) are cytotoxic for diverse cancer cell lines at micromolar concentrations, including human HepG2 and HepB3 hepatocarcinoma, HTC116 CRC and MDA‐MB‐231 breast cancer cells (EC_50_ < 100 µM).^382^, ^383^ Compound **115** (50 µM) enhanced lipid peroxidation and elevated cellular iron levels, which was prevented by the ferroptosis inhibitors DFO, ferrostatin‐1, and liproxstatin‐1 as well as the autophagy inhibitor bafilomycin A1.^382^ Cellular iron accumulation was ascribed to an activation of the transcription factor EB that leads to lysosomal degradation of ferritin (ferritinophagy). Moreover, compound **115** decreased GPX4 protein expression. The structurally related flavonoid **116** induced ferroptotic cell death in multiple myeloma cell lines (EC_50_ = 10–30 µM) along with apoptosis and autophagy.^383^

#### 9.4.7 Biflavonoids—robustaflavone A (**117**)

Several biflavonoids from *Selaginella trichoclada* (used in traditional Chinese medicine to treat jaundice and burns) induced cell death in human MCF‐7 breast cancer cells, with **117** (Figure 13) being most potent (EC_50_ = 12 µM).^384^ Cytotoxic effects of **117** were associated with enhanced lipid hydroperoxide formation and slightly diminished by ferrostatin‐1 or DFO, which hints toward a limited involvement of ferroptosis in cell death induction. Compound **117** downregulated Nedd4, an E3‐ubiquitin ligase that subjects voltage‐dependent anion channel 2 (VDAC2) to proteasomal degradation, and elevated VDAC2 protein levels, seemingly both via impaired protein degradation and enhanced mRNA expression. VDAC2 is an outer membrane pore in mitochondria and has recently been shown to increase the sensitivity of cancer cells to system X_c_^−^ inhibition.^385^

Due to the structural similarity to **94**, we proposed in Section 8 that the biflavonoid **95** inhibits NRF2 signaling, and compound **117** only differs from **95** by the site of oxidative coupling. While the apigenin moieties of **95** are linked between C‐3 of the hydroxyphenyl ring and C‐8 of chromene, the bond between the two apigenins is formed between C‐3 and C‐6 in **117**. This apparent small difference has strong impact on the 3D‐structure of the molecules, resulting either in a compact (**95**) or curved, rod‐like structure (**117**). Since the knowledge about the ferroptotic mechanisms of **117** are limited, we decided not to group this compound together with other biflavonoids in Section 7, although we cannot exclude a common mode of action.

#### 9.4.8 Physcion 8‐O‐β‐glucopyranoside (**118**)

The anthraquinone glycoside **118** (Figure 13) isolated from *Rumex japonicus* possesses antiapoptotic, antiproliferative and antimetastatic properties.^386^ Compound **118** elevated cellular iron and MDA levels and induces death in human MGC‐803 and MKN‐45 gastric carcinoma cells (EC_50_ = 40 µM),^387^ which was prevented by ferrostatin‐1 but not by the apoptosis inhibitor Z‐VAD‐FMK or the necroptosis inhibitor necrostatin‐1.^387^ Knockdown studies indicate that ferroptosis induction by **118** depends on miR‐103a‐3p upregulation and subsequent expression of GLS2. The mitochondrial GLS2 converts glutamine to glutamate, is involved in cancer metabolic reprogramming and has been shown to promote lipid peroxidation and ferroptosis.^388^

#### 9.4.9 Napthoquinone—avicequinone B (**119**)

The naphthoquinone **119** (Figure 13) from the mangrove tree *Avicennia alba* reduced the viability of human breast, colorectal, and lung adenocarcinoma cells (EC_50_ = 3–12 µM).^389^ Transcriptome analysis revealed that **119** regulates ferroptosis‐related genes (e.g., SLC7A11 and GCL), however, in the opposite direction as expected for ferroptosis induction. The authors speculate about unknown targets and feedback mechanisms.^389^

#### 9.4.10 Gallic acid (**120**)

3,4,5‐Trihydroxybenzoic acid (**120**; Figure 13) is prevalent in *Quercus species* and has recently been proposed to induce ferroptosis besides apoptosis and necroptosis at high concentrations (300 µM) in human HeLa epithelial adenocarcinoma cells, H446 lung carcinoma epithelial cells, SH‐SY5Y neuroblastoma cells, MDA‐MB‐231 breast cancer cells and A375 melanoma cancer cells.^390^, ^391^ The contribution of ferroptosis to cell death induction remains enigmatic. On the one hand, gallic acid decreased the mRNA expression of distinct ferroptosis‐related genes (e.g., GPX4 and p53),^390^ and it was speculated based on docking studies that **120** directly binds to several key factors in ferroptosis, including GPX4, p53 and NRF2.^390^ The iron chelator DFO diminished the cytotoxic effects of **120**.^392^ On the other hand, **120** did not evoke substantial lipid peroxidation, and the lipophilic radical trap ferrostatin‐1 failed to prevent cell death.^392^ Together, **120** induces cell death through multiple mechanisms and further studies are necessary to define the degree, to which ferroptosis is involved.

#### 9.4.11 Comparative discussion

An increasing number of compounds have been identified during recent years that induce membrane peroxidation and promote ferroptotic cell death independent from GPX4 and system X_c_^−^ inhibition. Many of them are natural products or bioinspired small molecules that induce besides ferroptosis also other forms of PD like apoptosis, necroptosis or autophagy. Such polypharmacological molecules might have advantages regarding clinical efficacy in anticancer therapy by hindering the tumors to develop escape routes and acquire resistance mechanisms.^7^, ^393^ On the other hand, increasing evidence suggests that compounds preferentially inducing ferroptosis might have advantages in discriminating between cancer and nontransformed cells, which expands to diverse therapy‐resistant phenotypes and is important for safety.

Major ferroptosis‐inducing mechanisms exploited by the natural products addressed in this chapter involve effects on (i) iron oxidation and the Fenton reaction (**97**, artemisinins), (ii) the thioredoxin system (**104**), (iii) spontaneous lipid autoxidation (CLA‐like **107**), (iv) iron storage and transport (this section: **105, 106** and **65, 66** in Section 6.3), and (v) glutaminolysis (**119**). It should be noted that many of the here described natural products have promiscuous activities, which might essentially add to their partly encouraging activity against (therapy‐resistant) cancer but hamper conclusions about the individual targets that contribute to cell death induction. For iron‐oxidizing endoperoxides (**97**, artemisinins), it is well documented that they drive lipid hydroperoxide formation through the Fenton reaction,^337^ though also here the exact interactions and mechanisms of subcellular membrane peroxidation are not always fully understood. For ferroptosis inducers that indirectly stimulate the accumulation of lipid hydroperoxides, it is even more difficult to assign which of the proposed mechanisms (i) have pharmacological relevance, (ii) interfere with the sensitive redox balance of membranes and (iii) contribute to ferroptosis induction under physiological settings. Functional studies based on compensation experiments that link proposed targets with cell death induction were successfully conducted only for few studies and are inconclusive (**104**)^3^ or lacking for others (e.g., **110**).^376^ For many further small molecules, the molecular targets are still enigmatic or, if known at all, not obviously linked to ferroptosis.

Among the diverse compounds in this section, two major groups can be classified based on their structure: (1) the artemisinins **98–102** and the compounds **103, 110** (this section), and **54** (see Section 6.3) share an eight‐membered ring system, and 2) compounds **105** and **106** (this section) as well as **65, 66, 67**, and **68** (see Section 6.3) have a spiroketal or an aza‐analogous spiroketal scaffold. The mechanisms by which the former induce ferroptosis are diverse and range from triggering the Fenton reaction to inactivating thioredoxin, whereas the spiroketals **65–68, 105**, and **106** have been reported to interfere with iron metabolism. Further studies might help to better understand the bioactivity and clinical potential of these anticancer drug candidates and potentially provide novel insights into the regulation of ferroptosis‐protective pathways.

## 10 CONCLUSION AND FUTURE PERSPECTIVES

Long before system X_c_^−^ has been identified as a key player in ferroptosis, inhibitors have been developed and the first anticancer drug candidate entered clinical trials. The interest in small molecules that target system X_c_^−^ rapidly increased after iron‐dependent lipid peroxidation has been recognized as a central part of a strictly regulated cell death program, and the term ferroptosis has been coined. Inhibitors of ferroptosis promise protection against neurodegenerative diseases,^20^, ^21^, ^394^ whereas ferroptosis inducers are directed against hyperproliferative disorders like cancer.^10^, ^58^, ^329^ Research in the latter field experienced a boost after aggressive, metastatic, and therapy‐resistant cancer cells were found to be highly sensitive to ferroptosis.^1^ GPX4, the only GPX isoenzyme that detoxifies lipid hydroperoxides, was early considered a promising target to trigger ferroptosis in tumors. However, major advances in drug development were hampered for several reasons, one of them being the lack of a clearly defined inhibitor binding pocket at the enzyme. Major breakthroughs in GPX4 inhibitor development may be expected from ligands combining specific noncovalent inhibition with an electrophilic warhead that covalently binds to the active site selenocysteine.

Direct GPX4 and system X_c_^−^ inhibitors are nowadays dominated by screening hits from synthetic compound libraries and structurally optimized derivatives, but early system X_c_^−^ inhibitors (**1** and **2**) were actually plant secondary metabolites^144^ and most NRF2 inhibitor/activators still originate from natural sources or are bioinspired. The high number of ferroptosis‐inducing natural products is not surprising when considering that ferroptosis is an ancient, evolutionally conserved cell death program that is shared by plants and animals.^395^

Triggering ferroptosis is generally considered an effective means to induce cell death in therapy‐resistant cancer cells.^1^ Among the ferroptosis inducers that efficiently discriminate between cancer and nontransformed cells are small molecules that induce lipid hydroperoxide formation either by (i) inhibiting GPX4, system X_c_^−^ or the thioredoxin/thioredoxin reductase system, (ii) inactivating NRF2, (iii) repressing GXP4 in combination with inducing HO‐1 expression, or (iv) triggering iron oxidation and the Fenton reaction. For example, the NRF2 modulators **94** and **56** act synergistically with conventional anticancer drugs and show high cytotoxic activity against drug‐resistant cancer cells, in particular toward those carrying mutations within the NRF2 axis. On the other hand, activation of the NRF2/HO‐1 axis seems to be an effective strategy, when combined with diminished GPX4 expression. Further studies are necessary to explore the functional links.

Overall, ferroptotic cell death is heterogenic and highly context‐dependent, which demands for personalized ferroptosis‐related anticancer strategies. Factors that shape ferroptosis sensitivity are the fatty acid composition of (sub)cellular membranes (in particular the balance between esterified PUFAs and MUFAs) and the capacity of antioxidant systems, including GPX4/GSH, FSP1/DHODH‐CoQ10, and GCH1/BH4. The number of known regulatory proteins in ferroptosis is dynamically increasing, and links to potential drug targets are emerging. For example, KEAP1 deletion (and thereby activation of NRF2) dramatically reduced GPX4 levels in human H1299 lung cancer cells but, in contrast to GPX4 deficiency, neither raised lipid peroxidation nor enhanced cell death. The failure of KEAP1 disruption to induce ferroptosis was ascribed to a concomitant induction of FSP1 that compensates for the loss of GPX4.^50^ On the other hand, cancer‐related processes, like EMT or metabolic reprogramming (e.g., by upregulating AR), are located at the crossroad to ferroptosis and regulate hallmarks in ferroptosis, such as the membrane PUFA proportion or the availability of GPX4.^51^, ^203^ These cancer‐specific discrepancies render distinct (therapy‐resistant) tumors more vulnerable toward ferroptosis‐inducing strategies than others, though not necessarily always toward the same targets or target combinations.^199^, ^202^, ^203^ The recent progress in the understanding of the complex defense network against ferroptosis now provides access to pleiotropic putative targets for ferroptosis‐triggering small molecules. Whether these points of attack are already exploited by some of the here described ferroptosis inducers and whether their pharmacological manipulation is promising in light of antitumoral efficacy and safety, requires further investigation.

We hypothesize that ferroptosis inducers limit the capacity of cancer cells to hijack their targets and upregulate them under oxidative stress. In addition, many of these small molecules have multiple activities that likely add to the overall anticancer properties and might help to overcome tumor resistance. The exact mechanisms behind are largely obscure, as are the consequences on the tumor microenvironment, immune surveillance, stemness, vasculation, metastasis, energy metabolism, membrane composition as well as the redox lipidome; the latter requiring sensitive, high‐end mass spectrometric redox lipidomics.^396^

Classical strategies employed in drug design require the knowledge of defined targets as well as structural information about the binding sites and can therefore not easily be applied to ferroptosis‐inducing small molecules. GPX4 lacks a classical binding pocket, NRF2 is mainly regulated via redox‐dependent processes and specific target sites are absent or not defined, and the molecular targets are unknown for many other reported ferroptosis inducers. Future studies are needed to expand our current understanding of redox sensors and redox‐controlled mechanisms in ferroptosis. The increasing awareness that HO‐1 plays a central role in NRF2‐dependent ferroptosis is an important step in this direction. Such insights will be the catalyst to further accelerate the discovery, design, and rational optimization of ferroptosis‐modulating small molecules that specifically interfere with single or multiple key targets in ferroptosis and potentially other cell death pathways.

## CONFLICT OF INTEREST

The authors declare no conflict of interest.

## Supporting information

Supporting information.

## Data Availability

Data sharing is not applicable to this article as no new data were created or analyzed in this study.
