# Supplementary material for: Ferroptosis‐modulating small molecules for targeting drug‐resistant cancer: Challenges and opportunities in manipulating redox signaling
Source: Med Res Rev. 2023 Jan 19;43(3):614–82. doi: 10.1002/med.21933 (PMC10947485; doi:10.1002/med.21933)
Supplement: Supplementary file 1 — Supporting information. [file MED-43-614-s001.pdf]

IUCr Journals

Terms and conditions of use of the article titled

Crystal structures of the selenoprotein glutathione peroxidase 4 in its apo form and in complex with the covalently bound inhibitor ML162

[Moosmayer *et al.* (2021). *Acta Cryst.* D**77**, 237-248
https://doi.org/10.1107/S2059798320016125]

Permission to reproduce or reuse in whole or part the above article is granted to

Solveigh Koeberle

provided that the reused material is accompanied by an attribution statement according the terms of the open-access licence under which the original article was published, and that permission has been obtained from any other organizations or individuals where any parts of the material are subject to explicit statements of copyright or prior reproduction permission from such third parties.

Peter Strickland
Executive Managing Editor, IUCr Journals
